# The whole blood transcriptional regulation landscape in 465 COVID-19 infected samples from Japan COVID-19 Task Force

**DOI:** 10.1038/s41467-022-32276-2

**Published:** 2022-08-22

**Authors:** Qingbo S. Wang, Ryuya Edahiro, Ho Namkoong, Takanori Hasegawa, Yuya Shirai, Kyuto Sonehara, Hiromu Tanaka, Ho Lee, Ryunosuke Saiki, Takayoshi Hyugaji, Eigo Shimizu, Kotoe Katayama, Masahiro Kanai, Tatsuhiko Naito, Noah Sasa, Kenichi Yamamoto, Yasuhiro Kato, Takayoshi Morita, Kazuhisa Takahashi, Norihiro Harada, Toshio Naito, Makoto Hiki, Yasushi Matsushita, Haruhi Takagi, Masako Ichikawa, Ai Nakamura, Sonoko Harada, Yuuki Sandhu, Hiroki Kabata, Katsunori Masaki, Hirofumi Kamata, Shinnosuke Ikemura, Shotaro Chubachi, Satoshi Okamori, Hideki Terai, Atsuho Morita, Takanori Asakura, Junichi Sasaki, Hiroshi Morisaki, Yoshifumi Uwamino, Kosaku Nanki, Sho Uchida, Shunsuke Uno, Tomoyasu Nishimura, Takashri Ishiguro, Taisuke Isono, Shun Shibata, Yuma Matsui, Chiaki Hosoda, Kenji Takano, Takashi Nishida, Yoichi Kobayashi, Yotaro Takaku, Noboru Takayanagi, Soichiro Ueda, Ai Tada, Masayoshi Miyawaki, Masaomi Yamamoto, Eriko Yoshida, Reina Hayashi, Tomoki Nagasaka, Sawako Arai, Yutaro Kaneko, Kana Sasaki, Etsuko Tagaya, Masatoshi Kawana, Ken Arimura, Kunihiko Takahashi, Tatsuhiko Anzai, Satoshi Ito, Akifumi Endo, Yuji Uchimura, Yasunari Miyazaki, Takayuki Honda, Tomoya Tateishi, Shuji Tohda, Naoya Ichimura, Kazunari Sonobe, Chihiro Tani Sassa, Jun Nakajima, Yasushi Nakano, Yukiko Nakajima, Ryusuke Anan, Ryosuke Arai, Yuko Kurihara, Yuko Harada, Kazumi Nishio, Tetsuya Ueda, Masanori Azuma, Ryuichi Saito, Toshikatsu Sado, Yoshimune Miyazaki, Ryuichi Sato, Yuki Haruta, Tadao Nagasaki, Yoshinori Yasui, Yoshinori Hasegawa, Yoshikazu Mutoh, Tomoki Kimura, Tomonori Sato, Reoto Takei, Satoshi Hagimoto, Yoichiro Noguchi, Yasuhiko Yamano, Hajime Sasano, Sho Ota, Yasushi Nakamori, Kazuhisa Yoshiya, Fukuki Saito, Tomoyuki Yoshihara, Daiki Wada, Hiromu Iwamura, Syuji Kanayama, Shuhei Maruyama, Takashi Yoshiyama, Ken Ohta, Hiroyuki Kokuto, Hideo Ogata, Yoshiaki Tanaka, Kenichi Arakawa, Masafumi Shimoda, Takeshi Osawa, Hiroki Tateno, Isano Hase, Shuichi Yoshida, Shoji Suzuki, Miki Kawada, Hirohisa Horinouchi, Fumitake Saito, Keiko Mitamura, Masao Hagihara, Junichi Ochi, Tomoyuki Uchida, Rie Baba, Daisuke Arai, Takayuki Ogura, Hidenori Takahashi, Shigehiro Hagiwara, Genta Nagao, Shunichiro Konishi, Ichiro Nakachi, Koji Murakami, Mitsuhiro Yamada, Hisatoshi Sugiura, Hirohito Sano, Shuichiro Matsumoto, Nozomu Kimura, Yoshinao Ono, Hiroaki Baba, Yusuke Suzuki, Sohei Nakayama, Keita Masuzawa, Shinichi Namba, Takayuki Shiroyama, Yoshimi Noda, Takayuki Niitsu, Yuichi Adachi, Takatoshi Enomoto, Saori Amiya, Reina Hara, Yuta Yamaguchi, Teruaki Murakami, Tomoki Kuge, Kinnosuke Matsumoto, Yuji Yamamoto, Makoto Yamamoto, Midori Yoneda, Kazunori Tomono, Kazuto Kato, Haruhiko Hirata, Yoshito Takeda, Hidefumi Koh, Tadashi Manabe, Yohei Funatsu, Fumimaro Ito, Takahiro Fukui, Keisuke Shinozuka, Sumiko Kohashi, Masatoshi Miyazaki, Tomohisa Shoko, Mitsuaki Kojima, Tomohiro Adachi, Motonao Ishikawa, Kenichiro Takahashi, Takashi Inoue, Toshiyuki Hirano, Keigo Kobayashi, Hatsuyo Takaoka, Kazuyoshi Watanabe, Naoki Miyazawa, Yasuhiro Kimura, Reiko Sado, Hideyasu Sugimoto, Akane Kamiya, Naota Kuwahara, Akiko Fujiwara, Tomohiro Matsunaga, Yoko Sato, Takenori Okada, Yoshihiro Hirai, Hidetoshi Kawashima, Atsuya Narita, Kazuki Niwa, Yoshiyuki Sekikawa, Koichi Nishi, Masaru Nishitsuji, Mayuko Tani, Junya Suzuki, Hiroki Nakatsumi, Takashi Ogura, Hideya Kitamura, Eri Hagiwara, Kota Murohashi, Hiroko Okabayashi, Takao Mochimaru, Shigenari Nukaga, Ryosuke Satomi, Yoshitaka Oyamada, Nobuaki Mori, Tomoya Baba, Yasutaka Fukui, Mitsuru Odate, Shuko Mashimo, Yasushi Makino, Kazuma Yagi, Mizuha Hashiguchi, Junko Kagyo, Tetsuya Shiomi, Satoshi Fuke, Hiroshi Saito, Tomoya Tsuchida, Shigeki Fujitani, Mumon Takita, Daiki Morikawa, Toru Yoshida, Takehiro Izumo, Minoru Inomata, Naoyuki Kuse, Nobuyasu Awano, Mari Tone, Akihiro Ito, Yoshihiko Nakamura, Kota Hoshino, Junichi Maruyama, Hiroyasu Ishikura, Tohru Takata, Toshio Odani, Masaru Amishima, Takeshi Hattori, Yasuo Shichinohe, Takashi Kagaya, Toshiyuki Kita, Kazuhide Ohta, Satoru Sakagami, Kiyoshi Koshida, Kentaro Hayashi, Tetsuo Shimizu, Yutaka Kozu, Hisato Hiranuma, Yasuhiro Gon, Namiki Izumi, Kaoru Nagata, Ken Ueda, Reiko Taki, Satoko Hanada, Kodai Kawamura, Kazuya Ichikado, Kenta Nishiyama, Hiroyuki Muranaka, Kazunori Nakamura, Naozumi Hashimoto, Keiko Wakahara, Sakamoto Koji, Norihito Omote, Akira Ando, Nobuhiro Kodama, Yasunari Kaneyama, Shunsuke Maeda, Takashige Kuraki, Takemasa Matsumoto, Koutaro Yokote, Taka-Aki Nakada, Ryuzo Abe, Taku Oshima, Tadanaga Shimada, Masahiro Harada, Takeshi Takahashi, Hiroshi Ono, Toshihiro Sakurai, Takayuki Shibusawa, Yoshifumi Kimizuka, Akihiko Kawana, Tomoya Sano, Chie Watanabe, Ryohei Suematsu, Hisako Sageshima, Ayumi Yoshifuji, Kazuto Ito, Saeko Takahashi, Kota Ishioka, Morio Nakamura, Makoto Masuda, Aya Wakabayashi, Hiroki Watanabe, Suguru Ueda, Masanori Nishikawa, Yusuke Chihara, Mayumi Takeuchi, Keisuke Onoi, Jun Shinozuka, Atsushi Sueyoshi, Yoji Nagasaki, Masaki Okamoto, Sayoko Ishihara, Masatoshi Shimo, Yoshihisa Tokunaga, Yu Kusaka, Takehiko Ohba, Susumu Isogai, Aki Ogawa, Takuya Inoue, Satoru Fukuyama, Yoshihiro Eriguchi, Akiko Yonekawa, Keiko Kan-o, Koichiro Matsumoto, Kensuke Kanaoka, Shoichi Ihara, Kiyoshi Komuta, Yoshiaki Inoue, Shigeru Chiba, Kunihiro Yamagata, Yuji Hiramatsu, Hirayasu Kai, Koichiro Asano, Tsuyoshi Oguma, Yoko Ito, Satoru Hashimoto, Masaki Yamasaki, Yu Kasamatsu, Yuko Komase, Naoya Hida, Takahiro Tsuburai, Baku Oyama, Minoru Takada, Hidenori Kanda, Yuichiro Kitagawa, Tetsuya Fukuta, Takahito Miyake, Shozo Yoshida, Shinji Ogura, Shinji Abe, Yuta Kono, Yuki Togashi, Hiroyuki Takoi, Ryota Kikuchi, Shinichi Ogawa, Tomouki Ogata, Shoichiro Ishihara, Arihiko Kanehiro, Shinji Ozaki, Yasuko Fuchimoto, Sae Wada, Nobukazu Fujimoto, Kei Nishiyama, Mariko Terashima, Satoru Beppu, Kosuke Yoshida, Osamu Narumoto, Hideaki Nagai, Nobuharu Ooshima, Mitsuru Motegi, Akira Umeda, Kazuya Miyagawa, Hisato Shimada, Mayu Endo, Yoshiyuki Ohira, Masafumi Watanabe, Sumito Inoue, Akira Igarashi, Masamichi Sato, Hironori Sagara, Akihiko Tanaka, Shin Ohta, Tomoyuki Kimura, Yoko Shibata, Yoshinori Tanino, Takefumi Nikaido, Hiroyuki Minemura, Yuki Sato, Yuichiro Yamada, Takuya Hashino, Masato Shinoki, Hajime Iwagoe, Hiroshi Takahashi, Kazuhiko Fujii, Hiroto Kishi, Masayuki Kanai, Tomonori Imamura, Tatsuya Yamashita, Masakiyo Yatomi, Toshitaka Maeno, Shinichi Hayashi, Mai Takahashi, Mizuki Kuramochi, Isamu Kamimaki, Yoshiteru Tominaga, Tomoo Ishii, Mitsuyoshi Utsugi, Akihiro Ono, Toru Tanaka, Takeru Kashiwada, Kazue Fujita, Yoshinobu Saito, Masahiro Seike, Hiroko Watanabe, Hiroto Matsuse, Norio Kodaka, Chihiro Nakano, Takeshi Oshio, Takatomo Hirouchi, Shohei Makino, Moritoki Egi, Yosuke Omae, Yasuhito Nannya, Takafumi Ueno, Tomomi Takano, Kazuhiko Katayama, Masumi Ai, Atsushi Kumanogoh, Toshiro Sato, Naoki Hasegawa, Katsushi Tokunaga, Makoto Ishii, Ryuji Koike, Yuko Kitagawa, Akinori Kimura, Seiya Imoto, Satoru Miyano, Seishi Ogawa, Takanori Kanai, Koichi Fukunaga, Yukinori Okada

**Affiliations:** 1 Department of Statistical Genetics, Osaka University Graduate School of Medicine, Suita, Japan; 2 Laboratory of Statistical Immunology, Immunology Frontier Research Center (WPI-IFReC), Osaka University, Suita, Japan; 3 Department of Respiratory Medicine and Clinical Immunology, Osaka University Graduate School of Medicine, Suita, Japan; 4 Department of Infectious Diseases, Keio University School of Medicine, Tokyo, Japan; 5 M&D Data Science Center, Tokyo Medical and Dental University, Tokyo, Japan; 6 Integrated Frontier Research for Medical Science Division, Institute for Open and Transdisciplinary Research Initiatives, Osaka University, Suita, Japan; 7 Division of Pulmonary Medicine, Department of Medicine, Keio University School of Medicine, Tokyo, Japan; 8 Department of Pathology and Tumor Biology, Kyoto University, Kyoto, Japan; 9 Division of Health Medical Intelligence, Human Genome Center, the Institute of Medical Science, the University of Tokyo, Tokyo, Japan; 10 Department of Biomedical Informatics, Harvard Medical School, Boston, MA USA; 11 Department of Otorhinolaryngology-Head and Neck Surgery, Osaka University Graduate School of Medicine, Suita, Japan; 12 Department of Pediatrics, Osaka University Graduate School of Medicine, Suita, Japan; 13 Department of Immunopathology, Immunology Frontier Research Center (WPI-IFReC), Osaka University, Suita, Japan; 14 Department of Respiratory Medicine, Juntendo University Faculty of Medicine and Graduate School of Medicine, Tokyo, Japan; 15 Department of General Medicine, Juntendo University Faculty of Medicine and Graduate School of Medicine, Tokyo, Japan; 16 Department of Emergency and Disaster Medicine, Juntendo University Faculty of Medicine and Graduate School of Medicine, Tokyo, Japan; 17 Department of Cardiovascular Biology and Medicine, Juntendo University Faculty of Medicine and Graduate School of Medicine, Tokyo, Japan; 18 Department of Internal Medicine and Rheumatology, Juntendo University Faculty of Medicine and Graduate School of Medicine, Tokyo, Japan; 19 Atopy (Allergy) Research Center, Juntendo University Graduate School of Medicine, Tokyo, Japan; 20 Department of Emergency and Critical Care Medicine, Keio University School of Medicine, Tokyo, Japan; 21 Department of Anesthesiology, Keio University School of Medicine, Tokyo, Japan; 22 Department of Laboratory Medicine, Keio University School of Medicine, Tokyo, Japan; 23 Division of Gastroenterology and Hepatology, Department of Medicine, Keio University School of Medicine, Tokyo, Japan; 24 Keio University Health Center, Tokyo, Japan; 25 Department of Respiratory Medicine, Saitama Cardiovascular and Respiratory Center, Kumagaya, Japan; 26 JCHO (Japan Community Health care Organization) Saitama Medical Center, Internal Medicine, Saitama, Japan; 27 Department of Respiratory Medicine, Tokyo Women’s Medical University, Tokyo, Japan; 28 Department of General Medicine, Tokyo Women’s Medical University, Tokyo, Japan; 29 Clinical Research Center, Tokyo Medical and Dental University Hospital of Medicine, Tokyo, Japan; 30 Department of Medical Informatics, Tokyo Medical and Dental University Hospital of Medicine, Tokyo, Japan; 31 Respiratory Medicine, Tokyo Medical and Dental University, Tokyo, Japan; 32 Clinical Laboratory, Tokyo Medical and Dental University Hospital of Medicine, Tokyo, Japan; 33 Kawasaki Municipal Ida Hospital, Department of Internal Medicine, Kawasaki, Japan; 34 Department of Respiratory Medicine, Osaka Saiseikai Nakatsu Hospital, Osaka, Japan; 35 Department of Infection Control, Osaka Saiseikai Nakatsu Hospital, Osaka, Japan; 36 Department of Infectious Diseases, Tosei General Hospital, Seto, Japan; 37 Department of Respiratory, Allergic Diseases Internal Medicine, Tosei General Hospital, Seto, Japan; 38 Department of Emergency and Critical Care Medicine, Kansai Medical University General Medical Center, Moriguchi, Japan; 39 Japan Anti-Tuberculosis Association (JATA) Fukujuji Hospital, Kiyose, Japan; 40 Department of Pulmonary Medicine, Saitama City Hospital, Saitama, Japan; 41 Department of Infectious Diseases, Saitama City Hospital, Saitama, Japan; 42 Department of General Thoracic Surgery, Saitama City Hospital, Saitama, Japan; 43 Department of Pulmonary Medicine, Eiju General Hospital, Tokyo, Japan; 44 Division of Infection Control, Eiju General Hospital, Tokyo, Japan; 45 Department of Hematology, Eiju General Hospital, Tokyo, Japan; 46 Saiseikai Utsunomiya Hospital, Utsunomiya, Japan; 47 Department of Respiratory Medicine, Tohoku University Graduate School of Medicine, Sendai, Japan; 48 Department of Infectious Diseases, Tohoku University Graduate School of Medicine, Sendai, Japan; 49 Department of Respiratory Medicine, Kitasato University Kitasato Institute Hospital, Tokyo, Japan; 50 Division of Infection Control and Prevention, Osaka University Hospital, Suita, Japan; 51 Department of Biomedical Ethics and Public Policy, Osaka University Graduate School of Medicine, Suita, Japan; 52 Tachikawa Hospital, Tachikawa, Japan; 53 Department of Emergency and Critical Care Medicine, Tokyo Women’s Medical University Medical Center East, Tokyo, Japan; 54 Department of Medicine, Tokyo Women’s Medical University Medical Center East, Tokyo, Japan; 55 Department of Pediatrics, Tokyo Women’s Medical University Medical Center East, Tokyo, Japan; 56 Internal Medicine, Sano Kosei General Hospital, Sano, Japan; 57 Japan Community Health care Organization Kanazawa Hospital, Kanazawa, Japan; 58 Department of Respiratory Medicine, Saiseikai Yokohamashi Nanbu Hospital, Yokohama, Japan; 59 Department of Clinical Laboratory, Saiseikai Yokohamashi Nanbu Hospital, Yokohama, Japan; 60 Internal Medicine, Internal Medicine Center, Showa University Koto Toyosu Hospital, Tokyo, Japan; 61 Department of Respiratory Medicine, Japan Organization of Occupational Health and Safety, Kanto Rosai Hospital, Kawasaki, Japan; 62 Department of General Internal Medicine, Japan Organization of Occupational Health and Safety, Kanto Rosai Hospital, Kawasaki, Japan; 63 Ishikawa Prefectural Central Hospital, Kanazawa, Japan; 64 Kanagawa Cardiovascular and Respiratory Center, Yokohama, Japan; 65 Department of Respiratory Medicine, National Hospital Organization Tokyo Medical Center, Tokyo, Japan; 66 Department of Allergy, National Hospital Organization Tokyo Medical Center, Tokyo, Japan; 67 Department of General Internal Medicine and Infectious Diseases, National Hospital Organization Tokyo Medical Center, Tokyo, Japan; 68 Department of Respiratory Medicine, Toyohashi Municipal Hospital, Toyohashi, Japan; 69 Keiyu Hospital, Yokohama, Japan; 70 KKR Sapporo Medical Center, Department of respiratory medicine, Sapporo, Japan; 71 Division of General Internal Medicine, Department of Internal Medicine, St. Marianna University School of Medicine, Kawasaki, Japan; 72 Department of Emergency and Critical Care Medicine, St.Marianna University School of Medicine, Kawasaki, Japan; 73 Japanese Red Cross Medical Center, Tokyo, Japan; 74 Matsumoto City Hospital, Matsumoto, Japan; 75 Department of Emergency and Critical Care Medicine, Faculty of Medicine, Fukuoka University, Fukuoka, Japan; 76 Department of Infection Control, Fukuoka University Hospital, Fukuoka, Japan; 77 Department of Rheumatology, National Hospital Organization Hokkaido Medical Center, Sapporo, Japan; 78 Department of Respiratory Medicine, National Hospital Organization Hokkaido Medical Center, Sapporo, Japan; 79 Department of Emergency and Critical Care Medicine, National Hospital Organization Hokkaido Medical Center, Sapporo, Japan; 80 National Hospital Organization Kanazawa Medical Center, Kanazawa, Japan; 81 Nihon University School of Medicine, Department of Internal Medicine, Division of Respiratory Medicine, Tokyo, Japan; 82 Musashino Red Cross Hospital, Musashino, Japan; 83 Division of Respiratory Medicine, Social Welfare Organization Saiseikai Imperial Gift Foundation, Inc., Saiseikai Kumamoto Hospital, Kumamoto, Japan; 84 Department of Respiratory Medicine, Nagoya University Graduate School of Medicine, Nagoya, Japan; 85 Fukuoka Tokushukai Hospital, Department of Internal Medicine, Kasuga, Japan; 86 Fukuoka Tokushukai Hospital, Respiratory Medicine, Kasuga, Japan; 87 Department of Endocrinology, Hematology and Gerontology, Chiba University Graduate School of Medicine, Chiba, Japan; 88 Department of Emergency and Critical Care Medicine, Chiba University Graduate School of Medicine, Chiba, Japan; 89 National Hospital Organization Kumamoto Medical Center, Kumamoto, Japan; 90 Division of Infectious Diseases and Respiratory Medicine, Department of Internal Medicine, National Defense Medical College, Tokorozawa, Japan; 91 Sapporo City General Hospital, Sapporo, Japan; 92 Department of Internal Medicine, Tokyo Saiseikai Central Hospital, Tokyo, Japan; 93 Department of Pulmonary Medicine, Tokyo Saiseikai Central Hospital, Tokyo, Japan; 94 Department of Respiratory Medicine, Fujisawa City Hospital, Fujisawa, Japan; 95 Uji-Tokushukai Medical Center, Uji, Japan; 96 Department of Infectious Disease and Clinical Research Institute, National Hospital Organization Kyushu Medical Center, Fukuoka, Japan; 97 Department of Respirology, National Hospital Organization Kyushu Medical Center, Fukuoka, Japan; 98 Division of Respirology, Rheumatology, and Neurology, Department of Internal Medicine, Kurume University School of Medicine, Kurume, Japan; 99 Department of Infectious Disease, National Hospital Organization Kyushu Medical Center, Fukuoka, Japan; 100 Ome Municipal General Hospital, Ome, Japan; 101 Research Institute for Diseases of the Chest, Graduate School of Medical Sciences, Kyushu University, Fukuoka, Japan; 102 Department of Medicine and Biosystemic Science, Kyushu University Graduate School of Medical Sciences, Fukuoka, Japan; 103 Daini Osaka Police Hospital, Osaka, Japan; 104 Department of Emergency and Critical Care Medicine, Faculty of Medicine, University of Tsukuba, Tsukuba, Japan; 105 Department of Hematology, Faculty of Medicine, University of Tsukuba, Tsukuba, Japan; 106 Department of Nephrology, Faculty of Medicine, University of Tsukuba, Tsukuba, Japan; 107 Department of Cardiovascular Surgery, Faculty of Medicine, University of Tsukuba, Tsukuba, Japan; 108 Division of Pulmonary Medicine, Department of Medicine, Tokai University School of Medicine, Isehara, Japan; 109 Department of Anesthesiology and Intensive Care Medicine, Kyoto Prefectural University of Medicine, Kyoto, Japan; 110 Department of Infection Control and Laboratory Medicine, Kyoto Prefectural University of Medicine, Kyoto, Japan; 111 Department of Respiratory Internal Medicine, St Marianna University School of Medicine, Yokohama-City Seibu Hospital, Yokohama, Japan; 112 KINSHUKAI Hanwa The Second Hospital, Osaka, Japan; 113 Gifu University School of Medicine Graduate School of Medicine, Emergency and Disaster Medicine, Gifu, Japan; 114 Department of Respiratory Medicine, Tokyo Medical University Hospital, Tokyo, Japan; 115 JA Toride medical hospital, Toride, Japan; 116 Okayama Rosai Hospital, Okayama, Japan; 117 Himeji St. Mary’s Hospital, Himeji, Japan; 118 Emergency & Critical Care, Niigata University, Niigata, Japan; 119 Emergency & Critical Care Center, National Hospital Organization Kyoto Medical Center, Kyoto, Japan; 120 National Hospital Organization Tokyo Hospital Hospital, Kiyose, Japan; 121 Fujioka General Hospital, Fujioka, Japan; 122 Department of General Medicine, School of Medicine, International University of Health and Welfare Shioya Hospital, Ohtawara, Japan; 123 Department of Pharmacology, School of Pharmacy, International University of Health and Welfare Shioya Hospital, Ohtawara, Japan; 124 Department of Respiratory Medicine, International University of Health and Welfare Shioya Hospital, Ohtawara, Japan; 125 Department of Clinical Laboratory, International University of Health and Welfare Shioya Hospital, Ohtawara, Japan; 126 Department of Cardiology, Pulmonology, and Nephrology, Yamagata University Faculty of Medicine, Yamagata, Japan; 127 Division of Respiratory Medicine and Allergology, Department of Medicine, School of Medicine, Showa University, Tokyo, Japan; 128 Department of Pulmonary Medicine, Fukushima Medical University, Fukushima, Japan; 129 Kansai Electric Power Hospital, Osaka, Japan; 130 Division of Infectious Diseases, Kumamoto City Hospital, Kumamoto, Japan; 131 Department of Respiratory Medicine, Kumamoto City Hospital, Kumamoto, Japan; 132 Department of Emergency and Critical Care Medicine, Tokyo Metropolitan Police Hospital, Tokyo, Japan; 133 Department of Respiratory Medicine, Gunma University Graduate School of Medicine, Maebashi, Japan; 134 National hospital organization Saitama Hospital, Wako, Japan; 135 Tokyo Medical University Ibaraki Medical Center, Inashiki, Japan; 136 Department of Internal Medicine, Kiryu Kosei General Hospital, Kiryu, Japan; 137 Department of Pulmonary Medicine and Oncology, Graduate School of Medicine, Nippon Medical School, Tokyo, Japan; 138 Division of Respiratory Medicine, Tsukuba Kinen General Hospital, Tsukuba, Japan; 139 Division of Respiratory Medicine, Department of Internal Medicine, Toho University Ohashi Medical Center, Tokyo, Japan; 140 Division of Anesthesiology, Department of Surgery Related, Kobe University Graduate School of Medicine, Kobe, Japan; 141 Genome Medical Science Project (Toyama), National Center for Global Health and Medicine, Tokyo, Japan; 142 Department of Biomolecular Engineering, Graduate School of Tokyo Institute of Technology, Tokyo, Japan; 143 Laboratory of Veterinary Infectious Disease, School of Veterinary Medicine, Kitasato University, Aomori, Japan; 144 Laboratory of Viral Infection, Department of Infection Control and Immunology, Ōmura Satoshi Memorial Institute & Graduate School of Infection Control Sciences, Kitasato University, Tokyo, Japan; 145 Department of Insured Medical Care Management, Tokyo Medical and Dental University Hospital of Medicine, Tokyo, Japan; 146 Center for Infectious Disease Education and Research (CiDER), Osaka University, Suita, Japan; 147 Department of Organoid Medicine, Keio University School of Medicine, Tokyo, Japan; 148 Medical Innovation Promotion Center, Tokyo Medical and Dental University, Tokyo, Japan; 149 Department of Surgery, Keio University School of Medicine, Tokyo, Japan; 150 Institute of Research, Tokyo Medical and Dental University, Tokyo, Japan; 151 Institute for the Advanced Study of Human Biology (WPI-ASHBi), Kyoto University, Kyoto, Japan; 152 Department of Medicine, Center for Hematology and Regenerative Medicine, Karolinska Institute, Stockholm, Sweden; 153 AMED-CREST, Japan Agency for Medical Research and Development, Tokyo, Japan; 154 Laboratory for Systems Genetics, RIKEN Center for Integrative Medical Sciences, Yokohama, Japan; 155 Department of Genome Informatics, Graduate School of Medicine, the University of Tokyo, Tokyo, Japan

**Keywords:** Gene expression, Transcriptomics, Quantitative trait loci, Viral infection

## Abstract

Coronavirus disease 2019 (COVID-19) is a recently-emerged infectious disease that has caused millions of deaths, where comprehensive understanding of disease mechanisms is still unestablished. In particular, studies of gene expression dynamics and regulation landscape in COVID-19 infected individuals are limited. Here, we report on a thorough analysis of whole blood RNA-seq data from 465 genotyped samples from the Japan COVID-19 Task Force, including 359 severe and 106 non-severe COVID-19 cases. We discover 1169 putative causal expression quantitative trait loci (eQTLs) including 34 possible colocalizations with biobank fine-mapping results of hematopoietic traits in a Japanese population, 1549 putative causal splice QTLs (sQTLs; e.g. two independent sQTLs at *TOR1AIP1*), as well as biologically interpretable trans-eQTL examples (e.g., *REST* and *STING1*), all fine-mapped at single variant resolution. We perform differential gene expression analysis to elucidate 198 genes with increased expression in severe COVID-19 cases and enriched for innate immune-related functions. Finally, we evaluate the limited but non-zero effect of COVID-19 phenotype on eQTL discovery, and highlight the presence of COVID-19 severity-interaction eQTLs (ieQTLs; e.g., *CLEC4C* and *MYBL2*). Our study provides a comprehensive catalog of whole blood regulatory variants in Japanese, as well as a reference for transcriptional landscapes in response to COVID-19 infection.

## Abstract (web summary)

Genetic mechanisms influencing COVID-19 susceptibility are not well understood. Here, the authors analyzed whole blood RNA-seq data of 465 Japanese individuals with COVID-19, highlighting thousands of fine-mapped variants affecting expression and splicing of genes, as well as the presence of COVID-19 severity-interaction eQTLs.

## Introduction

RNA-sequencing (RNA-seq) is an important data source to understand disease biology^1^. Studies comparing the transcriptomic landscape of healthy and diseased samples have been widely performed to identify target genes and pathways for different diseases^2^. Also, RNA-seq data coupled with genotype data are powerful resources to understand the impact of genetic variation on gene expressions. Such studies of expression quantitative loci (eQTL) have been highly effective in deciphering the genetic basis of human traits^3,4^, by connecting genotype and phenotype through gene expression regulations. Recent development in statistical fine-mapping^5^ and colocalization^6^ methods have further provided principles to pinpoint the causal mechanisms at single variant resolution.

Coronavirus disease 2019 (COVID-19) is a recently-emerged infectious disease^7,8^, with symptoms including respiratory failures. More than millions of deaths to date are related to COVID-19^9,10^, and the world is seeking a deeper understanding of disease mechanisms for comprehensive therapeutic strategies. As part of such efforts^11,12^, a genome-wide association study (GWAS) meta-analyzing genomics data from COVID-19 cases and population controls has been performed^13^ to identify genomic loci associated with disease severity and susceptibility. At transcriptomics level, differential expression analyses have been performed to nominate large numbers of genes presenting expression dynamics upon COVID-19 infection^14,15^, motivating us for further investigation, replication and validation of these results. In particular, although studies focusing on eQTL effect of COVID-19 risk variants using external databases exist^16–18^, a comprehensive study of gene expression regulation landscape specifically in COVID-19 infected individuals is still limited.

**Fig. 1:**
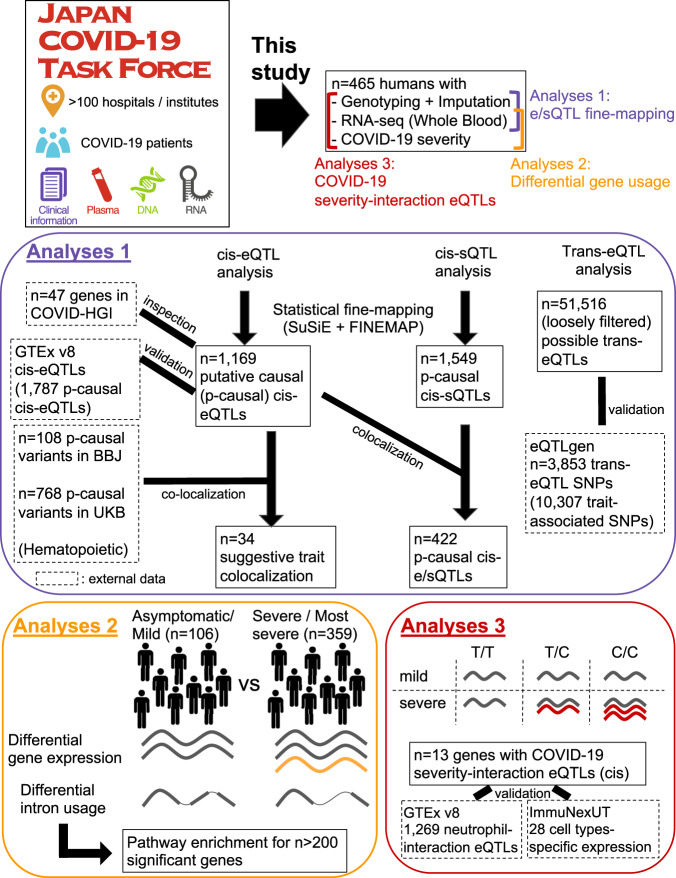
Overview of the study. Japan COVID-19 Task Force (JCTF) has collected DNA, RNA, and plasma from COVID-19 cases along with detailed clinical information. A subset of *n* = 500 (*n* = 465 after QC) harboring RNA-seq data was analyzed in this study. COVID-19 disease severity was used together with RNA-seq data to perform differential gene expression and intron usage analysis (red). Imputed genotyping data with RNA-seq data was used to perform cis-e/sQTL and trans-eQTL analysis, followed by fine-mapping (for cis-QTLs), colocalization and validation with external dataset (dotted line).

In this research, we provide a thorough analysis of whole blood RNA-seq data for 465 genotyped samples from the Japan COVID-19 Task Force^19^ (JCTF; Fig. 1), together with the results of cis-eQTL and cis-sQTL statistical fine-mapping, colocalization with biobank fine-mapping results and trans-eQTL search. We also utilize the different COVID-19 symptom severity information across samples to show the widespread effect of COVID-19 infection on the transcriptional landscape as well as its limited but non-zero effect on eQTL discovery, and characterize the set of eQTLs interacting with COVID-19 phenotype.

## Results

### Identifying cis-eQTLs from the JCTF RNA-seq data

**Fig. 2:**
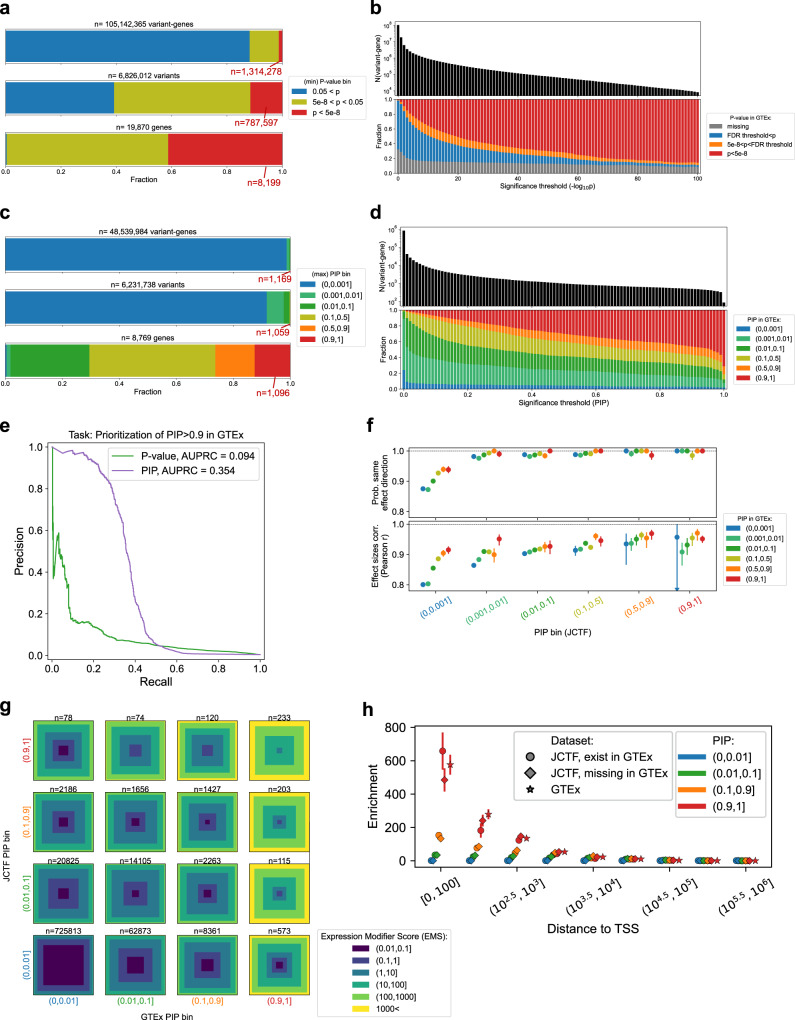
Overview of eQTL call and statistical fine-mapping from 465 samples in COVID-19 Task Force, and their comparison with publicly available eQTL data (GTEx). **a** Unique variant-gene pairs (top), variants (middle) and genes (bottom) classified into different marginal *p* value bins. The lowest p value was taken as a representative for variants and genes. **b** The number of variant-genes (y, top panel) classified into different marginal *p* value bins in GTEx v8 (y, bottom panel. 5e-8 = 5.0 × 10^−8^.), for different marginal *p* value thresholds (x < −log_10_(*p* value) for each x). **c** Unique variant-gene pairs (top), variants (middle) and genes (bottom) classified into different posterior inclusion probability (PIP) bins assigned by statistical fine-mapping of eGenes. The maximum PIP was taken as a representative for variants and genes. **d** The number of variant-genes (y, top panel) classified into different PIP bins in GTEx v8 (y, bottom panel), for different PIP thresholds (x < PIP for each x). **e** Precision-recall curve (PRC) for the task to classify variant-genes with 0.9 < PIP in GTEx and the ones with PIP < 0.001 from GTEx, using marginal p value (purple) or PIP (green). **f** Probability of presenting the same effect direction (first row), and the Pearson correlation of two (signed, marginal) effect sizes (second row) when comparing the effect sizes in JCTF and GTEx for different PIP bins. *x*-axis shows PIP bin in JCTF, and within an x-axis window, values are sorted along with PIP in GTEx. Error bar is the standard error of mean estimated via Fisher’s z-transformation, and the large error bar is due to having small data points (*n* = 4). **g** Distribution of a regulatory effect prediction score (Expression Modifier Score = EMS) bin for different PIP bins in our study (*y*-axis) and GTEx (*x*-axis). The fraction is represented as the area in each bin (the binning is coarser than in **f**). **h** Enrichment of variant-genes in specific range of distance to the transcription starting site (dTSS) for each PIP bin (color) and in each dataset condition compared to random. EMS is not available for variants missing in GTEx, and dTSS is of the best individual features predictive for putative causal eQTLs in the absence of EMS^24^.

We performed an eQTL call for 105,142,365 cis variant-gene pairs (v-g) in 465 samples that passed quality control (QC; Fig. S1; “Methods”) step. 1,314,278 v-gs (1.24%) had *p* value lower than genome-wide threshold (< 5.0 × 10^−8^; corresponding to 11.5% = 787,597 of 6,826,012 variants or 41.3% = 8199 of 19,870 genes; Fig. 2a**;** Tables S1, S2). The result was nearly perfectly consistent regardless of whether or not including COVID-19 severity status as a covariate, presumably because the effect is largely captured by PEER factors^20^ (Only six variant-genes were off by more than 2 in a binned −log_10_(*p*) scale; Figs. S2, S3, Supplementary Data 1). When compared with whole blood eQTL data in GTEx^4^, 32.4% (34,059,915 out of 105,142,365) of v-gs were missing, reflecting the different genetic background between two populations (Japanese versus mainly European) (Fig. 2b leftmost bar, Fig. S4). The proportion of v-gs with *p* value lower than 5.0 × 10^−8^ in GTEx consistently increased along with p value threshold in our dataset. For example, when filtering to variant-genes with *p* value lower than 10^−100^, 85.5% (6908 out of 8076) of the variants (or 93.5% of the non-missing variants) showed *p* value < 5.0 × 10^−8^ in GTEx (Fig. 2b rightmost bar), validating our association statistics.

### Statistical fine-mapping of cis-eQTLs

We then performed statistical fine-mapping using two methods^21,22^ for 8199 genes that harbored at least one variant with genome-wide significance *p* < 5.0 × 10^−8^ (= “eGenes”), as well as additional 570 genes that harbored rare significant variants, and identified 1169 putative causal v-gs (i.e. p-causal eQTLs, defined as v-gs with posterior inclusion probability = PIP > 0.9 across two methods^21,22^; Fig. 2c, Fig. S5, containing 1059 unique variants and 1096 unique genes; Table S1). We used a uniform threshold of *p* < 5.0 × 10^−8^ to define eGenes for consistency with previous fine-mapping literatures^23,24^, but alternative possible choices such as *q* value based per-gene false discovery rate (FDR) are equally possible. For example, using FDR < 0.05 threshold resulted in 13,898 genes; See Fig. S6 and Supplementary Note. To test the validity of our fine-mapping results, we compared the PIPs from two study populations (JCTF versus GTEx; Fig. 2d, S7a–c). The fraction of variant-genes identified as p-causal eQTLs in GTEx consistently increased along with the PIP threshold in our study (JCTF). Moreover, among 505 p-causal eQTLs where PIPs were also calculated in GTEx, nearly half (46.1%, *n* = 233) were also identified as p-causal eQTLs in GTEx, and for the most confident set (i.e. PIP = 1 in our study; *n* = 90 with non-missing GTEx PIP) (Fig. 2d) of v-gs, the fraction of p-causal eQTLs in GTEx reached 71.1% (64 out of 90). Out of the remaining 26 variant-genes, 21 were explained by one or more of (1) moderately high PIP (>0.5) (*n* = 9), (2) harboring top PIP in the gene, even though it does not reach the PIP > 0.9 threshold (*n* = 12), (3) non-negligible PIP (>0.1) only in SuSiE (*n* = 20) in GTEx, or (4) >10-fold differences in the minor allele frequencies between JCTF and GTEx (*n* = 16), suggesting that inconsistency mainly reflected the differences in allele frequencies and LD structures, as well as the uncertainty of the fine-mapping algorithms (Figs. S4, S7d, e). We also evaluated the performance of prioritizing p-causal eQTLs identified in GTEx using two measures (*p* value or PIP in JCTF), and showed that PIP achieves higher area under precision-recall curve (AUPRC) (0.354 vs 0.094, Fig. 2e). These results demonstrate the robustness of our fine-mapping results, as well as largely shared causal regulatory architecture between two study populations at single variant resolution.

We also investigated the marginal effect sizes (*β*) in two datasets, for the v-gs passing FDR threshold (<0.05) in GTEx v8, and confirmed the high effect size correlation and the effect direction concordance (*r* = 0.74 and 83%, *p* < 10^−100^). The concordance was underscored when shifting to higher PIP bins, for both our dataset and GTEx (100% direction concordance when PIP > 0.9 in both; Fig. 2f). In addition to serving as another evidence for largely shared causal effect in two populations, these observations suggest that PIP in JCTF improves our ability to prioritize regulatory v-gs, even after given the PIPs from GTEx (or vice versa). We further confirmed that by comparing a regulatory effect prediction score (Expression Modifier Score = EMS^24^) distribution in JCTF and GTEx (Figs. 2g, S8a, b) -- The proportion of variant-genes with low (high) EMS nearly consistently decreases (increases) along with the PIP in JCTF, across different PIP bins in GTEx (*p* value < 6.2 × 10^−7^ in Fisher’s exact test for proportion of variant-gene with EMS > 1).

32.4% (34,059,915 out of 105,142,365) of the v-gs in JCTF, including 396 p-causal eQTLs, are missing in GTEx. To validate the quality of such p-causal eQTLs in JCTF-unique variants, we compared the distance to transcription starting site (dTSS) distribution stratified by the different PIPs (PIP in JCTF, for variant missing vs existing in GTEx, and PIP in GTEx; Fig. 2h). TSS-proximal variants were enriched for p-causal eQTLs similarly in all three categories (*p* > 0.05 in Fisher’s exact test for difference in the fraction of PIP > 0.9 in the top bin), suggesting that the PIPs of JCTF-unique variants are equally calibrated as those of variants existing in GTEx (either from fine-mapping in JCTF or fine-mapping in GTEx). We also compared the fraction of reporter assay QTLs (raQTLs)^25^; again confirming the similarity in the enrichment pattern between categories (Fisher’s exact test *p* > 0.05 for the top PIP bin in two cell types; Fig. S8c, d, “Methods”).

These results show that discovering eQTLs from RNA-seq data of different genetic background and refining the eQTL signals via statistical fine-mapping is important for identification of p-causal eQTLs, for (1) it improves the ability to prioritize regulatory eQTLs compared to fine-mapping in a single population, when a variant exists in both populations (i.e. improving the specificity in regulatory variant discovery), and (2) it allows discovery of novel p-causal eQTLs with the same level of calibration in PIP estimate for population-unique variants (i.e. improving the sensitivity). Regarding (1), we also investigated eQTL data from African American individuals in Multi-Ethnic Study of Atherosclerosis study^26^ (MESA, *n* = 233), and discovered that utilizing PIPs from both JCTF and GTEx in combination increases the ability to prioritize likely-regulatory eQTLs in MESA (Fig. S9), highlighting the value of integrating information from even larger (>2) numbers of cohorts with diverse backgrounds.

### splice QTL (sQTL) fine-mapping and colocalization

**Fig. 3:**
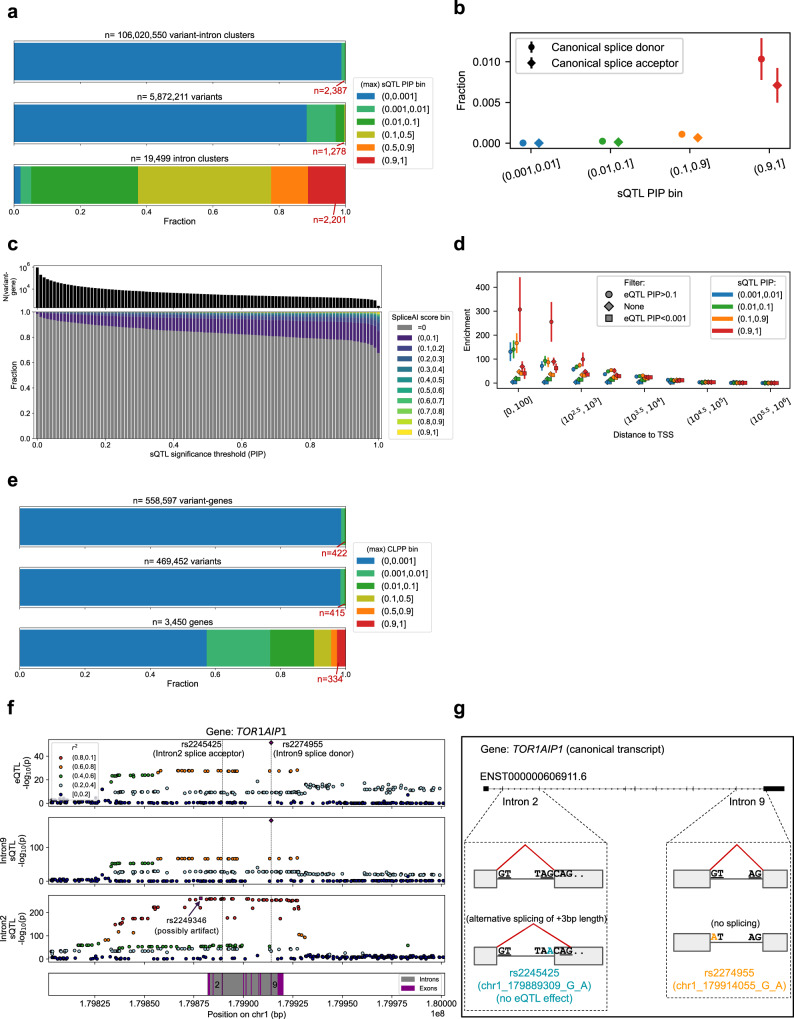
Overview of sQTL call and statistical fine-mapping from 465 samples in COVID-19 Task Force. **a** Unique variant-intron pairs (top), variants (middle) and introns (bottom) classified into different PIP bins. The highest PIP was taken as a representative for variants and introns. **b** Binned distribution of the distance to transcription starting site (TSS) for sQTLs in different PIP bins. **c** The fraction (top) and the number (bottom) of variant-introns classified as splice regions (left), donors (center) or acceptors (right) variants, for different sQTL PIP bins. **d** Unique variant-gene pairs (top), variants (middle) and genes (bottom) classified into different bins of colocalization posterior probability (CLPP) with eQTL PIPs in the same study. The highest CLPP was taken as a representative for variants and genes. **e** Binned distribution of the distance to transcription starting site (TSS) for sQTLs in different PIP bins, for the ones with (top) and without (bottom) suggestive eQTL colocalization. **f** Locus zoom for eQTL and sQTL effect on *TOR1AIP1* gene. rs2274955 (dotted line in the right) is on the canonical splice donor site of intron 9 (9th gray square from the left), whereas rs2249346 (dotted line in the left) is upstream of the transcription start site (TSS) of the gene. **g** Detailed description of the splice pattern differences. In **d**–**f**, maximum PIP was taken for introns in a single gene to derive a PIP for each variant-gene.

We next performed sQTL call followed by statistical fine-mapping with the same pipeline (e.g. filtering to *p* < 5.0 × 10^−8^ before applying fine-mapping algorithms). We identified 2,387 p-causal variant-introns^27^ in 106,020,550 variant-intron pairs (Fig. 3a). p-causal sQTLs (v-gs with sQTL PIP > 0.9 for at least one intron in the corresponding gene region, *n* = 1549) were enriched for known canonical splice donor or acceptor sites (Fig. 3b). On the other hand, a large majority of p-causal sQTLs (98.3%) were not annotated as splice sites, and a substantially higher but still a minority fraction of p-causal sQTLs presented non-zero scores in a deep-neural network based prediction^28^ (SpliceAI; 22.0% at PIP > 0.9; Fig. 3c), suggesting a wide range of splice effects that are not limited to canonical sites^29^ (and/or slight miscalibration of PIPs).

Alternative splicing can also result in a difference in the overall mRNA expression level (e.g. via nonsense mediated decay^30^). When we quantified the distribution of dTSS stratified by both sQTL PIP and eQTL PIP (Fig. 3d, circle and square shape dots), not only the v-gs with high eQTL PIP (eQTL PIP > 0.1, *n* = 2882 with sQTL PIP > 0.001), but also the v-gs that are unlikely to be causal eQTLs (eQTL PIP < 0.001, *n* = 484,977 with sQTL PIP > 0.001) were heavily concentrated towards TSS-proximal regions (40.3x enrichment for the top dTSS < 100 bin compared to random; *p* = 2.2 × 10^−5^ in Fisher’s exact test comparing the top and bottom PIP bin), suggesting the presence of both shared and distinct mechanisms of eQTL and sQTL effects. We then performed a colocalization analysis between sQTLs and eQTLs. Of 916,223 variant-gene pairs with sQTL PIP > 0.001, 422 were identified as possibly colocalizing v-gs (colocalization posterior probability^31^ = CLPP > 0.1; Fig. 3e). For example, we nominated rs2274955 (chr1_179914055_G_A) as a p-causal s/eQTL for *TOR1AIP1*, a gene well studied for alternative splicing patterns^32,33^ (Fig. 3f, g; Fig. S10). rs2274955 is a canonical splice donor of the intron 9. Of note, there is a clear sQTL signal independent from that of rs2274955 including 49 tightly linked (*r*^2^ > 0.8) variants with *p* value < 10^−200^. While two fine-mapping algorithms nominated an upstream variant rs2249346 (chr1_179878500_C_T) as the putative causal sQTL for intron 2, our manual inspection suggests rs2245425 (chr1_179889309_G_A) as the causal variant, disrupting the canonical splice acceptor site of intron 2 and introducing subtle intron length difference of 3 bp (and therefore has minimal effect to the overall gene expression level; Fig. 3g left). This example of *TOR1AIP1* highlights independent sQTL signals where one of them results in an order of magnitude stronger eQTL signal, as well as the limitation of fine-mapping algorithms with uniform prior.

Our sQTL fine-mapping overall highlighted a wide variety of putative causal sQTL effects that exist within or outside of the context of canonical splice sites or causal eQTL effects, motivating us for further comprehensive characterization of splice pattern variations.

### eQTL colocalization with putative complex-trait-causal variants from biobank studies

**Fig. 4:**
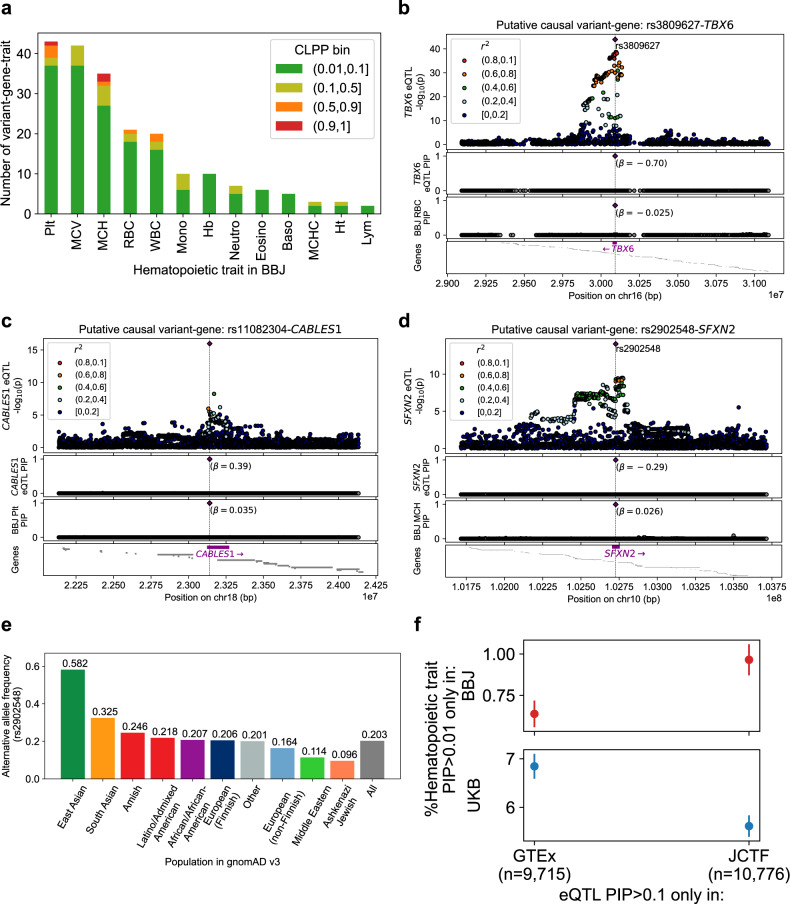
Colocalization of eQTLs with possible hematopoietic trait-causal variants suggested in biobank studies. **a** Number of variant-gene-trait pairs (y axis) with suggestive colocalization posterior probabilities (0.01<CLPP), for different hematopoietic traits (x axis) in Biobank Japan (BBJ). **b**–**d** Association *p* value (top), eQTL PIP (second row) and BBJ trait PIP (third row) of the SNVs in ±1 Mb (**b**, **c**) or ±100 kb (**d**) window, as well as the location of the genes (bottom row). The putative causal variants and genes are colored with purple. **e** The alternative allele frequency of rs2902548 in gnomAD. Additional descriptions about these variant-genes are available in Supplementary Note. **f** Percentage of variant (y axis) with suggestive hematopoietic trait-causal signal (0.01<PIP) only in Biobank Japan (top), or only in UK Biobank (bottom), for variants with possible putative causal eQTL effects (PIP > 0.1) unique to GTEx (left), or our dataset (right).

To investigate the phenotypic relevance of p-causal eQTLs identified in our study, we compared our statistical fine-mapping results with that of a large-scale biobank study from the same geographical region (the Biobank Japan Project; BBJ^23,34^, *n* = 178,726). Focusing on 13 hematopoietic traits (i.e., red blood cell, while blood cell, and platelet-related traits), we identified 34 possibly colocalizing variant-gene-trait pairs (CLPP > 0.1; Fig. 4a, Fig. S11), including 6 with high confidence (CLPP > 0.75; Supplementary Data 2).

We highlight three examples in Fig. 4b–e and Supplementary Note. In particular, rs2902548 (chr10_102727625_C_T), an intronic SNP on the Sideroflexin 2 (*SFXN2*) gene, was putatively causal for both decreased *SFXN2* expression in JCTF and increased mean corpuscular hemoglobin (MCH) in BBJ^34^ (*β* = −0.29 and 0.026 for the T allele, PIP > 0.99 in both, resulting in CLPP > 0.99; Fig. 4d). A study^35^ reported that the gene is involved in mitochondrial iron homeostasis and showed that knocking out the *SFXN2* gene results in an increase of mitochondrial iron level in cultured human cells, suggesting that rs2902548 increases MCH through down-regulation of *SFXN2* gene. The alternative allele (T) of rs2902548 is the major allele only in EAS in gnomAD^36^ (Fig. 4e), and that the variant shows eQTL PIP > 0.9 in GTEx^24^, but the effect on MCH does not reach genome-wide significance in UK biobank (UKB^37^), possibly because of low effect size and smaller allele frequency.

We next investigated the colocalization landscape in a cohort from a different geographical region. Specifically, we compared the PIP across two by two patterns of specific enrichment in Japanese or European cohorts (JCTF or GTEx, by BBJ or UKB; Supplementary Data 2). The proportion of variants presenting PIP > 0.01 specifically in UKB was higher for the variants presenting PIP > 0.1 specifically in GTEx (*p* = 0.01 in Fisher’s exact test; Fig. 4f), suggesting the increase in the power of colocalization analysis by matching the population.

### An attempt to colocalize host genetic factors of COVID-19

We also sought to identify regulatory variants on a set of 47 genes suggested as relevant with COVID-19 severity in the GWAS conducted by COVID-19 Host Genetics Initiative (HGI, release 5)^13^ based on proximity with the lead variant. Although we identified 11 variants that are potentially regulatory to total 9 genes through gene expression regulation (PIP > 0.5) (Supplementary Data 3, Fig. S12), we note that our results do not nominate phenotype-causal variants or genes with high confidence (Supplementary Note). Further omics studies on COVID-19 infected population with a diverse population are warranted.

### Biological insights from trans-eQTL analysis

**Fig. 5:**
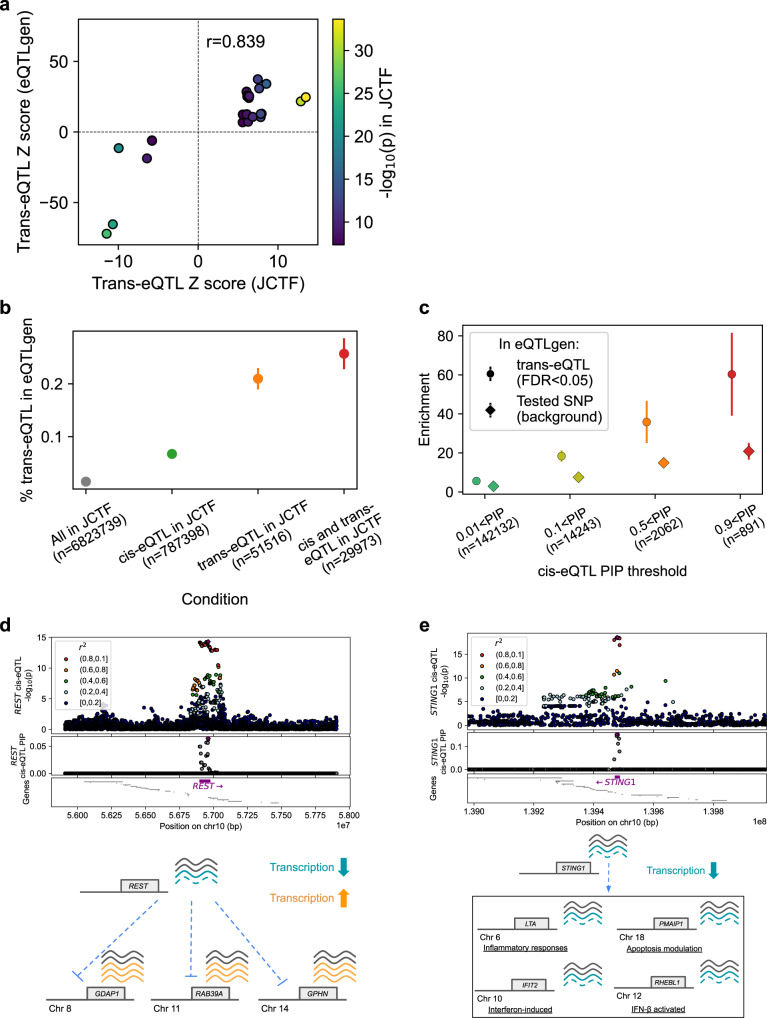
Insights from trans-eQTL analysis. **a** Scatter plot showing the trans-eQTL effect sizes (z-score) in our analysis (x-axis) and in eQTLgen (*y*-axis) for the 37 variant-genes identified as trans-eQTL both in two analyses. The color represents the nominal p value in our analysis. **b** Percentage of variants presenting trans-eQTL effect in eQTLgen (FDR < 0.05), for variants in our dataset with different conditions (*x*-axis). **c** Enrichment of variants presenting trans-eQTL effect in eQTLgen (circle) or assessed in eQTLgen (diamond) relative to all the variants in our dataset, for variants with different maximum cis-eQTL PIP (*x*-axis). **d**, **e** Association *p* value (top), cis-eQTL PIP (second row) and the location of the genes (third row) for ±1 Mb (**d**) or 0.5 Mb (**e**) of the variant with possible trans-eQTL effects mediated by cis-eQTL effects, with schematic overview of the trans-eQTL mechanisms. Blue dotted line represents the decrease of the effect (arrow; positive, non-arrow; negative).

We performed trans-eQTL mapping to nominate 51,516 possible trans-eQTL variants (passing a loose *p* value threshold of 5.0 × 10^−8^; “Methods”). We used a recently published large trans-eQTL resource from *n* = 31,684 predominantly from European samples (eQTLgen^38^) to evaluate our findings. We observed consistent effect sizes for all 37 trans-eQTLs that are also annotated as trans-eQTLs in eQTLgen (pearson *r* = 0.839 in the unit of z-score; Figs. 5a, S13), and the proportion of variants presenting trans-eQTL effect in eQTLgen were significantly higher for variants with possible trans-eQTLs effects in our dataset (orange dot in Fig. 5b). Being a cis-eQTL in our dataset further increased the chance of being a trans-eQTLs in eQTLgen (green and red dots in Fig. 5b). We presume this observation, suggesting trans-eQTL effects mediated by cis-eQTL effects as one of the major mechanisms^38,39^, is not simply due to ascertainment in eQTLgen or tagging of non-causal cis-eQTLs, since the enrichment was higher than the background when stratified by PIP (Fig. 5c; Fisher’s exact test *p* = 0.01 for the top PIP bin; Supplementary Note).

Although individual examples require cautious interpretation and confirmation with larger sample sizes, we highlight two biologically suggestive findings. First, we replicated the trans-eQTL association presumably mediated by cis-eQTL effect on the *REST*^38,40^ gene (Fig. 5d), for three biologically relevant genes at *p* < 5.0 × 10^-8^ threshold (*GDAP1*, *RAB39A*, and *GPHN*, while the other 85 trans-genes nominated in eQTLgen did not reach significance, presumably reflecting the sample size differences). Second, the lead cis-eQTL (rs78233829) for Stimulator Of Interferon Response CGAMP Interactor 1 (*STING1*) gene expression (*β* = −0.311) showed negative trans-eQTL effect for four genes (*LTA*, *IFIT2*, *RHEBL1* and *PMAIP1*. *β* = −0.246, −0.259, −0.39, and −0.327 respectively) passing the bonferroni-corrected threshold of *p* < 3.6 × 10^−13^. These four genes are all related with interferon activity^41–45^ (e.g. *IFIT2* is a well-known interferon-induced gene), suggesting the trans-eQTL effect mediated by the IFN pathway (Fig. 5e). We note that previous GWAS^19^ did not suggest an association of the variant against COVID-19 infection (*p* > 0.05), warning us that eQTL effects on immune-related genes do not necessarily result in currently detectable effects on COVID-19 susceptibility.

### The effect of severe COVID-19 phenotype on transcriptional landscape

**Fig. 6:**
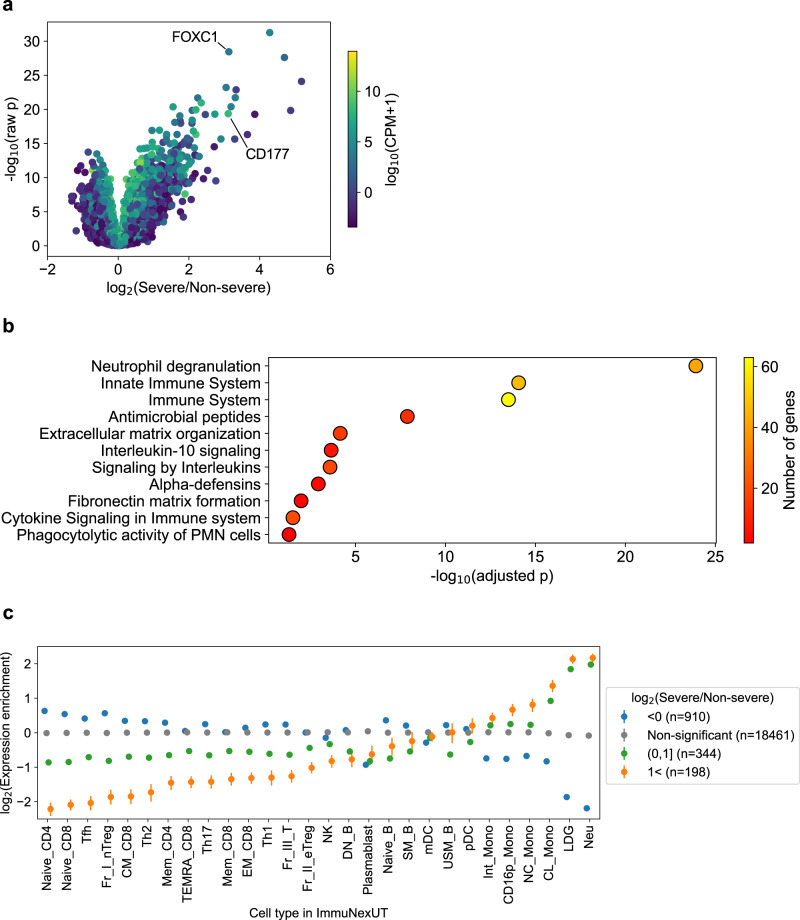
Transcriptional interpretation of COVID-19 susceptibility. **a** Volcano plot showing the difference of the RNA expression level between severe and non-severe COVID-19 cases (*x* axis, log_2_(severe/non-severe)), and the statistical significance (likelihood ratio test *p* value, *y* axis). Color shows the log_10_(count per million + 1). **b** GO term enrichment of top-enriched genes in severe cases (*n* = 198), including genes such as *CD177* (Human Neutrophil Alloantigen 2a), or *FOXC1* as reported in refs. 46,47. **c** Cell-type-specific enrichment of the gene sets with different levels of differential expression, for 28 cell types from ImmuNexUT.

Our cohort is unique in that the samples were ascertained for COVID-19 infection with ranging severity. To understand the influence of the severe COVID-19 phenotype on the transcriptional landscape, we divided the samples into two groups (severe vs non-severe phenotype; *n* = 359 and 106, respectively) and performed differential gene expression analysis (Fig. 6a). We observed larger number of genes with increased expression in severe cases-group (198 significantly increasing genes vs 10 decreasing genes at Bonferroni threshold and >2 fold difference, fisher exact test *p* = 1.1 × 10^−46^; Supplementary Data 4, 5). The genes with increasing expression in severe cases-group are enriched for innate immune system annotations such as neutrophil degranulation (Fig. 6b), consistent with previous reports^15,46,47^. To understand the cell type specificity of such differentially expressed genes, we calculated the expression enrichment for 28 immune cell types in ImmuNexUT^48^, stratified by the differential expression status of the genes (Fig. 6c, Figs. S14, S15; “Methods”). Neutrophils (Neu), Low-density granulocytes (LDGs) and monocytes consistently showed enrichment in expression-increasing genes, while naive CD4 and CD8 were depleted, again highlighting innate immune system activation in severe COVID-19 phenotype^49,50^. We note that such changes are often observed as a general response to infection^51,52^.

Differential expression results in whole blood are known to be sensitive to cell type composition changes^53^. When we applied cell type decomposition on our bulk expression data using CIBERSORT^54^ and included major inferred cell type composition as covariates, fewer genes reached statistical significance, although the enrichment patterns remained roughly consistent (Fig. S16, Supplementary Data 6, 7). We thus note that part of the observed gene expression differences is due to changes in the fraction of cell types rather than an increased expression within a cell type, in agreement with ref. 15.

We also performed differential splicing analysis to identify differences in intron usage^25^ in response to severe COVID-19 phenotypes. One hundred and ninety introns corresponding to 73 genes were identified to have different usage (Bonferroni adjusted *p* < 0.05, absolute fold change > 2; Supplementary Data 8, 9), with mild enrichment in genes with immune system-related functions such as *CD82* and *SERPINB2* (Fig. S17). We did not observe evidence of differential splicing for *OAS1*^12^ and *ACE2*^11^ (two major genes known for links between their splicing pattern and COVID-19 disease phenotype; Fig. S17h, i).

### eQTL effects are relatively stable in severe COVID-19 phenotype

**Fig. 7:**
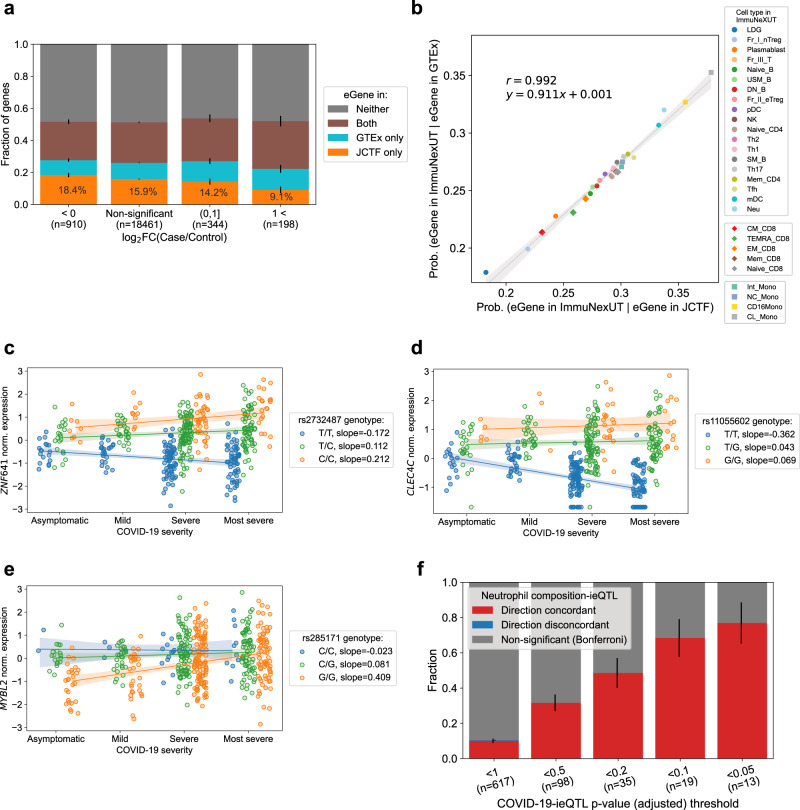
The effect of COVID-19 phenotype on transcriptional regulation landscape. **a** The fraction of genes that are identified as eGenes only in our analysis (orange) or in GTEx (cyan) (*y*-axis), both (brown) or neither (gray), for a set of genes with different levels of differential expression (*x*-axis). **b** Scatter plot presenting the proportion of eGenes (*p* < 5.0 × 10^−8^) identified either in whole blood RNA-seq in our study (*x*-axis) or GTEx (*y*-axis), that are replicated in each of the 28 cell types from ImmuNexUT. **c**–**e** Examples of COVID-19-interaction eQTLs (ieQTLs). *y* axis is the normalized expression, and the position of each dot is shifted randomly along *x*-axis direction for visualization purposes. **f** The fraction of COVID-19-ieQTLs replicated as estimated neutrophil count-ieQTLs, as a function of significance. Error bar in **a** and **f** are the standard error of the mean of the bottom bar. Error band in **b** to **e** denotes the 95% confidence interval.

We next sought to evaluate the effect of COVID-19 infection on the eQTL call. We compared the fraction of eGenes (genes with at least one variant with eQTL *p* value < 5.0 × 10^−8^) unique to our study and to GTEx, stratified by the differential expression status of the genes. We observed a decrease in the fraction of eGenes unique to JCTF, for genes highly expressed in severe COVID-19 cases (Fig. 7a, 0.57× for top bin and chi square contingency test *p* = 0.008). To further understand the biology of eGenes uniquely discovered in different cohorts, we compared the replication rate of eGenes in different immune cell types in ImmuNexUT^48^ (Fig. 7b). There was a very strong correlation (*r* > 0.99) between the replication rate in two cohorts (JCTF and GTEx), suggesting overall biological consistency between eGenes in our datasets and in GTEx. On the other hand, neutrophils (Neu) and LDGs particularly showed low replication rate in JCTF relative to GTEx. These results combined with the pathway and expression enrichment quantified in the previous section together indicate that severe COVID-19 phenotypes might slightly change the transcriptional regulation landscape and decrease our power to identify eQTLs, especially for neutrophils presumably due to increased mean and variance in the gene expression in response to viral infection that is near-independent of the genotypes in the cis-regions (Supplementary Note).

Nevertheless, as examined in Fig. S18 (concordance in the baseline expression level) and Fig. S1 (concordance in the eQTL signal), we assume the overall ascertainment bias is limited, allowing us to replicate the majority of the GTEx results. Together with our GWAS study^19^, our observation agrees with^55^ that the majority of the transcriptional differences observed are the consequence of infection rather than genetic variations.

### Characterization of eQTL effects interacting with severe COVID-19 phenotype

We then hypothesized that COVID-19 infection allows us to capture a set of interaction eQTLs (ieQTLs) that presents eQTL effects of different magnitude for different conditions (e.g. larger effect in mild phenotype), and performed ieQTL analysis (“Methods”). 13 ieGenes (genes with minimum *p* value for the interaction term < FDR = 0.05 threshold, including 10 ieGenes with *p* < 5.0 × 10^−8^) were discovered (Supplementary Data 8). As examples, *ZNF641* is subject to different levels of regulatory effect for each COVID-19 phenotype bin (Fig. 7c). *CLEC4C*, known for its role in antiviral immune response and cold^56,57^, shows decreased expression in severe cases, only when the T/T alleles are observed at rs11055602 (Fig. 7d). The variant is nominally associated with infectious phenotype in Finngen^58,59^ (*p* = 1.8 × 10^−5^). Although *CLEC4C* is also almost exclusively expressed in plasmacytoid dendritic cells^48^ (pDCs), the same effects for these two genes are replicated in GTEx as ieQTL for neutrophil score (Figs. S19, S20).

To further characterize such ieQTLs in the context of neutrophil degranulation, we examined the proportion of genes identified as ieQTLs interacting with an inferred neutrophil score in GTEx^4,60^ (Fig. S19c). While the proportion of such neutrophil-ieGenes increased along with the significance threshold in our ieQTL analysis, it did not exceed 60% at the most stringent threshold (adjusted *p* < 0.05). For example, the eQTL effect of rs285171 on *MYBL2* gene diminished in samples with severe and most severe COVID-19 symptoms (Fig. 7e), where such interaction was not replicated in neutrophil ieQTL analysis in GTEx (of note, *MYBL2* gene is known to be involved in stress responses^61,62^ and is only lowly expressed in neutrophils; Fig. S20).

**Fig. 8:**
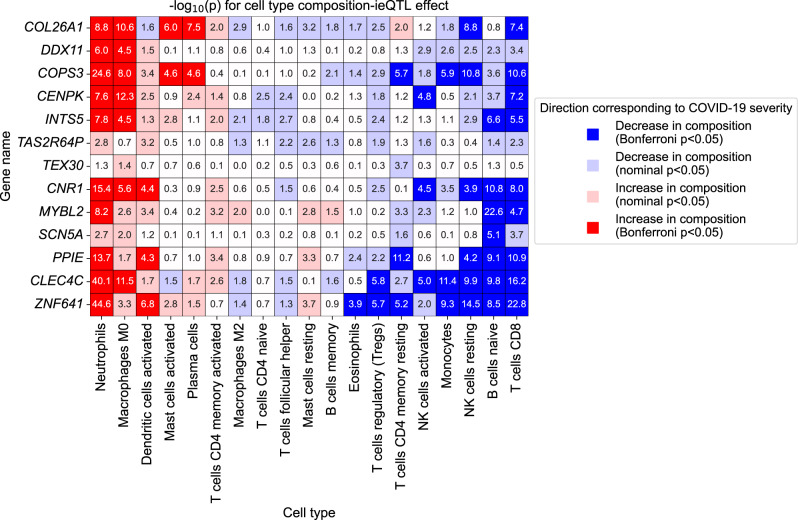
COVID-19 severity-interaction eQTLs interacts with composition of various immune cell types. For each of the 13 genes with COVID-19 severity-interaction eGenes (FDR < 0.05) (= row), significance for interaction eQTL effect with inferred cell type compositions (= columns) are plotted in −log_10_(*p*) scale. Colors show the significance as well as the direction of the ieQTL effect relative to the COVID-19 severity (red means severe COVID-19 case corresponds to larger cell type composition in terms of interaction effect, and blue is the other way. Bonferroni *p* = 1.7 × 10^−4^ = 0.05/13 genes/22 cell types). Row and columns are sorted based on the number of positive and negative significant results, where three cell types with no significant results are removed.

Next, we tested ieQTL effects for each of the inferred cell type composition from CIBERSORT^54^. This not only replicated the neutrophil ieQTLs (Fig. 7f; *MYBL2* also reaching significance) but also highlighted ieQTL effects with wide range of cell types, such as interaction with increased M0 macrophage, decreased naive B cells and CD8+ T cells compositions (Fig. 8).

We also performed fine-mapping of the eQTL and ieQTL signals separately for 13 ieGenes and observed that the signals are mostly shared (Figs. S21, 22, Supplementary Note). Finally, we note that these ieQTLs are not among the GWAS significant variants in refs. 13, 19.

In summary, our results suggest that the interaction between the genotype and COVID-19 phenotype status is characterized by dynamics of cell type composition such as an increase in neutrophils in COVID-19 patients along with the severity of the disease, and motivates us for further characterization of the interaction between COVID-19 phenotype and gene expression regulation.

## Discussion

In this work, we performed a set of analyses ranging from cis-e/sQTL fine-mapping, colocalization, trans-eQTL, cis-ieQTL analysis to differential expression using a dataset of whole blood RNA-seq data from 465 genotyped samples with severe to asymptomatic COVID-19 patients in Japan from JCTF. Comparing our fine-mapping results with a different cohort (GTEx) showed that statistical fine-mapping results in one cohort adds information on top of the other, confirming that the previous observation in biobank complex-trait fine-mapping^23^ holds true in eQTL fine-mapping as well. Colocalization analysis with biobank fine-mapping results highlighted putative causal association between variants and hematopoietic traits through cis-gene regulation in whole blood, including those enriched for Japanese populations. sQTL fine-mapping suggested the presence of both shared and distinct mechanisms of eQTL and sQTL effects. Trans-eQTLs analysis suggested mediation by cis-eQTL as one of its major mechanisms. Finally, we evaluated the impact of COVID-19 phenotype on transcriptional landscape to reveal a widespread increase of immune response related genes’ expression, characterized the expression change in terms of tissue specificity, highlighted ieQTLs that show distinct regulatory pattern and its possible role in COVID-19 phenotype.

Altogether, our study is unique and valuable not only because it serves as one of the largest reference databases^4,48,63,64^ of gene expression regulation at statistically fine-mapped, single variant resolution in a Japanese population, but also because it characterizes gene expression regulation landscape specifically in COVID-19 infected samples.

Our study also harbors potential limitations. First, when using our data as a reference, the effect of COVID-19 infection on our statistical fine-mapping is limited (e.g. Figs. S2, S18 and Fig. 6c) but non-zero. Second, the sample size is still not likely to reach saturation. Increased sample sizes and diversity^65^ should allow discovery of a larger number of disease-relevant transcriptional dynamics with statistical confidence, including ones with small effect sizes. Third, although our analysis utilizing external per-cell type eQTLs strongly suggest activation of specific blood cell types such as neutrophil or pDCs, confidently distinguishing gene expression dynamics universal to blood cells versus those due to cell type composition changes remains challenging from our dataset. Lastly, we applied genotyping followed by imputation instead of direct whole-genome sequencing (WGS), thereby not fully assessing regulatory impact of rare variants where imputation quality is likely to drop.

These points at the same time motivate us for future work that utilizes WGS, with larger sample size, and ideally RNA-sequencing at single cell resolution. Methodological developments are also of prominent importance; for example, our analysis of independent sQTL signals on the same gene (Fig. 3f) highlights the opportunity to include functional annotations^24,66^ tailored for sQTL fine-mapping. In addition, although we focused on hematopoietic traits, further utilizing biobank scale studies with expanding numbers of variants and phenotypes^67^ (e.g., other respiratory, immunological, or infectious traits) would be valuable for novel colocalizing variant identifications.

To date, our result serves as one of the most comprehensive studies focused on statistical fine-mapping of regulatory variants in a Japanese population, as well as a reference for transcriptional landscapes in response to COVID-19 infection. Our study demonstrates the value of transcriptomics study with large sample sizes to decipher disease mechanisms, and motivates us for further characterization of the shared and distinct regulatory landscape of the genome between different populations, in healthy and disease state.

## Methods

### Ethics

We have complied with all relevant ethical regulations. This study was approved by the ethical committees of Keio University School of Medicine, Osaka University Graduate School of Medicine, and affiliated institutes. Informed consent was obtained from all participants.

### The COVID-19 Task Force data

The study participants were recruited through Japan COVID-19 Task Force, which is described in detail in ref. 19. Briefly, the study samples included 2520 COVID-19 cases and 3341 controls genotyped using Infinium Asian Screening Array (Illumina, CA, USA) at the time of this research. Whole blood-RNA-sequencing was performed for a subset of the genotyped samples (*n* = 500) and analyzed in this study. Stringent sample and variant level quality control (QC) filters were applied (e.g. sample call rate > 0.97, variant call rate > 0.99), resulting in *n* = 465 samples and *n* = 18,343,752 (including imputed) variants after imputation. The 465 samples were annotated with four levels of phenotype severity; “Most severe” for patients in ICU or requiring intubation and ventilation (*n* = 209), “Severe” for others requiring oxygen support (*n* = 150), “Mild” for other symptomatic patients (e.g. shortness of breath; *n* = 60), and “Asymptomatic” for those without COVID-19 related symptoms (*n* = 46). RNA-seq was performed using the NovaSeq6000 platform (Illumina, CA, USA) with paired end reads (read length of 100 bp), using S4 Reagent kit (200 cycles). We lifted over the hg19 genotypes to hg38 using GATK LiftoverVcf, and filtered out the ones without unique mapping. Further details about the sample collection, genotyping and RNA-seq data generation are described in ref. 19.

### RNA-seq data analysis and QTL calls

We followed the analysis pipeline provided by the GTEx^4^ [https://github.com/broadinstitute/gtex-pipeline], with minimal changes. Specifically, RNA-seq data was first aligned to hg38 human reference genome (excluding ALT, HLA and decoy contigs) using STAR v2.5.3a (for eQTL study) and STAR v2.6.0 (for sQTL study), with parameter ‘--sjdbOverhang 100ʼ instead of 75. Transcript amounts were quantified using RSEM v1.3.0. Sample QC was performed based on the metrics described in ref. 4 such as total number of mapped reads (Fig. S1). We changed the threshold of correlation statistics from *D*_i_ = −5 (as described in ref. 68) to −15, since we expect lower correlation between samples with different infectious disease severity status, resulting in *n* = 465 samples that were used for all the downstream analysis (of 500 samples with RNA-seq data, 472 samples passed the RNA-seq QC metrics, and seven samples were further filtered out based on genotyping QC metrics as described in ref. 19). Sex chromosomes were not included for QTL analysis. The splicing level was quantified using LeafCutter^25^ v0.2.7, with the same filtering criteria.

For cis-eQTL call, the gene expressions were TMM-normalized and genes with low expression level were filtered out as in ref. 4. Variants with minor allele frequency (MAF) smaller than 1% were filtered out, and fastQTL (https://github.com/francois-a/fastqtl) was run to obtain nominal *p* values against the null hypothesis that the genotype has no effect on the gene expression, for 105,142,365 cis variant-gene pairs (defined as distance to transcription starting site, dTSS smaller than 1 Mb), with 60 PEER factors (as recommended in ref. 4), sex and 5 genotype PCs included as covariates. For cis-sQTL call, 15 PEER factors were used as recommended in ref. 4.

For trans-eQTL call, tensorQTL v1.0.5 (https://github.com/broadinstitute/tensorqtl) was used to perform association tests for all the trans (i.e. dTSS > 1 Mb) genotypes-gene pairs across the genome filtered to MAF > 5%. We did not explicitly model the inflation of test statistics due to multi-mapping, but instead applied a relatively loose thresholding and relied on manual inspection to evaluate the validity of individual findings.

### Statistical fine-mapping of cis-QTLs

Statistical fine-mapping was performed for each of the genes (for eQTL) and introns (for sQTLs) harboring at least one variant with a *p* value lower than genome-wide significance threshold (5.0 × 10^−8^). We included rare variants of MAF <1% particularly in this step, although such rare variants were filtered out and not included in the other parts of the analysis. We did not use q-value based per-gene FDR obtained by grouped permutations and instead relied on nominal *p* values with a more stringent significance threshold as described in detail in Supplementary Note, although our analysis suggests that the choice would not affect our main findings (Fig. S6). For each of such eGenes and eIntrons, all the variants within 1 Mb of the transcription starting site (TSS) were included as the region of interests, and the in-sample LD was directly calculated and adjusted for all the covariates that were included in the eQTL discovery step, following the best practices described in refs. 23, 24, 69. Point estimations of the eQTL effect sizes and standard deviations from the fastQTL outputs were used to specify the marginal test statistics. Two fine-mapping tools, FINEMAP v1.3.1 and susieR v0.11.43 with default parameter settings were used to perform statistical fine-mapping. Since the output of FINEMAP and SuSiE does not always agree with each other (although they correlate very well; Fig. S5a) and each of them is thought to have potential false positives, the minimum PIPs from two algorithms were taken to represent the PIP for each variant-genes (or variant-introns). Especially, based on functional enrichment analysis, we expect our SuSiE fine-mapping result presents a higher number of false (and true) positives, and taking the minimum PIPs results in reduction of false positives (possibly at the expense of sensitivity; Fig. S5b, c). Additional characterization of statistical fine-mapping results in terms of its sensitivity to methodological choice are described in Fig. S5d–i and Supplementary Note.

For statistical fine-mapping of sQTLs, we applied the same pipeline to each variant-intron pair, where the intron was defined from the leafcutter algorithm (thus not necessarily corresponding to canonical intron annotated in databases one to one). Since the number of introns are larger than the number of variant-genes, we filtered out introns harboring more than 25,000 variants in 1 Mb window (typically those in major histocompatibility complex or other complex regions) to reduce the computational burden.

For colocalization analyses, the colocalization posterior probability (= CLPP) for each variant was defined as the product of two PIPs, regardless of the study samples identify (Supplementary Note).

### Annotation of QTLs

Association statistics in GTEx were obtained from the GTEx web portal (https://www.gtexportal.org/home/datasets; we only used whole blood data throughout the study). PIP and the expression modifier score (EMS) for the variants existing in GTEx were downloaded from ref. 23. Fine-mapping results of Biobank Japan is collected from ref. 32, and that from UK Biobank is from http://finucanelab.org/data. Population allele frequencies are annotated from the genome Aggregation Database (gnomAD) (http://gnomad.broadinstitute.org/). Those represented in hg19 (the Biobank Japan and UK Biobank data) were matched to our data using hg19 coordinates, and those in hg38 were matched to our data using hg38 coordinates that we lifted over.

To obtain splicing-related annotations for the variant-genes with non-trivial (>0.001) sQTL PIPs, we first took the maximum PIP of all the introns on the same gene. We then ran the Variant Effect Predictor (VEP) version 104 on the web interface (https://asia.ensembl.org/Homo_sapiens/Tools/VEP/), and took the maximum of delta scores for splice donor and acceptor loss/gain as a single representative value for SpliceAI score. We used ggsashimi (https://github.com/guigolab/ggsashimi) to visualize sQTL effects.

### Differential expression analysis

Differential gene expression analysis was performed using edgeR^2^ v3.34. All the genes that passed the expression level threshold in the QTL analysis were included in the analysis. Expected count data from RSEM was rounded and used as the input matrix. TMM-normalization was applied to calculate the normalization factor. The samples were classified as either severe (*n* = 359, those annotated as “Severe” or “Most severe”) or non-severe (*n* = 106, those annotated as “Mild” or “Asymptomatic”) group in a binary fashion. Log-likelihood ratio test (LRT) including sex and age in the generalized linear model was performed to quantify the effect size (fold change) and the significance (*p* value). We did not discretize the age, with an aim to capture possible continuous effects of age on gene expression, but the results were consistent otherwise.

Positively differentially expressed genes (= genes with increased expression in severe cases) were defined by p value less than (0.05/#genes) and log_2_FC > 1, and negatively differentially expressed genes were defined by the same *p* value threshold and log_2_FC < −1. GO term analysis was performed using g:Profiler web interface (https://biit.cs.ut.ee/gprofiler/page/citing version: e104_eg51_p15_3922dba).

As a comparison, we also tested different phenotype assignments (continuous four-rank severity, or comparing the subset of samples of most severe vs non-symptomatic), a different differential expression tool that uses median-ratio normalization (DEseq2 v1.32 https://bioconductor.org/packages/release/bioc/html/DESeq2.html), a different threshold to defined the differentially expressed gene set to test for GO term enrichment, and inclusion of inferred cell type composition (Fig. S16), all yielding roughly consistent results.

Differential splicing analysis was performed using a custom code. For each intronic region as part of the intron defined in leafcutter, the intronic usage fraction was calculated, standardized within an individual, and rank-normalized across introns as part of the leafcutter pipeline. We then performed LRT including COVID status, age and sex in a standard linear model (the likelihood from linear model was calculated in a closed form corresponding to a minimum least square) to calculate the nominal *p* value indicating the association between the COVID status and normalized intron usage. We applied a Bonferroni-corrected *p* value threshold (0.05/#intronic regions) for GO term enrichment analysis in g:Profiler web interface. The un-normalized intron usage (%) was used for visualization in Fig. S17.

### Comparison with existing COVID-19-associated genes and variants

COVID-19-associated genes were defined as the set of genes that are reported in ref. 13, which are in LD (within *r*^2^ > 0.6) or showing evidence of variant-to-gene connection with the variants with significant association *p* value in their study. We note that these genes are nominated solely on the basis of linkage with the variants associated with COVID-19 disease susceptibility or severity in their study, and thus does not indicate causal relationship by its own.

### Cell type specificity analysis

We downloaded the eQTL summary statistics as well as the count matrix for 28 cell types from the ImmuNexUT study^48^. Expression enrichment for a gene set G in a cell type C was defined as the average of count in cell type C across all the genes in G divided by the average of the count of genes in G across 28 cell types. i.e.

Expression enrichment (G, C) = $${\sum }_{g\in G}{{{{{\rm{Cnt}}}}}}(C)/{\sum }_{g\in G}{\sum }_{c\in C}{{{{{\rm{Cnt}}}}}}(C)/28$$.

The error bar represents the 95% confidence interval and was estimated by a bootstrap of 1000 repeats (sampling from the count matrix each time, allowing for replacement).

To define the eGenes in ImmuNexUT, we relied on marginal *p* value instead of FDR, and set the cutoff to be 5.0 × 10^−8^ to let it be consistent with the definition of eGenes throughout the analysis.

### Interaction eQTL (ieQTL) analysis

We used tensorQTL to perform ieQTL analysis. TensorQTL builds a linear model including the effect of genotype alone, interaction variable (COVID-19 phenotype severity in our case) alone, as well as the interaction between those two, and tests the significance of interaction term to obtain the *p* value (marginal, as well as the Benjamini-Hochberg adjusted ones). We did not apply inverse normal-transformation to the interaction variable (COVID-19 severity), since it is in discretized scale (ranging from 1 for non-symptomatic to 4 for most-severe). Same set of covariates as the eQTL call step were included (We did not include inferred cell type composition as covariates. Instead, we have confirmed that the inclusion still leads to consistent results; *r* = 0.91 in the scale of −log_10_(*p*) for 13 ieGenes; Fig. S23). The neutrophil ieQTLs summary statistics were downloaded from the GTEx portal. In order to quantify the direction concordance between two ieQTL summary statistics, we centered the COVID-19 severity value (to account for the inflation in the interaction term due to different distribution of the interaction variable), and then multiplied the effect size of the genetics term *β*_g_ and interaction term *β*_gi_. The positive value of this product indicates increasing variance of genetic effect along with the interaction variable (COVID-19 severity in JCTF, or estimated neutrophil score in GTEx). We define the effect direction to be concordant when the sign of this product matches between JCTF and GTEx (i.e. when severe COVID-19 corresponds to increase of neutrophils count estimation). We validated this quantification by confirming that when restricting to genes that are unlikely to be neutrophil ieQTLs in GTEx (adjusted *p* = 1, *n* = 11,945) the sign showed near 50% (49.7%) concordance (Supplementary Note). For cell type decomposition, we used CIBERSORT^54^ web interface (http://cibersort.stanford.edu/) with the built-in LM22 signature matrix as the reference and used the TPM matrix of JCTF as the input matrix.

### Statistical analysis

All the statistical tests were two sided. No adjustment was made for the *p* values we report, unless it is clearly stated as “adjusted *p* value”. Error bar denotes the standard error of the mean unless noted otherwise. For standard error estimation of Pearson correlation (Fig. 2f), Fisher’s z-transformation^70^ was used. Enrichments of a category C1 in category C2 (Figs. 2h, 3d, 4c) were defined as the probability of drawing a variant-gene pair of C1 given that the variant-gene is in C2, divided by the overall probability of drawing a variant-gene pair of C1 (i.e. $\frac{p({vg}\in C1|{vg}\in C2)}{p({vg}\in C1)}$). The error bar of enrichment denotes the standard error of the numerator, divided by the denominator.

The set of softwares and tools used for the analysis as well as data visualization are listed as below;

CIBERSORT web interface (http://cibersort.stanford.edu/)

DESeq2 v1.32.0 (https://bioconductor.org/packages/release/bioc/html/DESeq2.html)

edgeR v3.34 (https://bioconductor.org/packages/release/bioc/html/edgeR.html)

fastQTL v2.165 (http://fastqtl.sourceforge.net)

FINEMAP v1.3.1 (http://www.christianbenner.com/)

GATK v4.1.9.0 LiftoverVcf (https://gatk.broadinstitute.org/)

ggsashimi v.1.1.0 (https://github.com/guigolab/ggsashimi)

g:Profiler web interface (https://biit.cs.ut.ee/gprofiler/page/citing)

GTEx pipeline (https://github.com/broadinstitute/gtex-pipeline)

LeafCutter v0.2.7 (https://davidaknowles.github.io/leafcutter/index.html)

matplotlib v3.3.4 (https://matplotlib.org)

numpy v1.20.1 (https://numpy.org)

pandas v1.1.4 (https://pandas.pydata.org)

RSEM v1.3.0 (https://deweylab.github.io/RSEM/)

scikit-learn v0.24.1 (https://scikit-learn.github.io/stable)

scipy v1.6.2 (http://scikit-learn.github.io/stable)

seaborn v0.11.1 (https://seaborn.pydata.org)

STAR v2.5.3a and v2.6.0 (https://github.com/alexdobin/STAR)

susieR v0.11.43 (https://github.com/stephenslab/susieR)

tensorQTL v1.0.5 (https://github.com/broadinstitute/tensorqtl)

Variant Effect Predictor (VEP) version 104 web interface (https://asia.ensembl.org/Homo_sapiens/Tools/VEP/)

### Reporting summary

Further information on research design is available in the Nature Research Reporting Summary linked to this article.

## Supplementary information

Supplementary Information

Description of Additional Supplementary Files

Supplementary Data 1-10

Reporting Summary

## Data Availability

The summary statistics of eQTL (cis and trans) and cis-sQTL analysis, as well as the RNA-seq expression matrix^19^ are available at the National Bioscience Database Center (NBDC) Human Database (accession code: hum0343.v2). The individual genotype data^19^ is available at European Genome-Phenome Archive (EGA) (accession code: EGAS00001006284). The data from colocalization, differential expression and ieQTL analysis in this study are provided in the Supplementary Data file. The list of publicly available datasets used are listed below: Biobank Japan (BBJ) and UK Biobank (UKB) fine-mapping: NBDC Human Database (accession code: hum0197) and https://www.finucanelab.org/data eQTLgen trans-eQTL data: https://www.eqtlgen.org/trans-eqtls.html The expression modifier score (EMS): https://www.finucanelab.org/data Genome Aggregation Database (gnomAD) allele frequencies: https://gnomad.broadinstitute.org/downloads Genotype-Tissue Expression (GTEx) cis-eQTL data: https://gtexportal.org/home/datasets ImmuNexUT cell type expression data: https://www.immunexut.org Multi-Ethnic Study of Atherosclerosis (MESA) cis-eQTL data: https://www.dropbox.com/sh/f6un5evevyvvyl9/AAA3sfa1DgqY67tx4q36P341a?dl=0.

## Code availability

The code used in this manuscript is available at https://github.com/QingboWang/japan_covid_taskforce_rna.
