# Supplementary material for: The whole blood transcriptional regulation landscape in 465 COVID-19 infected samples from Japan COVID-19 Task Force
Source: Nat Commun. 2022 Aug 22;13:4830. doi: 10.1038/s41467-022-32276-2 (PMC9395416; doi:10.1038/s41467-022-32276-2)
Supplement: Supplementary file 1 — Supplementary Information [file 41467_2022_32276_MOESM1_ESM.pdf]

The whole blood transcriptional regulation landscape in 465 COVID-19 infected samples from Japan COVID-19 Task Force

Wang et al. 2022

Supplementary Information

Supplementary Note: pages 2 to 7
Supplementary Tables: pages 8 and 9
Supplementary Figures: pages 10 to 43
Supplementary References: pages 44 and 45

Supplementary Note

Determining the significance threshold in p-value and PIP

We used a uniform threshold of p-value = 5.0×10^{-8} for most part of our analysis to discuss significant association, although we recognize this is thought to be over- or under- conservative depending on the analysis. Specifically, for trans-eQTL analysis, due to large number of multiple testings, the Bonferroni-corrected threshold $p=3.6\times10^{-13}$ is orders of magnitudes smaller than the 5.0×10^{-8} threshold, and we use the wording of "possible" trans-eQTLs to convey the nuance. Although many studies, including GTEx[1], uses False Discovery Rate (FDR) based on Benjamini–Hochberg procedure or other assumptions, we thought a uniform p-value threshold is less dependent on the significance of other variants tested on the same gene (similar to the idea discussed in [2]) and would streamline with the downstream fine-mapping procedure which takes nominal p-value as the input (We included the FDR threshold provided by GTEx in **Fig. 2b, d**, and used FDR-adjusted p-value as the x axis in **Fig. 7f** for visually easier interpretation).

The posterior inclusion probability (PIP) is thought to be a measure less susceptible to the number of variants (or the number of tests), but the extent it corresponds to truly "being causal" (as well as the strict definition of "being causal" itself) is still unclear. For example, a variant with PIP being exactly 1 can still easily be non-causal when the model is mis-specified. Although we used PIP>0.9 as a threshold for "putative" causal, PIP=0.1 or CLPP=0.1 as "possibly" causal, we note that the choice of the word is subjective.

Overall we do not intend to make binary discussion of whether an association is "truly significant" or not, and instead would like to confer a continuous spectrum of association signal strength.

Choosing the genes to apply fine-mapping algorithms

Choosing the p-value threshold matters most when deciding on the genes to apply fine-mapping algorithms. Following [3], we performed fine-mapping on the subset of genes passing a hard cut-off of minimum 5.0×10^{-8}. This is equivalent to setting a discretized distribution of the prior on the number of causal variants on a gene; for example, fine-mapping algorithms will be applied to a gene A with minimum $p=5.0\times10^{-8} - \delta$, but will not be applied to another gene B with minimum $p=5.0\times10^{-8} + \delta$ (here, let δ be a very small number). Gene A will have a large prior probability of harboring at least one causal variant (which is an assumption fine-mapping algorithms typically make), while gene B will essentially have zero probability of harboring causal variants (since fine-mapping algorithms will not be applied at all) under our genome-wide fine-mapping strategy, even though intuitively the biological differences between gene A and B is subtle.

While we would not provide a full characterization and possible solutions of such observation in detail, we note that future work on this direction would be highly valuable (e.g. having a continuous prior of a gene harboring at least one causal variant, as a function of its minimum p-value as well as other parameters such as LD matrix).

Quantification of the Posterior Inclusion Probability (PIP) from two algorithms
As described in **Methods**, we used SuSiE and FINEMAP with default parameter setting and took the minimum PIP to assign one PIP for each variant-gene. Here we describe the result when using two slightly different approaches.

First is changing the number of single effect vectors (l) in SuSiE. SuSiE sets $l = 10$ by default whereas the maximum number of causal variants per region in FINEMAP is set to a smaller number, 5. We tested the setting of $l = 5$ in SuSiE to make it consistent with FINEMAP and compared the results (**Fig. S5d**). As expected, this resulted in deflation of PIP, reducing the number of p-causal eQTLs to 68.6% (from 478 to 328, where 292 were consistent) in chromosome 1. Based on this result we assume that setting l=10 in SuSiE for eQTL fine-mapping might leads to larger number of true and false positives, but would leave the discussion of best parameter setting to future work. We also tested setting the maximum number of causal variants per region in FINEMAP to be 10, and the result was much more stable (130 versus 138 p-causal eQTLs; **Fig. S5e**). We stuck with the default parameter setting rather than modifying the parameter to make it consistent between two methods.

Second is taking the mean PIP from two methods as done in [3] rather than taking the minimum as we did ([3] further excluded the variants with PIP difference > 5% in their analysis). We compared the mean and minimum PIP distribution, including those that would be excluded in their analysis (**Fig. S5f-i**). As expected (and by definition), taking the minimum resulted in more conservative estimation. Taking the mean and excluding those with >5% difference resulted in higher agreement with GTEx results (215 p-causal eQTLs in GTEx out of 391 in JCTF. 215/391 = 55.0%), but when including those with >5% PIP difference, the agreement level (234 out of 564 = 41.6%) was slightly lower than when taking the minimum (233 out of 505 = 46.1%).

In summary we observed that different approach results in slightly different but largely consistent PIP distributions, validating our approach as one of the “good” practices. We would leave further studies to explore the “best” practices in merging the PIP estimation from different fine-mapping algorithms each with different possible parameter choices as a future work.

Details of putative causal cis-eQTLs colocalizing with BBJ or harboring trans-eQTL effects
Here we provide additional descriptions of the colocalizing variants presented in **Fig. 4. b-d**. The first example, rs3809627 (chr16_30091839_C_A), identified as a

putative causal eQTL for *TBX6* (PIP=1), is a likely-causal variant for red blood cell (RBC) count in BBJ (PIP=0.86; **Fig. 4b**). The variant, located at 5'UTR of the *TBX6* gene, is experimentally confirmed to lower the expression of *TBX6* gene in a reporter assay[4], and also shows high PIP (PIP=1) in GTEx and UK Biobank = UKB[3,5,6] supporting our finding.

The second example, rs11082304, an intronic variant on the *CABLES1* gene well-known for its strong association with platelet count (Plt)[6-8], was putatively causal for both *CABLES1* expression in JCTF and platelet count in BBJ (PIP>0.99 in both, resulting in CLPP>0.99; **Fig. 4c**), suggesting the role of *CABLES1* in platelet production. Notably, the variant does not reach genome-wide significance in GTEx Whole Blood data[1], demonstrating the increased power of colocalization (We also note that the same variant-gene harbored weaker colocalization signal for Muscle Skeletal tissue in GTEx with PIP = CLPP = 0.34). A study[9] showed *CABLES1* plays a key role in hematopoiesis specifically in aged mice, although knocking out the *CABLES1* gene did not result in detectable differences in platelet count in their analysis. The third example, rs2902548 (chr10_102727625_C_T), is explained in the main text.

For the trans-eQTL examples provided in **Fig. 5d**, *REST* gene is a known suppressor of neuronal gene expression in non-neuronal tissues. These three genes are all known to be target gene of *REST*[10], are highly expressed in brain (median TPM>30 in Brain - Cerebellum in GTEx v8), and are up-regulated by cis-eQTLs on the *REST* gene (β = 0.284, 0.262 and 0.219 for the lead cis-eQTL of *REST*), consistent with the hypothesis presented in the eQTLgen study that the down-regulation of *REST* (β = -0.229 for the lead cis-eQTL effect on *REST*) results in up-regulation of its target genes. Descriptions about the *STING1* example in **Fig. 5e** is provided in the main text.

Choice of the method for colocalization analysis

We used a naïve approach of taking the product of two PIPs to derive the colocalization posterior probability[11] (= CLPP) in colocalization analysis, whether it is eQTL-sQTL colocalization from the same samples or eQTL-complex trait colocalization using external samples. We acknowledge that the PIPs of the pairs of traits, especially when coming from the same study samples, are subject to the same source of errors or biases, making them non-independent and thus the assumption of derivation of CLPP in the original method [11] is violated. We assume our approach is simple and conservative one. Future studies could use colocalization methods with more flexible, non-independent assumptions such as in [12].

Details of putative causal cis-eQTLs on possibly COVID-19 related genes

As described in the main text, we identified 11 variants that are potentially regulatory to total 9 genes through gene expression regulation (PIP>0.5) but think these results do not nominate phenotype-causal variants or genes with high confidence. For

example, although our analysis nominates 3' UTR variant rs576605913 (chr8_124307452_T_TA) as the putative causal eQTL (PIP=0.97) for *TMEM65* (**Fig. S11a**), the variant is no longer of significance in the latest release (i.e., release 6) of the COVID-19 HGI analysis. As another example, we identified an intronic variant rs901886 (chr19_10291455_T_C) as possible causal eQTL increasing *ICAM5* expression (PIP=0.80; **Fig. S11b**), but latest studies suggest that the signal from the proximal to *ICAM5* is caused by a known coding variant rs34536643 on *TYK2*, rather than the *ICAM5*. In addition, these putative causal eQTLs do not match the GWAS lead variants (rs72711165 and rs4801778, dotted line in **Fig. S11**). Other nine variants also lack strong evidence for phenotypic causality. Our results overall highlight the challenges of trying to identify the causal association of host genetics and COVID-19 severity mediated by gene expression.

Details for evaluation of trans-eQTLs in eQTLgen database

The fact that the variants present cis-eQTL effect in our dataset increases the chance of being trans-eQTLs in eQTLgen, and the chance increase by the conditioning was present also for variants with possible trans-eQTLs effects in our dataset (green and red dots in **Fig. 5b**). This observation suggests trans-eQTL effects mediated by cis-eQTL effects as one of the major machanisms[13,14], but also could be due to the fact that the variants tested in eQTLgen is ascertained for trait associated variants. To evaluate these two factors, we stratified the variants with the maximum PIP of cis-eQTL effects, and investigated the enrichment of two groups; 1. All the variants tested in eQTLgen (n=10,317), and 2. the subset of these that were identified as trans-eQTLs (FDR<0.05, n=3,853) (**Fig. 5c**). The first group was enriched for high-PIP variants (diamond shape in **Fig. 5c**), reflecting the ascertainment effect. Notably, the second group showed significantly higher enrichment (circle shape in **Fig. 5c**), suggesting that variants with putative causal cis-eQTL effects has higher chance of presenting trans-eQTL effects, and the fact is not simply due to ascertainment in eQTLgen.

A plausible mechanism for decreased power of eQTL calls for genes highly expressed in severe COVID-19 cases

The fact that severe COVID-19 cases decrease the power to call eQTLs could be counterintuitive at first site, but could be expected as below:
A lot of genes highly/lowly expressed in severe COVID-19 cases are related to immune response (i.e. most of their expression changed in response to the infection, as noted in [15]), and although we did find a few genes with ieQTLs effects, most of the overall expression change, originating from fundamental biological processes, are near-independent from the host's genotype (also as mentioned in [15]). In other words, if we decompose the variation of gene expression as $V(total) = V(g) + V(e)$, where g is the host's genotype, and e is the environment (which, importantly, includes COVID-19 case status), the $V(e)$ becomes much larger for genes

highly/lowly expressed in severe COVID-19 cases (since the environment, which is COVID-19 infection, has huge effect on the expression of those genes). Thus, the expression variation explained by the host's genotype ($V(g)/V(total)$) would be much lower in those genes, and would be harder to detect with statistical significance. Relative effect of $g \times e$ interaction is weak and will not change the discussion above.

Defining the direction of interaction eQTLs

In tensorQTL[16], interaction eQTLs (ieQTLs) were analyzed by testing the significance of the effect size β_{gi} of the interaction term in a linear model including the genotype, interacting variable as well as the interaction:

$$y \sim x \cdot \beta_g + I \cdot \beta_i + x \cdot I \cdot \beta_{gi} + covariates + \epsilon$$

In a setting where we would like to test the direction concordance of the interaction eQTLs effect for a gene in two datasets,

$$y_1 \sim x_1 \cdot \beta_{1,g} + I_1 \cdot \beta_{1,i} + x_1 \cdot I_1 \cdot \beta_{1,gi} + covariates + \epsilon_1$$
$$y_2 \sim x_2 \cdot \beta_{2,g} + I_2 \cdot \beta_{2,i} + x_2 \cdot I_2 \cdot \beta_{2,gi} + covariates + \epsilon_2$$

the simplest scenario is when the association statistics are available for the same genotype (i.e. $x_1 = x_2$), and the interaction terms follow the same distribution (i.e. $I_1 =^d I_2$). In such cases, the sign of each of the interaction term effect sizes directly allows us to interpret and compare the effect size direction (e.g., when $\beta_{1,gi} > 0$ and $\beta_{2,gi} > 0$, the increase of both two interaction variables increases the magnitude of expression increase in samples harboring alternative alleles compared to reference alleles).

In our analysis, such simple comparison is not available, for two reasons (1) the variant harboring strong ieQTL effect might be different in JCTF and GTEx, and (2) the distribution of two interaction variables (COVID-19 severity in four levels in JCTF versus estimated, normalized neutrophil score in GTEx) is different.

For (1), we simply tried to avoid the complication by taking the product $\beta_g\beta_{gi}$instead of simple $\beta_{gi}$for comparison (i.e. when the genotype markers with opposite marginal eQTL effect directions were chosen by chance, the interaction term sign would also be the opposite, and thus the effect would be canceled out by taking the product). That is, $\beta_g\beta_{gi} > 0 \; and \; \beta_g > 0$ is essentially the same as $\beta_g\beta_{gi} < 0 \; and \; \beta_g < 0$ other than the (arbitral) choice of marker genotype. Although this assumption fails when the sign of the point estimate of one of the effect sizes $\beta_g$or $\beta_{gi}$was different from that of the true effect sizes, we assume such a situation is very less likely to happen unless the true effect size is very weak (which is not likely the case for ieQTLs that can be captured with current sample sizes).

For (2), since matching the distribution between four-level discrete values (the COVID-19 severity $I_1 \in \{1,2,3,4\}$) and continuous values (the normalized and inferred neutrophil score in GTEx $I_2 \sim N(0,1)$) is unrealistic, we instead chose to mean-center the COVID-19 severity term, to at least let the interpretation of the sign be concordant across two ieQTL analysis.

As written in the method, we validated this quantification in terms of calibration of false-positives by confirming that when restricting to genes that are unlikely to be neutrophil ieQTLs in GTEx (adjusted p=1, n=11,945) the sign showed near 50% (49.7%) concordance. For replication in JCTF using CIBERSORT, we did not need to apply this conversion since we have the information for same variant (i.e. we simply used the product of $\beta_{1,gi}$ and $\beta_{2,gi}$).

Shared eQTL and ieQTL signals

As described in the main text, we performed fine-mapping of the eQTL and ieQTL signals separately for the 13 possible ieGenes (adjusted p<0.05) to clarify whether there are distinct causal variants underlying eQTL and ieQTL signals in a gene (**Fig. S21, 22**), and did not find strong evidence of such distinct causal variants, although this could be due to limited resolution of fine-mapping. For example, the putative causal variant was shared for eQTL and ieQTL in *CLEC4C*, and fine-mapping suffered from tight LD in *MYBL2*; **Fig. S21**). There was a clearly distinct ieQTL association signal peak at near 1Mb upstream of *TEX30* (**Fig. S22**), but we note that this requires further investigation (although reports analyzing *TEX30* in the context of viral infection, basophil activity, allergy and CpG methylation exist[17-19] biological characterization of the association signal remains challenging, leaving the possibility of it being an artifact). For 12 out of 13 genes other than *TEX30*, the eQTL and ieQTL peak seemed to be in the same locus.

Confounding factors for COVID-19 phenotype assignment

As described in detail in [20], the study samples vary not only in annotated information such as COVID-19 severity status, age, sex, but also additional factors that are highly correlated. For example, almost all of the samples in the most-severe category have undertaken steroids as an emergency treatment. Although we believe most of the observations we reported are mainly due to COVID-19 disease status rather than the drug administration, based on biological support that are discussed in the main text, we note that we cannot fully rule out the causal contribution of such additional factors.

Supplementary Tables

Supplementary Table 1. The numbers of variant-genes, variants and genes in the main analyses

Analysis	Analysis category*1	Method	variant-genes	variants	genes	threshold
**cis-eQTL**	1	association	1,314,278	787,597	8,199	$p<5.0\times10^{-8}$
		fine-mapping	1,169	1,059	1,096	PIP>0.9
**cis-sQTL**	1	fine-mapping*2	2,387	1,278	2,201	PIP>0.9
**cis-e/sQTL coloc**	1	colocalization	422	415	334	CLPP>0.1
**cis-eQTL BBJ coloc**	1	colocalization	26*3	23	23	CLPP>0.1
**Trans-eQTL**	1	association	73,057	51,516	1,736	$p<5.0\times10^{-8}$ (Discovery, above Bonferroni threshold)
**Differential gene expression**	2	fold change (FC)	-	-	198 (up)<br>10 (down)	$p<2.5\times10^{-6}$<br>$1<\lvert\log_2 FC\rvert$
**Differential intron usage**	2	fold change (FC)	-	-	73 (up)<br>0 (down)	$p<2.5\times10^{-6}$<br>$1<\lvert\log_2 FC\rvert$
**COVID-19 ieQTL**	3	association	13	13	13	FDR<0.05

*1 Analysis category corresponds to those in **Fig. 1**
*2 counts in the unit of variant-intron clusters
*3 Corresponding to 34 unique variant-gene-trait pairs

Supplementary Table 2. Summary of the significance tests in the manuscript

Analysis	Analysis category *	Number	Method	Main figures	Note
cis e/sQTL	1	$p<5.0\times10^{-8}$	Nominal p	Fig. 2-4, 5b, 7ab	We also used q-value based FDR for comparison; see **Fig. S6**
trans-eQTL (discovery)	1	$p<5.0\times10^{-8}$	Nominal p	Fig. 5a-c	We note this is a "loose" filtering
trans-eQTL validation (eQTLgen)	1	FDR<0.05	permutation-based	Fig. 5b-c	Data obtained from Vosa et al[13]. See Vosa et al. for details.
trans-eQTL (inspection)	1	$p<3.6\times10^{-13}$	Bonferroni	Fig. 5d,e	0.05 / #(test), where #(test) >> 10^{10}.
Differential gene expression	2	$p<2.5\times10^{-6}$	Bonferroni	Fig. 6b,c	0.05 / #(gene), where #(gene) = 19,913. Note that also fold change filtering was applied
GO term enrichment	2	p_adj<0.05	g:SCS algorithm	Fig. 6b	See gprofiler[21] for details.
COVID-19 ieQTL	3	FDR<0.05	Benjamini-Hochberg (= BH)	Fig. 7c-f Fig. 8	FDR based on BH method. (10 genes passed $p<5.0\times10^{-8}$)

* Analysis category corresponds to those in **Fig. 1**

Supplementary Figures

Supplementary Figure 1. Quality control of the RNA-seq data

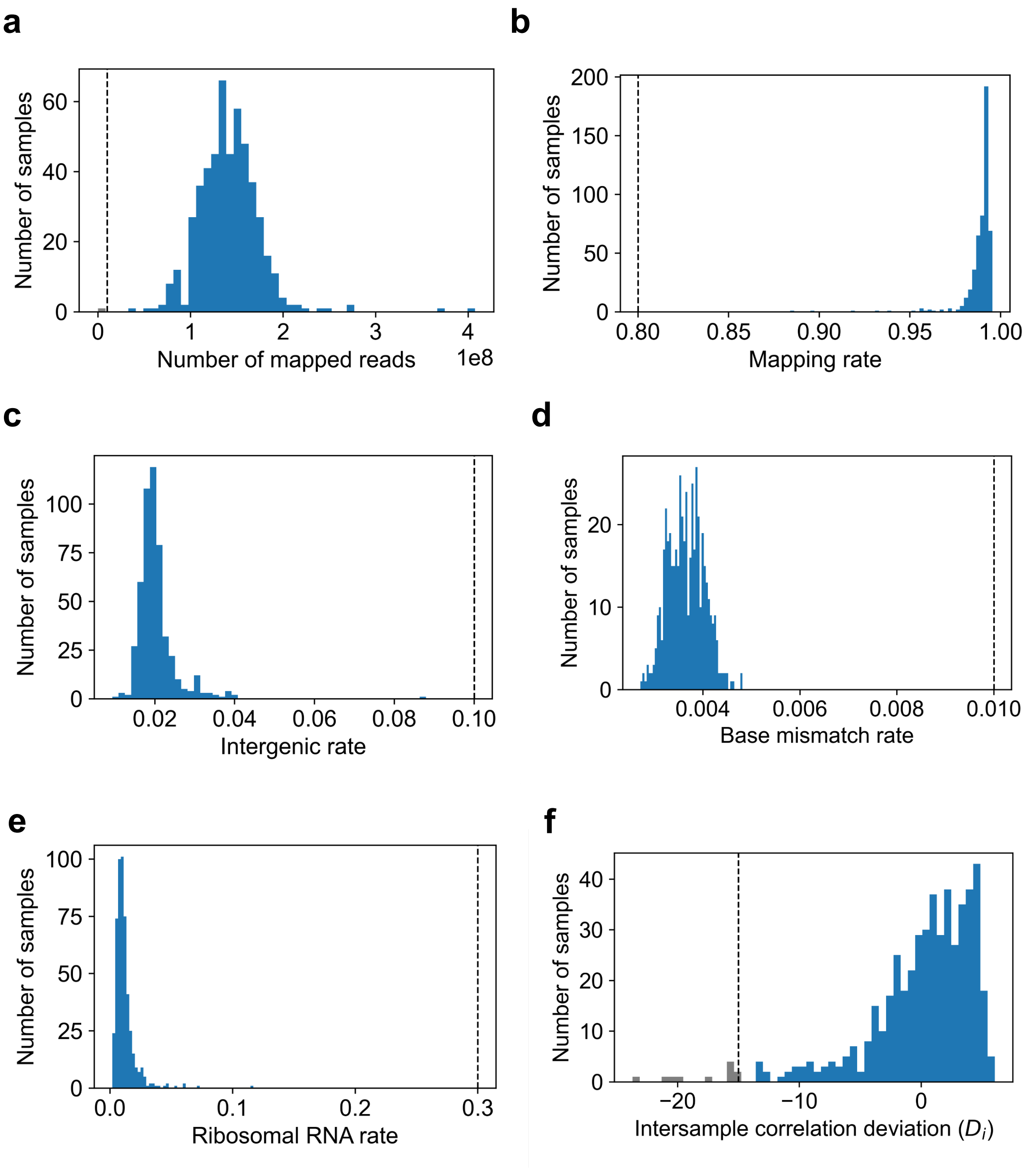

Filtering steps were applied to 500 Samples RNA-seq data based on number of mapped reads (>10^7), mapping rate (>0.8), intergenic rate (<0.1), base mismatch rate (<0.1), ribosomal RNA rate (<0.3), and the intersample correlation deviation metric (>-15). Dashed line corresponds to the threshold, and the gray color corresponds to filtered out samples.

Supplementary Figure 2. COVID-19 severity has minimum effect in eQTL call

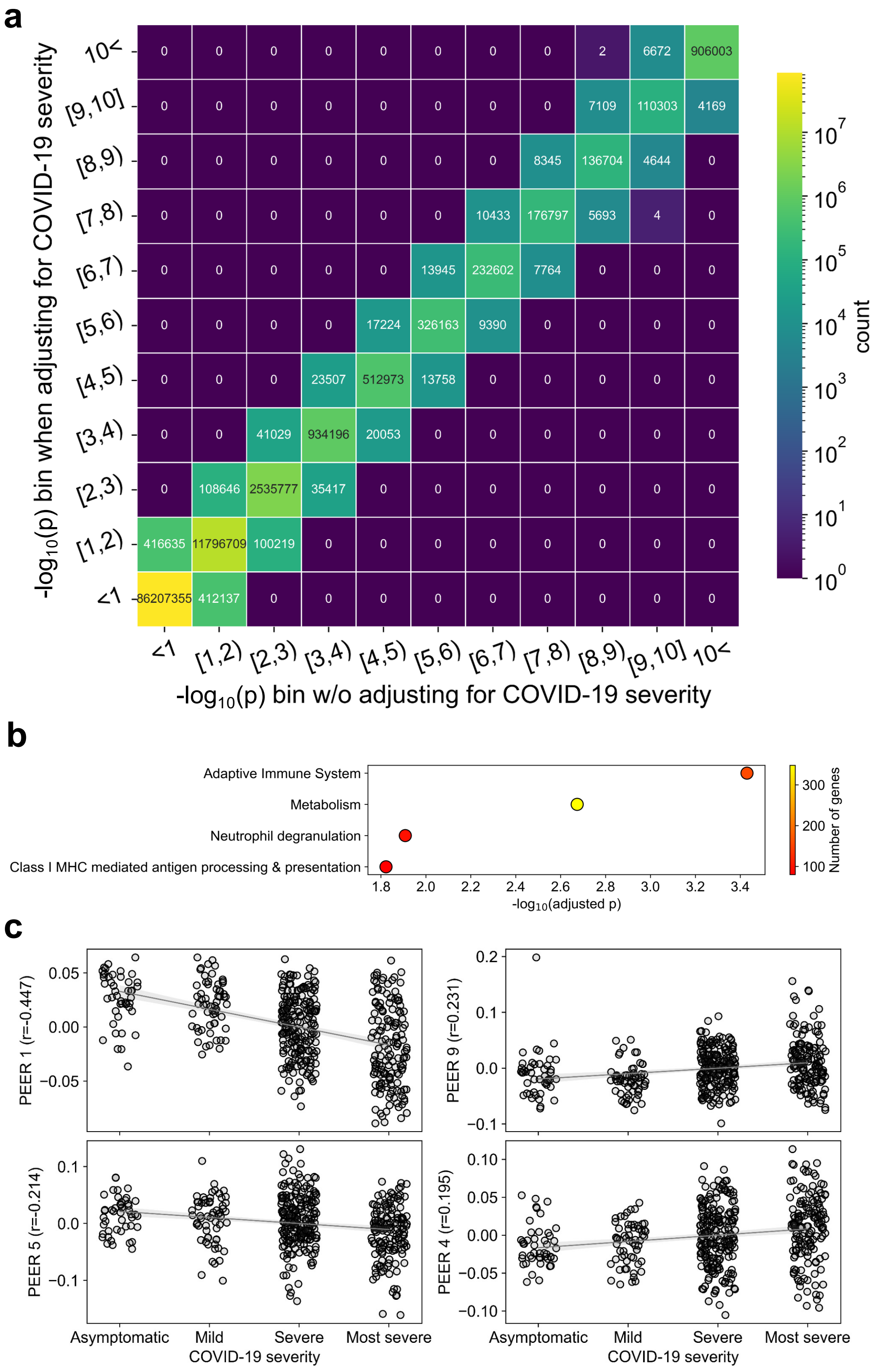

a. Comparison of binned association -log$_{10}$(p) value between eQTL call (x axis = without including COVID-19 severity as a covariate, y axis = including COVID-19

severity as a covariate). **b**. GO term (Reactome) enrichment for genes including variants below or above 5.0×10^{-8} threshold only before the adjustment (n=3,195). Data was generated from g:Profiler web (https://biit.cs.ut.ee/gprofiler/page/citing version: e104_eg51_p15_3922dba) [21]. **c.** Examples of PEER factors correlated with COVID-19 severity metric. Pearson correlation between the factor and the severity is shown in y axis label.

Supplementary Figure 3. Examples of significant correlations between PEER factors and age or inferred cell type compositions

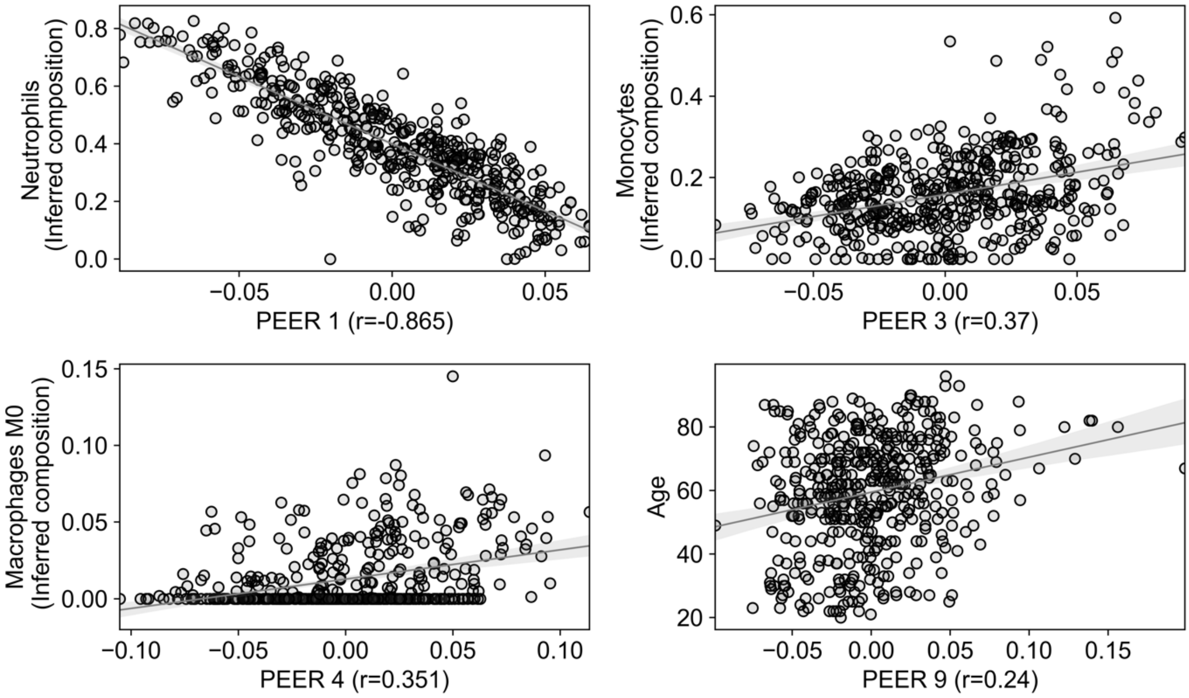

Scatter plots for visualizing the correlation between PEER factor 1 and inferred neutrophil composition **(top left)**, PEER factor 3 and inferred monocyte composition **(top right)**, PEER factor 4 and inferred macrophage composition **(bottom left)**, and PEER factor 9 and age **(bottom right)**, suggesting PEER factors are capturing meaningful expression variations (without information from the genotype data) as they are designed to. The Pearson correlations are shown in x axis labels. Error band denotes the 95% confidence interval. The full correlation matrix is available as **Supplementary Data 1**.

Supplementary Figure 4. Characterizing eQTL call and fine-mapping results by allele frequencies

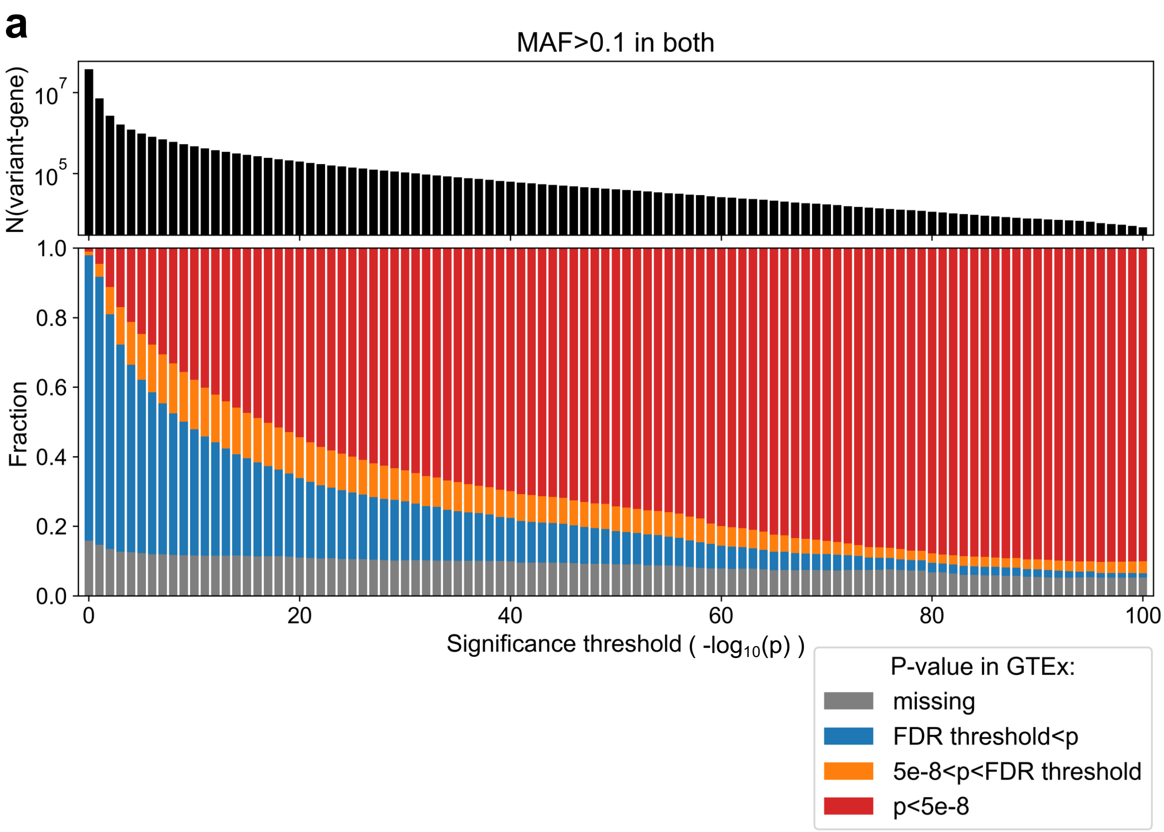

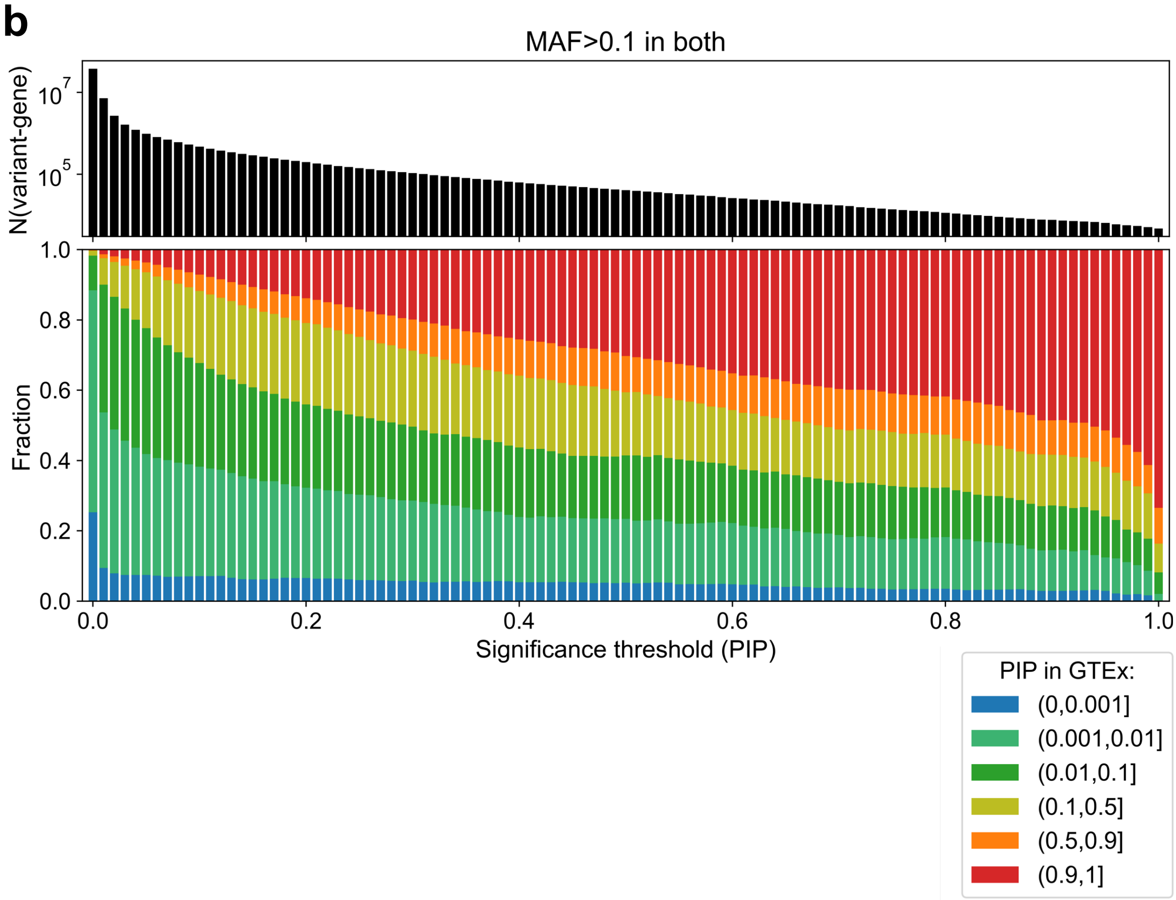

c

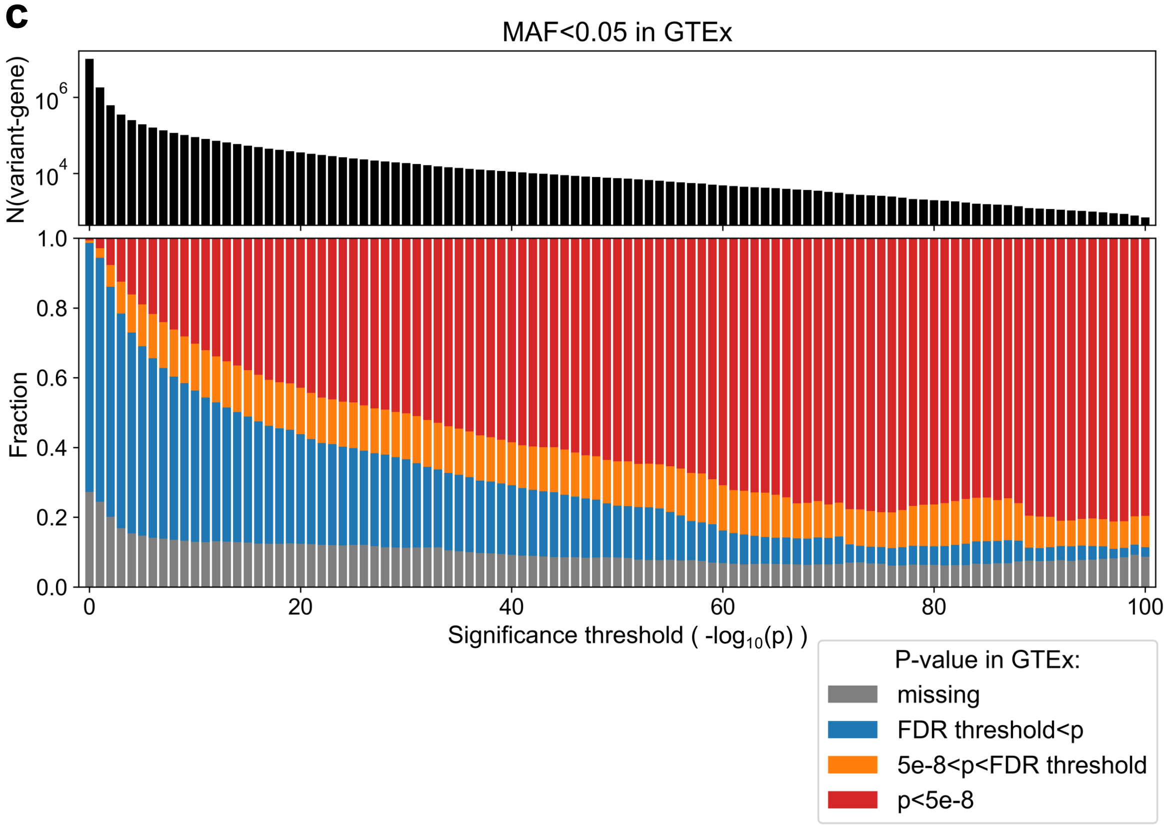

MAF<0.05 in GTEx
N(variant-gene)
10^6
10^4
1.0
0.8
0.6
0.4
0.2
0.0
Fraction
0
20
40
60
80
100
Significance threshold (-log10(p))
P-value in GTEx:
missing
FDR threshold<p
5e-8<p<FDR threshold
p<5e-8

d

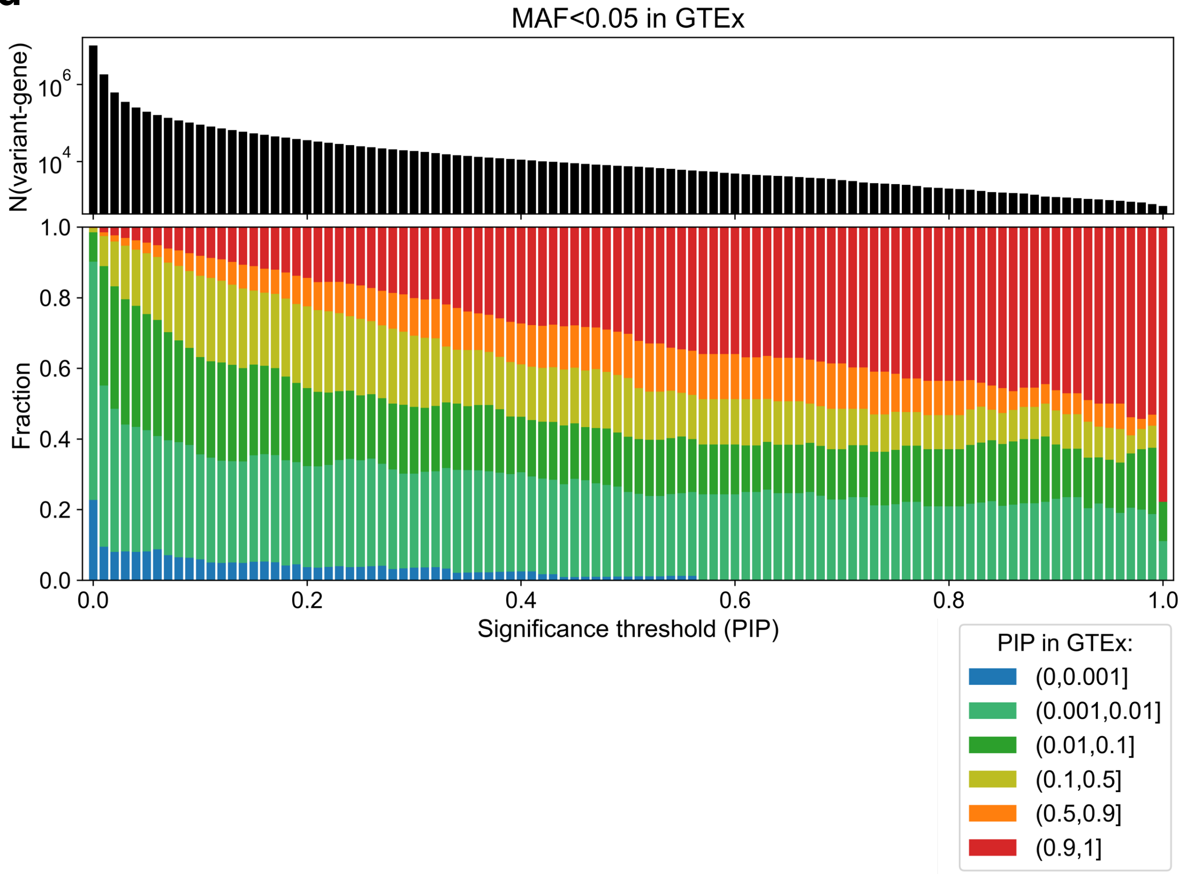

MAF<0.05 in GTEx
N(variant-gene)
10^6
10^4
1.0
0.8
0.6
0.4
0.2
0.0
Fraction
0.0
0.2
0.4
0.6
0.8
1.0
Significance threshold (PIP)
PIP in GTEx:
(0,0.001]
(0.001,0.01]
(0.01,0.1]
(0.1,0.5]
(0.5,0.9]
(0.9,1]

e

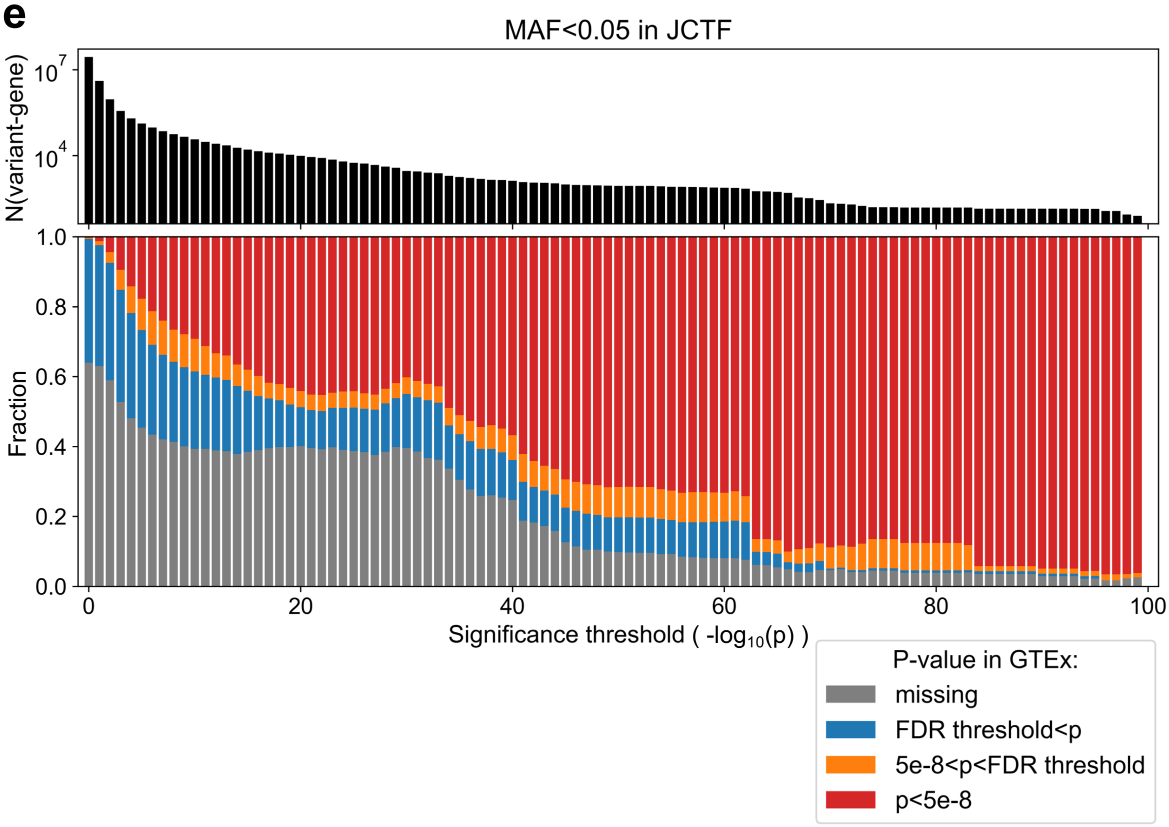
MAF<0.05 in JCTF
N(variant-gene)
10^7
10^4
1.0
0.8
0.6
0.4
0.2
0.0
Fraction
0
20
40
60
80
100
Significance threshold ($-\log_{10}(p)$)
P-value in GTEx:
missing
FDR threshold<p
5e-8<p<FDR threshold
p<5e-8

f

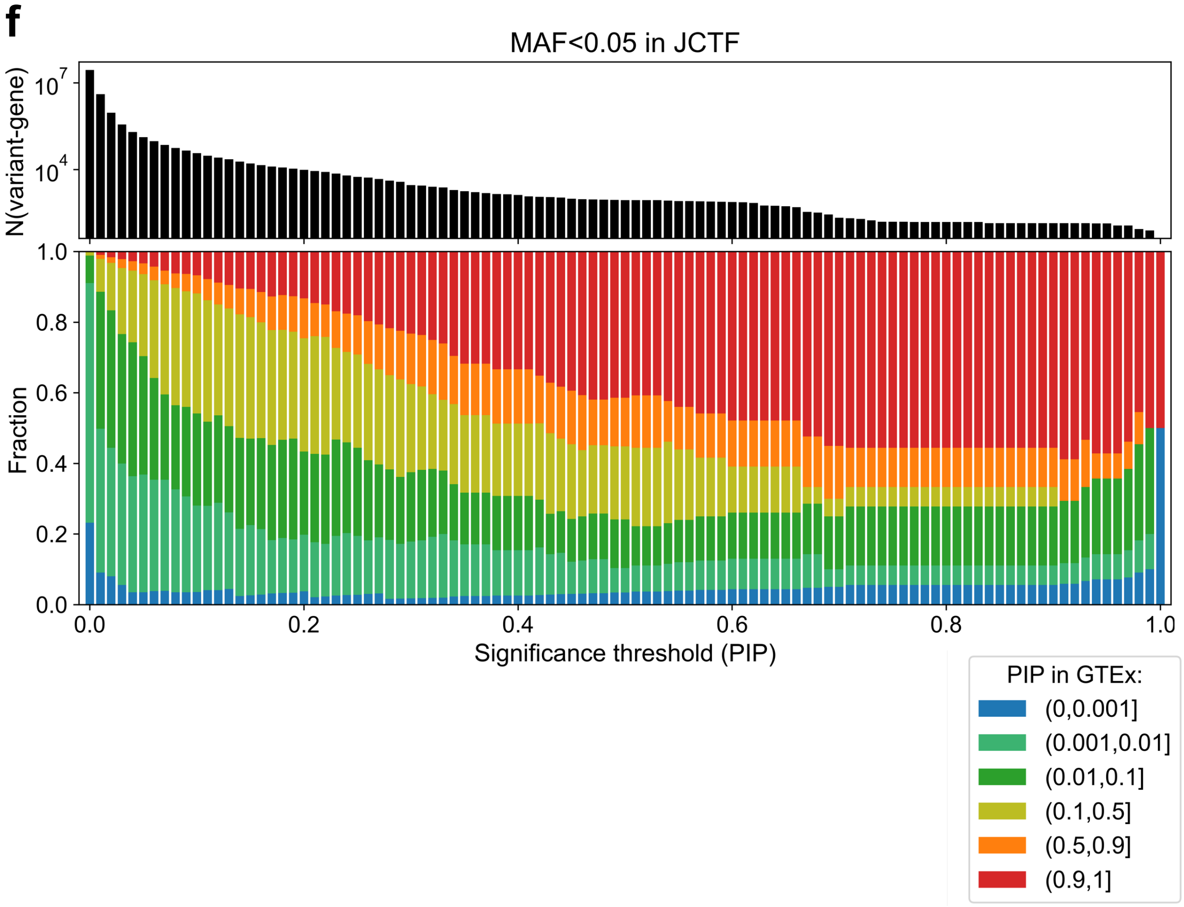
MAF<0.05 in JCTF
N(variant-gene)
10^7
10^4
1.0
0.8
0.6
0.4
0.2
0.0
Fraction
0.0
0.2
0.4
0.6
0.8
1.0
Significance threshold (PIP)
PIP in GTEx:
(0,0.001]
(0.001,0.01]
(0.01,0.1]
(0.1,0.5]
(0.5,0.9]
(0.9,1]

g

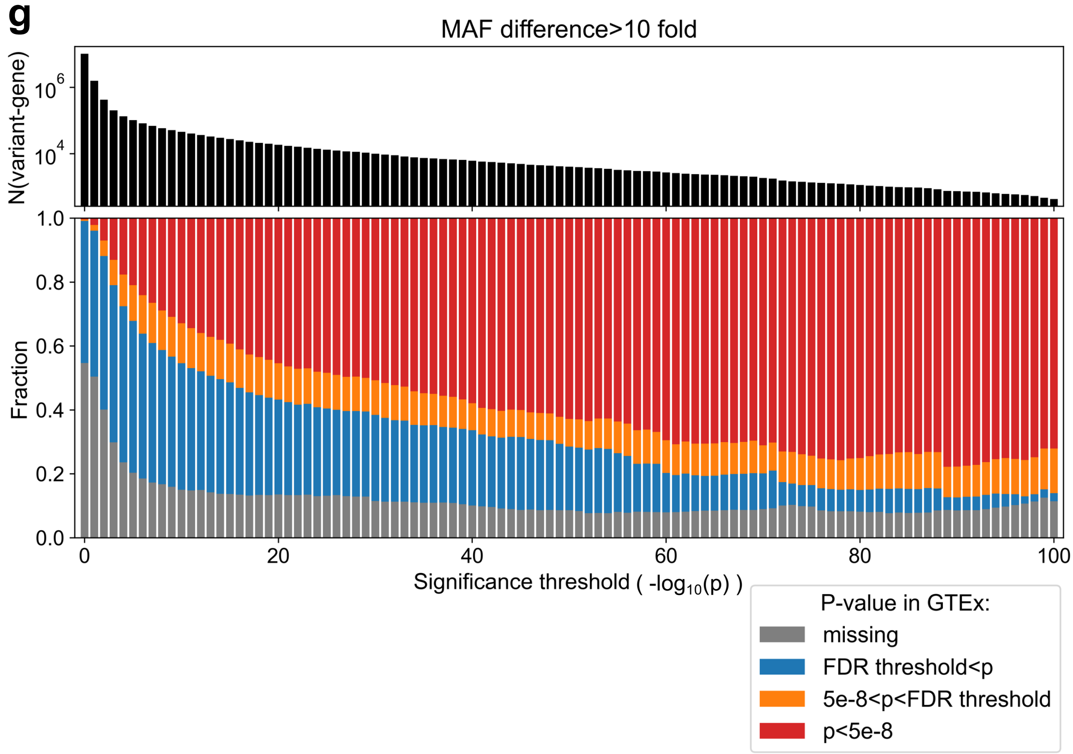

h

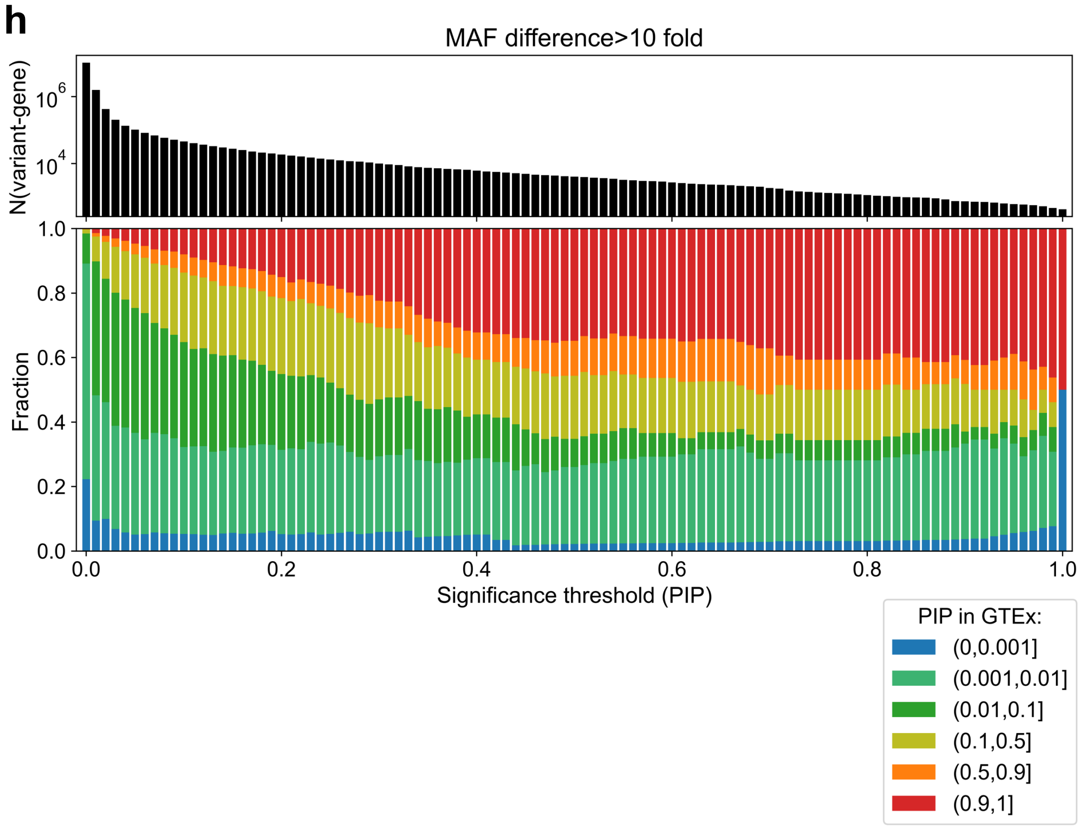

The number and the fraction of variant-genes classified into different p-value (**a,c,e** and **g**. 5e-8 = 5.0×10^{-8}.) or PIP (**b,d,f** and **h**) bins in GTEx v8, for different minor allele frequency (MAF) conditions. The results overall highlight the fact that MAF is one of the main factors for differences in p-value or PIP between populations in eQTL analysis, and that fine-mapping suffers for rare variants especially for JCTF, presumably due to the fact that the genotypes are imputed.

Supplementary Figure 5. Detailed comparison of PIP from two fine-mapping algorithms (SuSiE and FINEMAP)

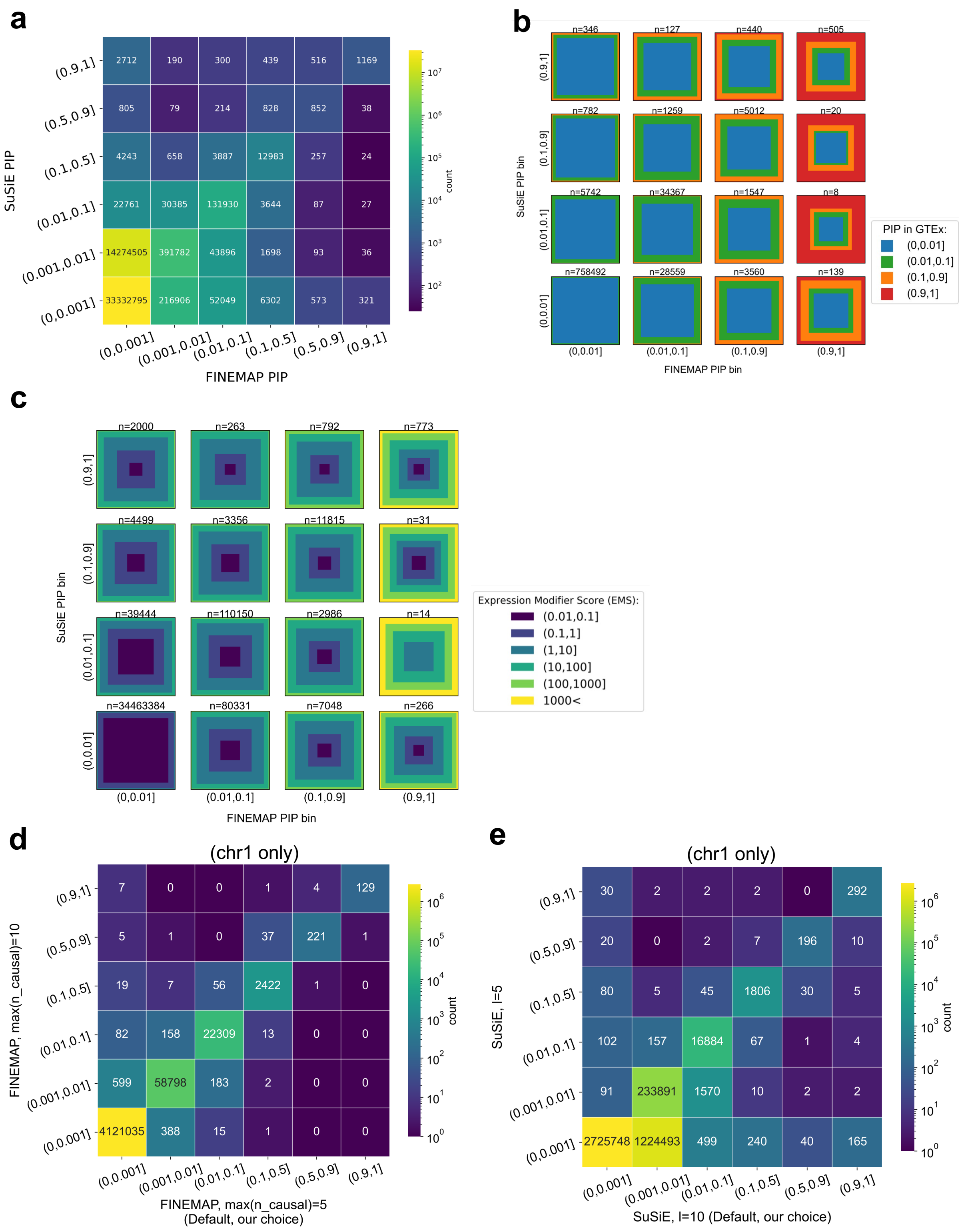

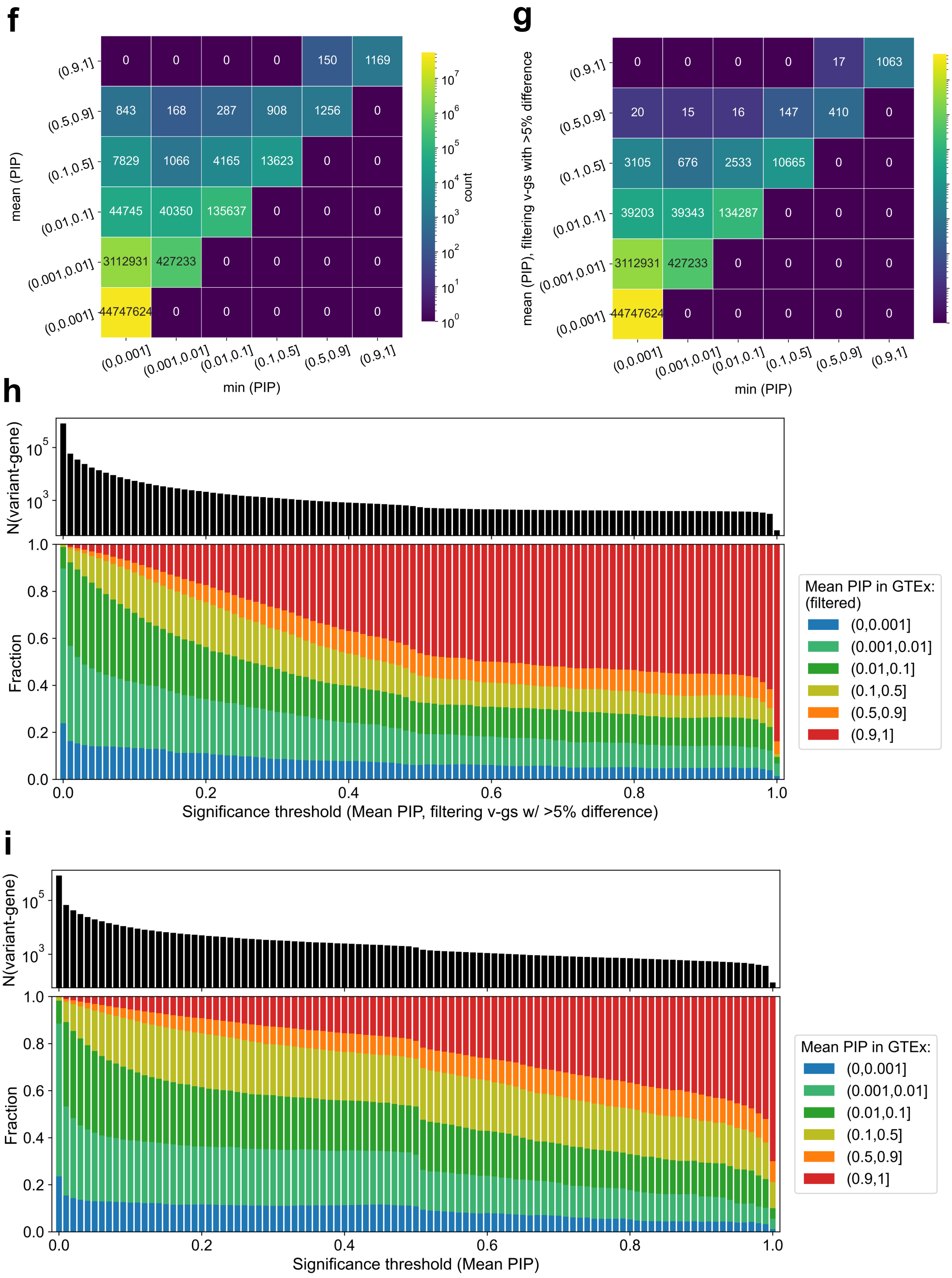

a. Comparison of PIP bin between two methods. **b.** Characterization of variant-genes in each SuSiE and FINEMAP PIP bin by PIP in GTEx (minimum of that from SuSiE and FINEMAP). **c.** Characterization by the Expression Modifier Score (EMS). **b** and **c** were limited to variant-genes existing in GTEx. The comparison suggests possible inflation in PIP from SuSiE compared to that from FINEMAP, motivated us to take the conservative approach of taking the minimum between those two for downstream analyses. **d.e.** Stability of PIP estimation when changing the number of maximum

causal variants in the algorithms (**d.** n=10 for FINEMAP and **e.** l=5 for SuSiE), downsampled to chr1 for computational ease. **f.g.** Comparison of minimum versus mean PIP from two algorithms. **h.i.** Agreement with PIP in GTEx, when using mean instead of minimum PIP. **g.** and **i.** are when filtering the ones with difference of PIP > 0.05, as is done in [3].

Supplementary Figure 6. FDR-based eGene classification

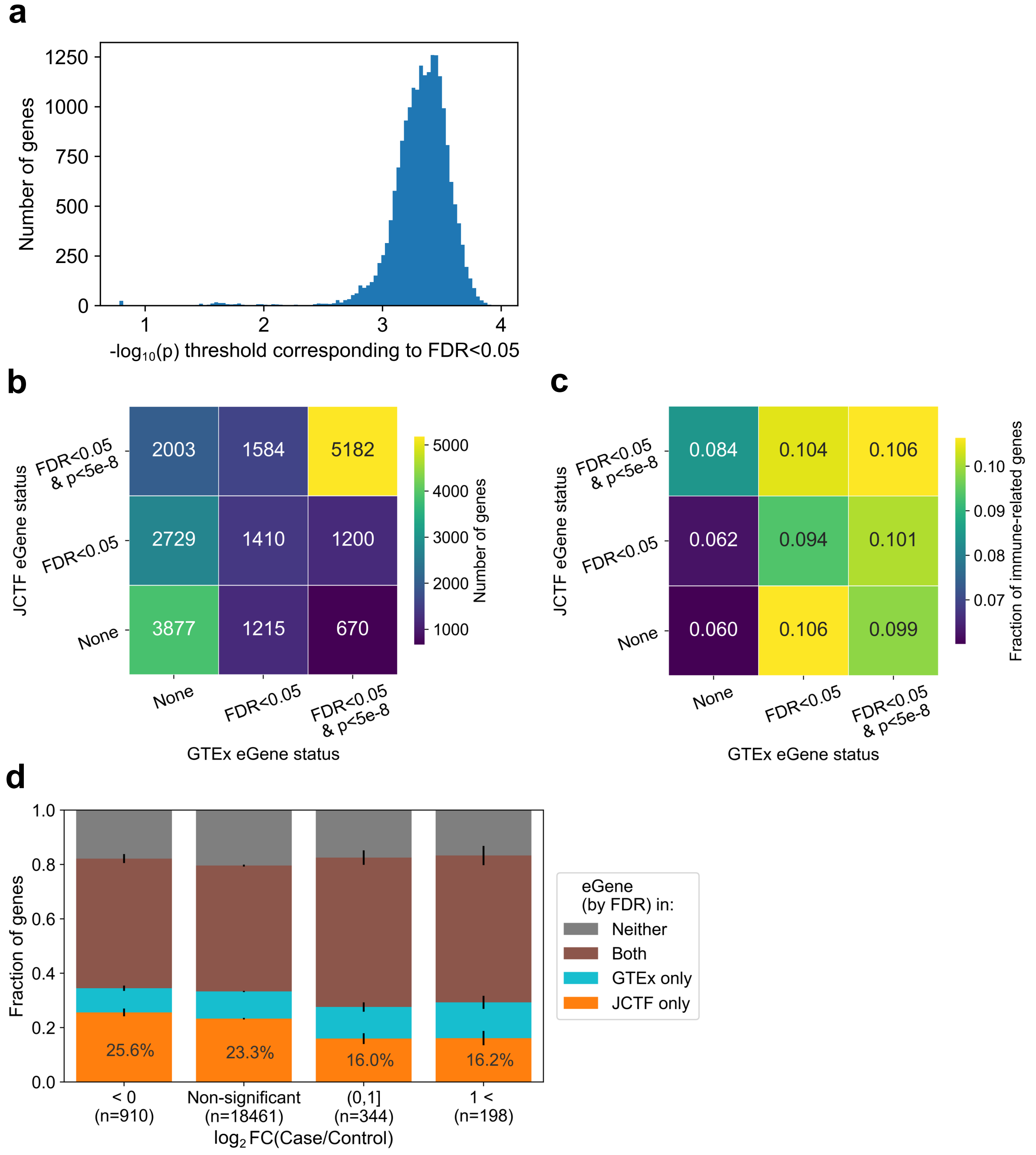

a. Distribution of the corresponding -log$_{10}$(p) threshold when defining eGenes based on FDR<0.05. **b.** The number of eGenes based on strict p<5.0×10^{-8} (5e-8) (=the one used in the main analysis) or more generous FDR<0.05 threshold, for two populations. **c.** Fraction of immune related genes (REAC:R-HSA-168256) for each eGene category. **d**. Fraction of eGenes stratified by the study population and differential expression status, defined FDR<0.05 (instead of p<5e-8 as in **Fig. 7a**). **c.** shows that immune related genes (as a proxy of genes functional in blood) is along with statistical confidence, and **d.** shows decrease of JCTF-specific eGenes for highly differentially expressed genes, consistent with **Fig. 7a**, together validating that using FDR-based eGene call is a reasonable alternative method and will not change the main results of our analysis.

Supplementary Figure 7. Agreement between significance statistics (marginal p-value or posterior inclusion probability=PIP) in our analysis and PIP in GTEx

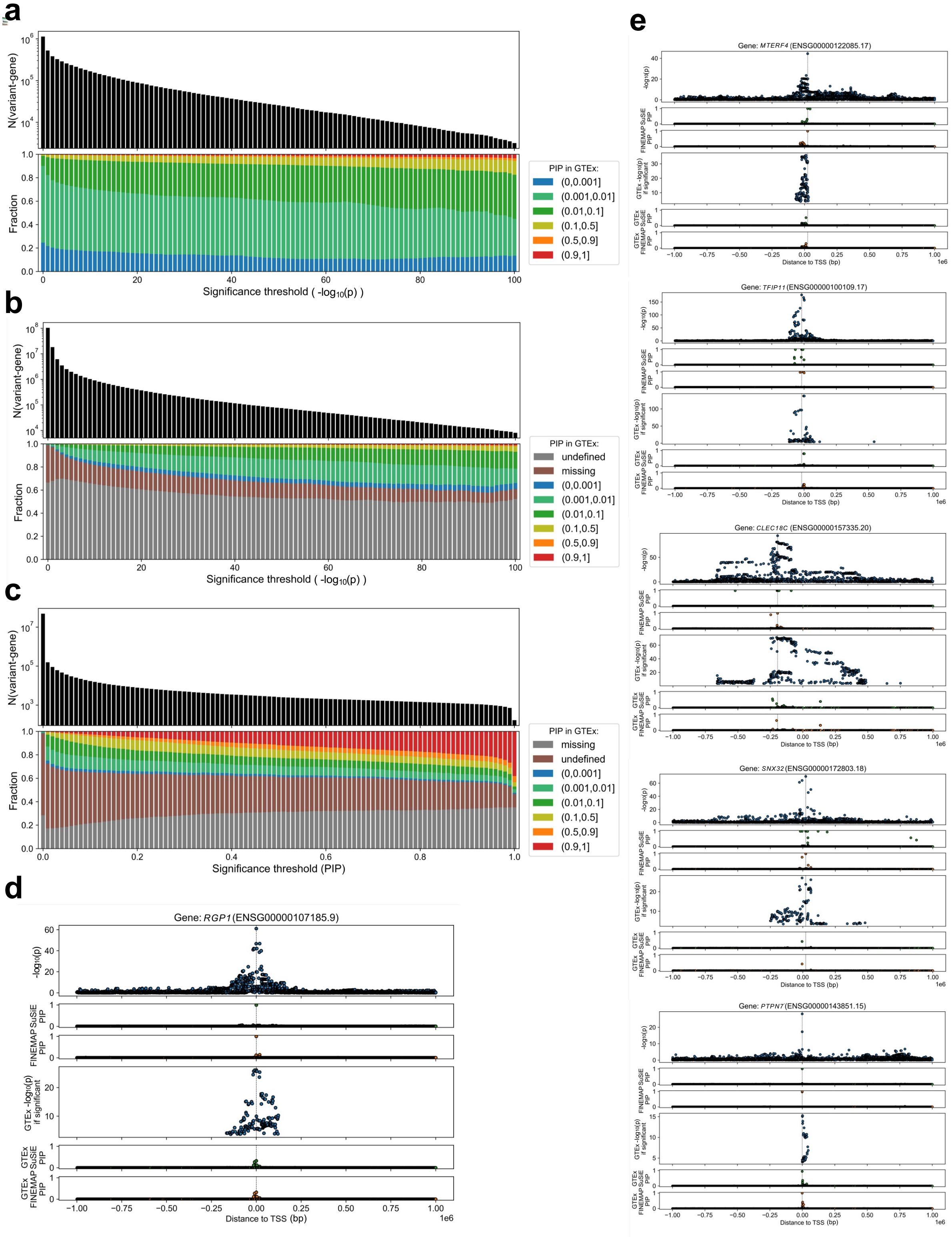

The x axis is the marginal p-value in **(a,b)**, and is PIP in **(c)**. Variant-genes missing in GTEx are included in **(b,c)** for comparison, while those are removed in **(a),** as well as other figures such as **Fig. 2b,d**. The “undefined” category includes non-eGenes in GTEx, as well as v-gs with PIP=0 in both algorithms (the case where we cannot distinguish whether the algorithms failed, or are truly outputting exact 0), and thus

are likely to contain substantial amount of non-causal v-gs. Example of variant-gene harboring PIP>0.9 in JCTF but <0.9 in GTEx presumably simple due to difference in the LD structure is shown in **(d)**. Examples of five variant-genes where PIP is above 0.9 in JCTF but below 0.1 in GTEx across two fine-mapping methods are shown in **(e)**. These examples could be due to further understanding of differences in the true causal variants and/or fine-mapping algorithms' artifacts, and motivates us for further inspection. We did not show LD information in the plot, for visual simplicity.

Supplementary Figure 8. Additional functional characterization of putative causal eQTLs

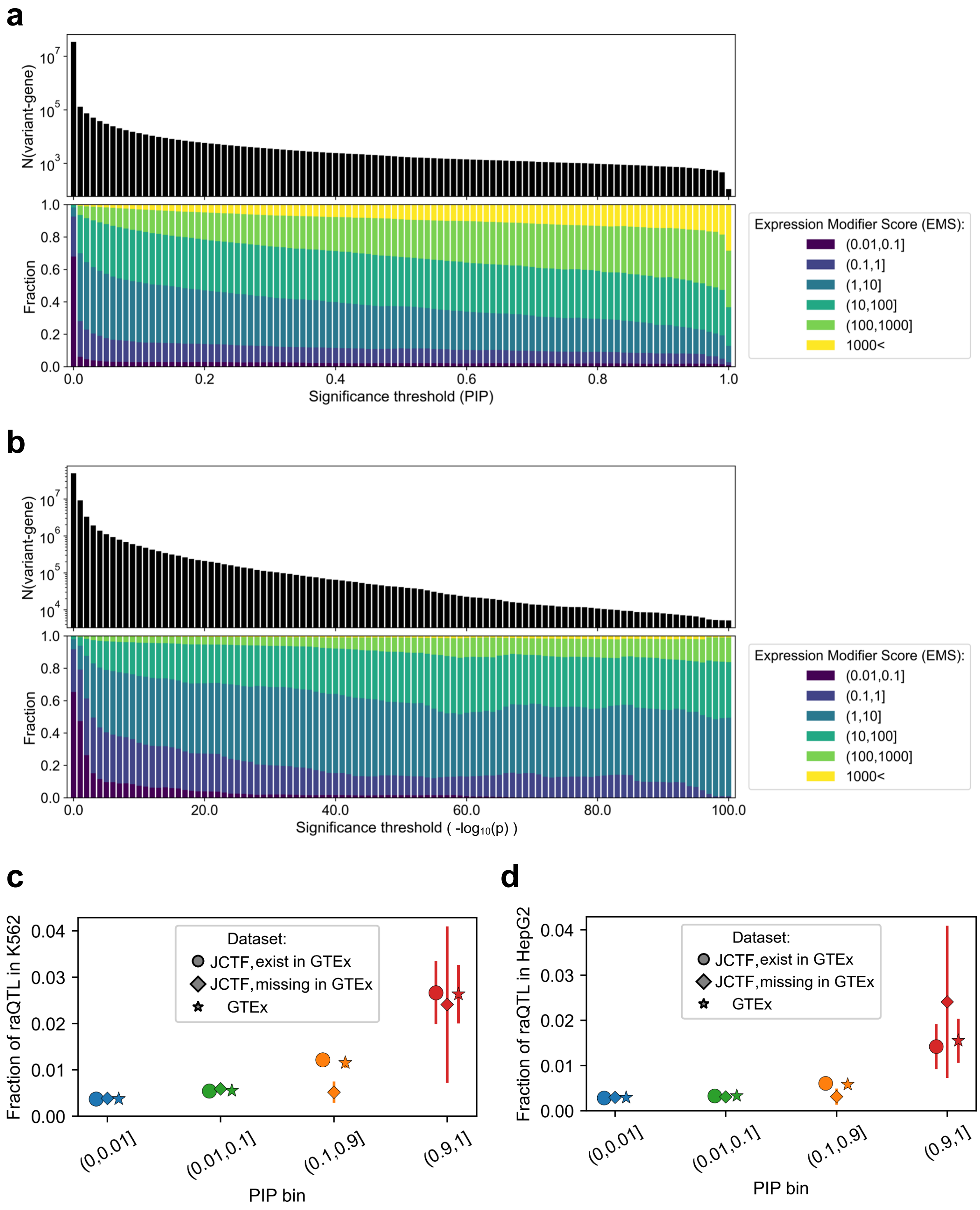

a.b. Distribution of the expression modifier score (EMS) along with the marginal p-value (**a**) or the PIP (**b**) in JCTF, for the variants that exist in GTEx. **c.d.** Enrichment of reporter-assay QTLs (raQTLs) along with the PIP bin in JCTF for the variants existing (circle) or missing (star) in GTEx, as well as that for PIP in GTEx for the ones existing (diamond). **c.** is for K562 and **d.** is for HepG2. n=25611365, 91358, 10315 and 646 GTEx variant-genes for each bin.

Supplementary Figure 9. Agreement with a study from African American cohort (MESA)

a

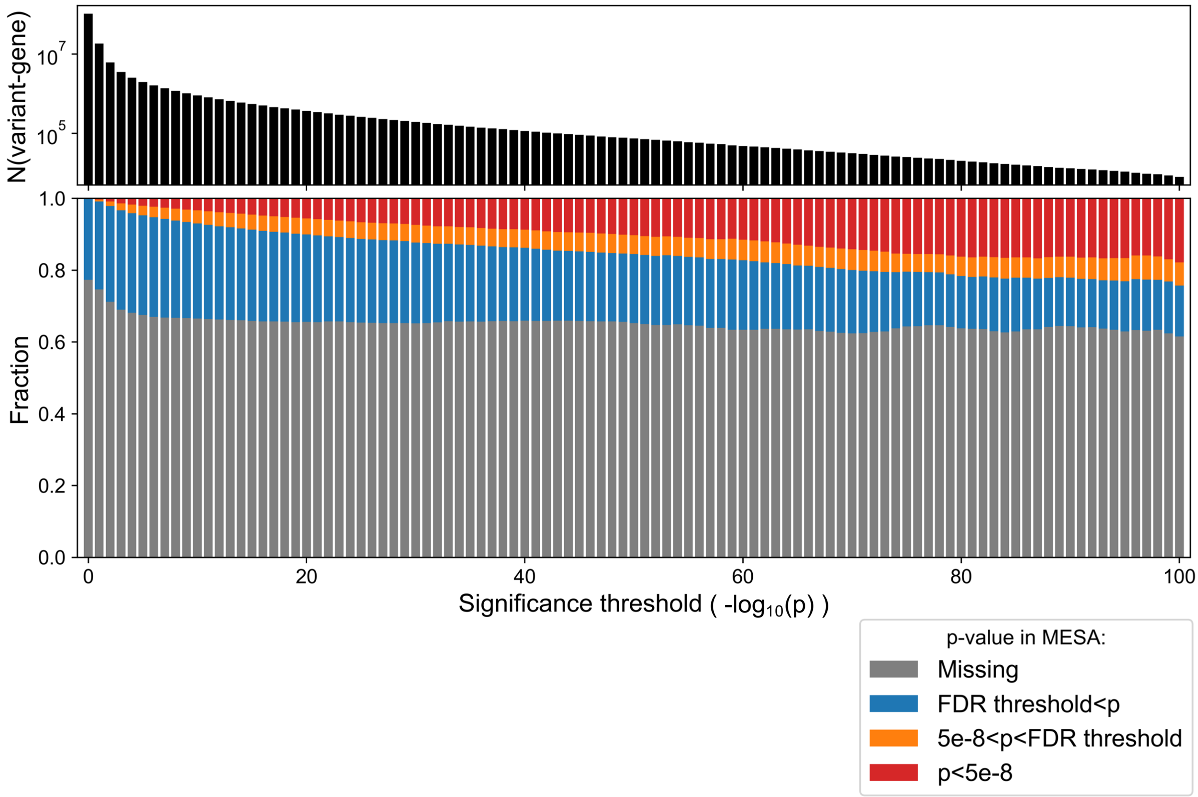

b

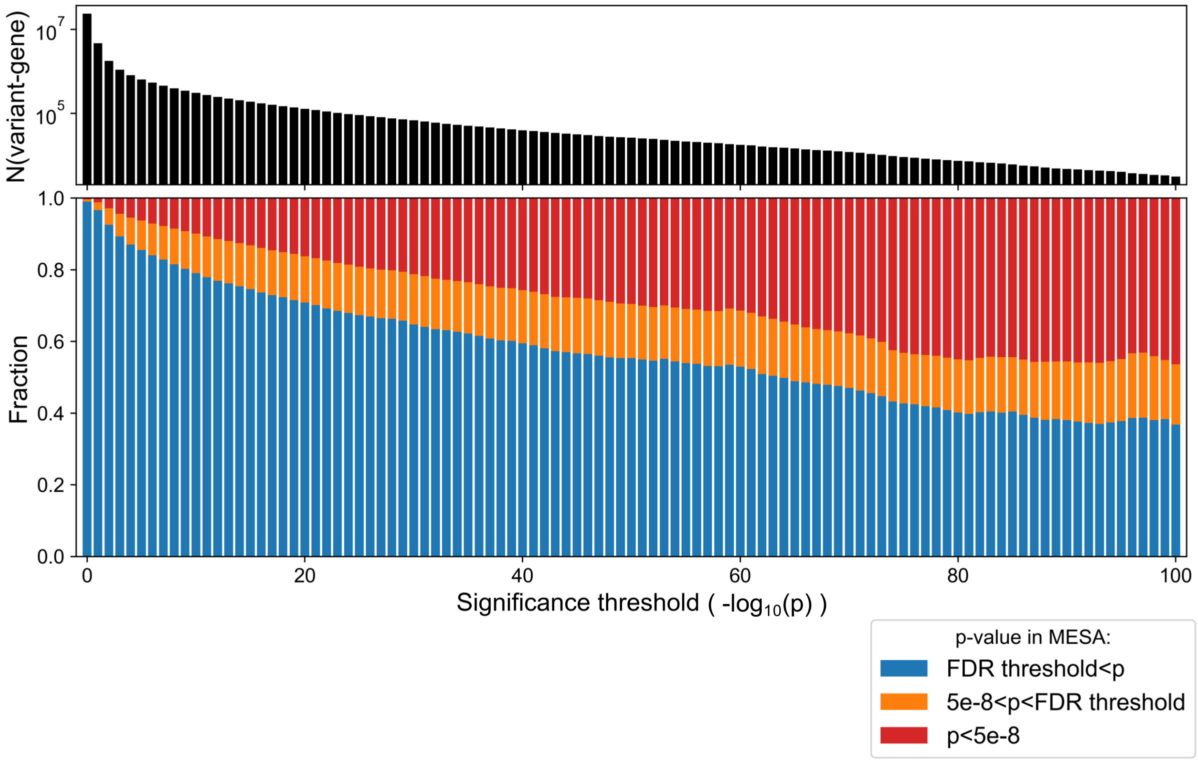

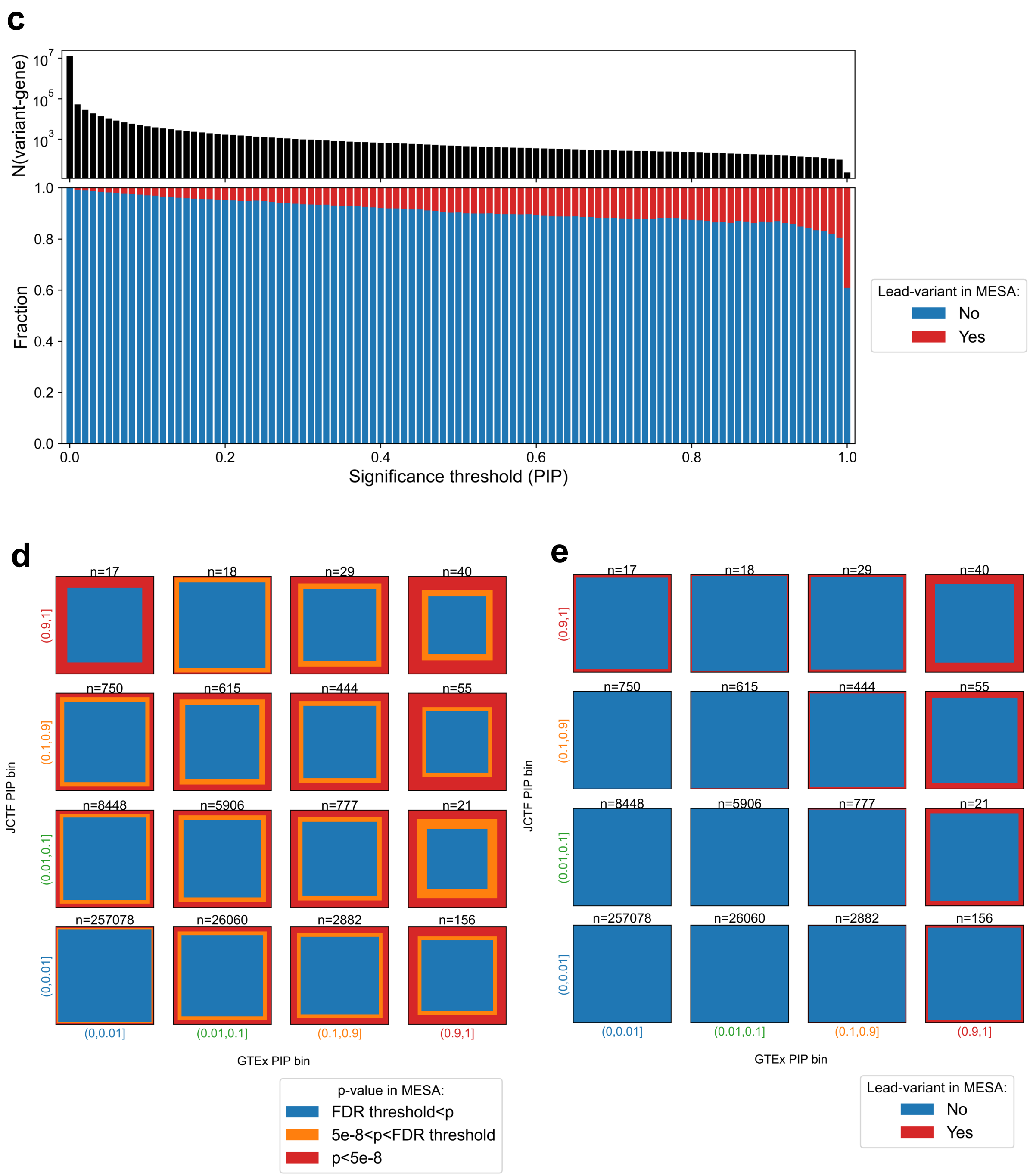

a. b. The number and the fraction of variant-genes classified into different p-value bins in MESA study[22], including **(a)** or excluding **(b)** the variants missing in MESA. **c.** is using whether a variant-gene is a lead variant or not, as a proxy of "causal" eQTL (i.e. we assume that the lead variant, which means the variant has the lowest p-value for the gene, is highly enriched for causal eQTLs). **d. e.** Distribution of significance bins in MESA (**d**:p-value, **e**: lead-variant) for different PIP bins in our study (y-axis) and GTEx (x-axis). The fraction is represented as the area in each bin. Variant-genes with p<5e-8 as well as lead variant-genes are most enriched for top-right bin (i.e. when 0.9<PIP in both JCTF and GTEx). 5e-8 = 5.0×10^{-8}.

Supplementary Figure 10. Detailed inspection of *TORAIP1* gene in the context of sQTL fine-mapping

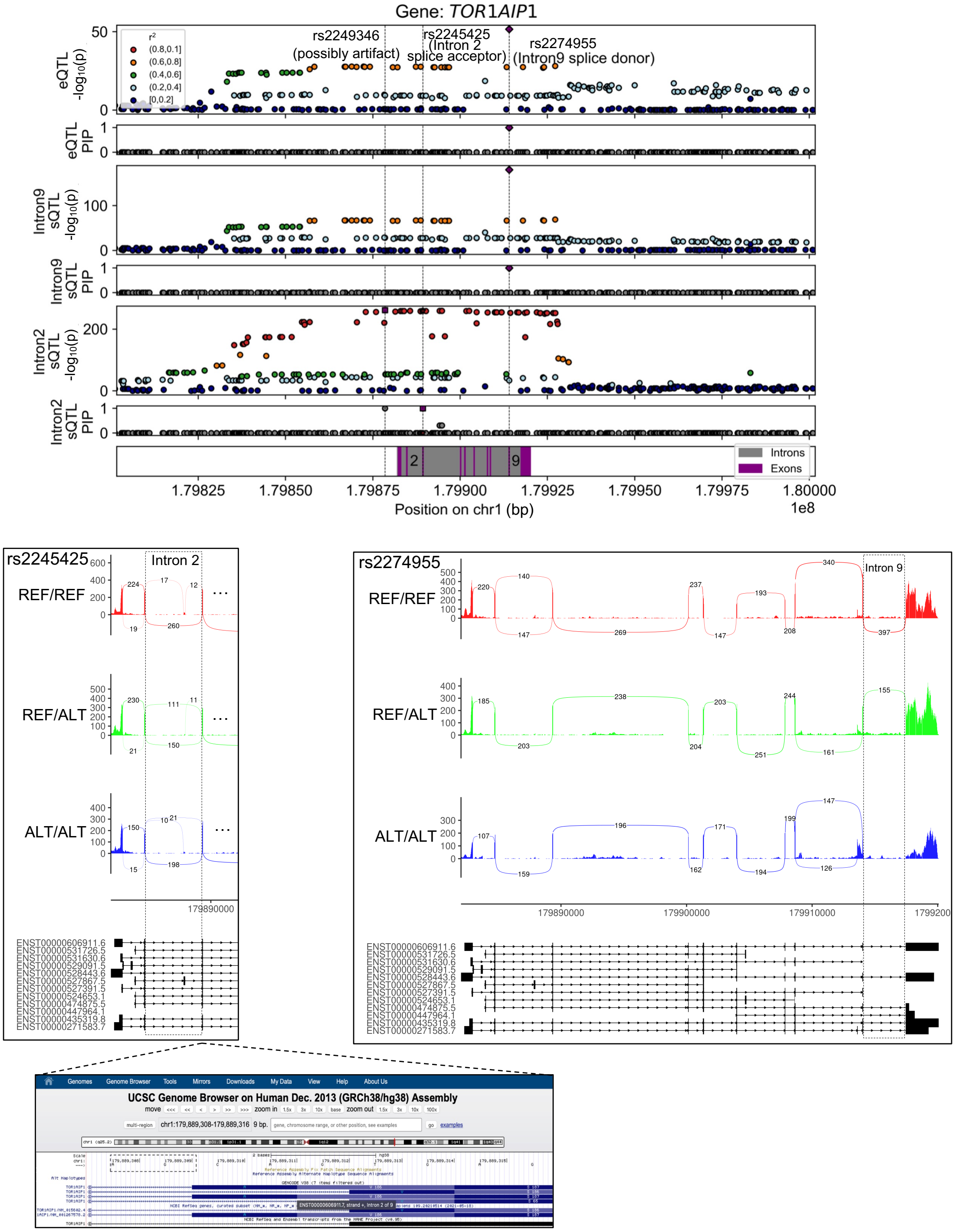

The PIP for each signal is shown in addition to the plots in **Fig. 3f,g**. The putative causal sQTL for Intron 2 splicing (rs2249346) is presumably mis-identified as the one upstream of the gene body, suggesting possible false positives in sQTL call, as well as the importance of incorporating functional annotations in fine-mapping. Detailed inspections of the splice pattern in sashimi plots, as well as the UCSC genome browser screenshot for the proximal splice sites are provided in the lower panels. We downsampled to three samples for each genotype and set the cutoff to be 10 reads for intron 2 and 100 for intron 9 when visualizing in ggsashimi.

Supplementary Figure 11. Landscape of colocalization between whole blood eQTL and hematopoietic traits in Biobank Japan

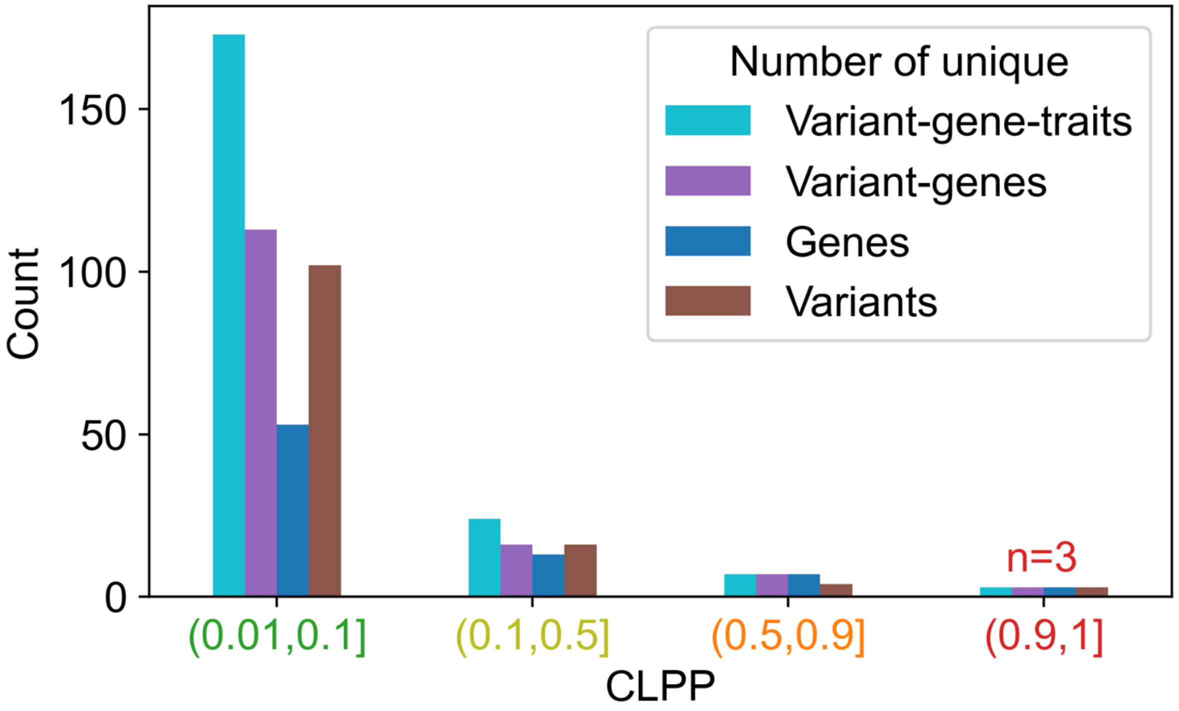

The number of unique entries in different scales (e.g. per variant or per variant-gene-trait) are shown in different colors.

Supplementary Figure 12. Fine-mapping the eQTLs for genes with possible association with COVID-19 susceptibility or severity

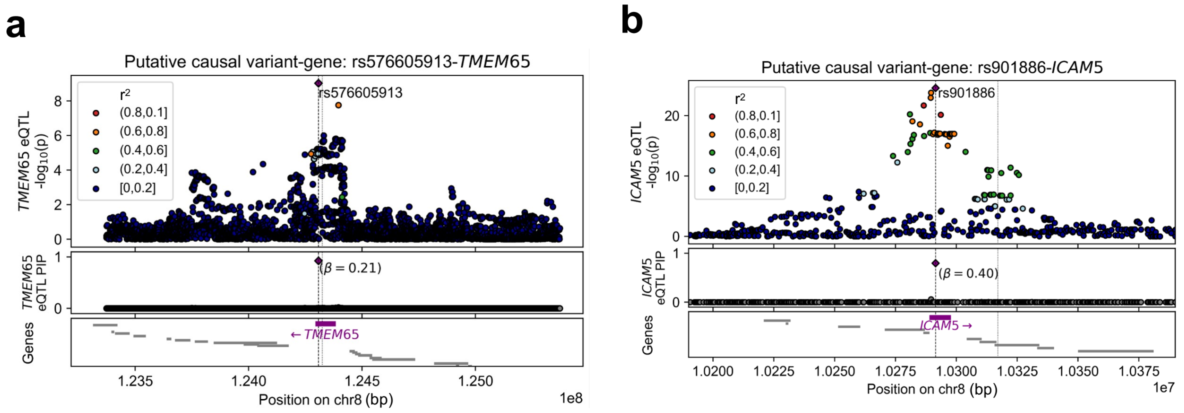

Association p-value (top), eQTL PIP (second row) for the SNVs in +-100 kb of the putative causal variant, and the location of the genes (bottom row). The putative causal variants and genes are colored with purple.

Supplementary Figure 13. Trans-eQTL z-score scatter plot accounting for multiple variant and gene

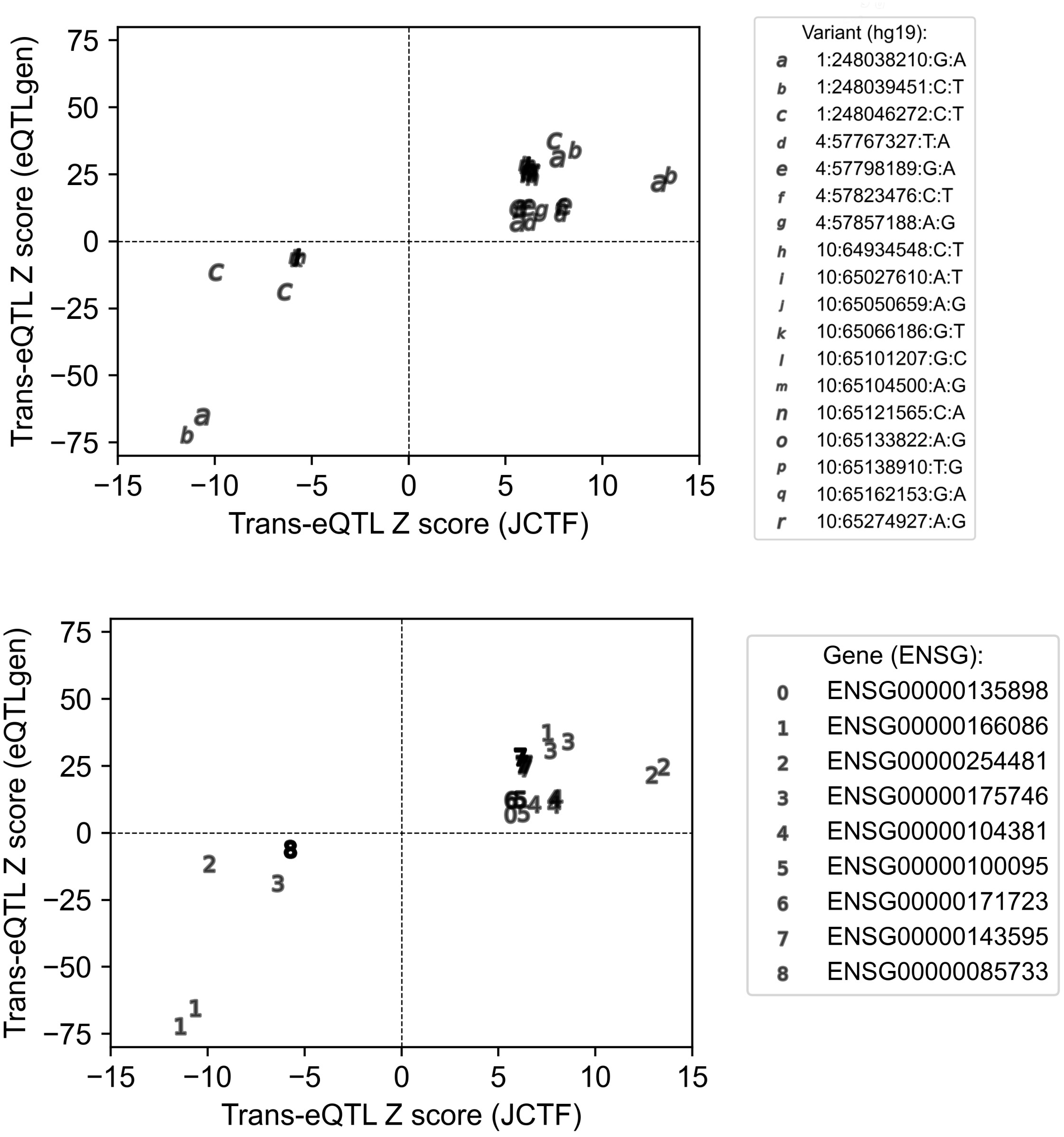

Scatter plot showing the trans-eQTL effect sizes (z-score) in our analysis (x-axis) and in eQTLgen (y-axis) for the 37 variant-genes identified as trans-eQTL both in two analyses (data identical as **Fig. 5a**), but plotting each variant (top) or gene (bottom) with a unique marker. These plots together show that the correlation is not driven by single variant tagging all the others.

Supplementary Figure 14. Increasing number of overall agreement with ImmuNexUT along with p-value or Posterior inclusion probability (PIP) in our analysis

a

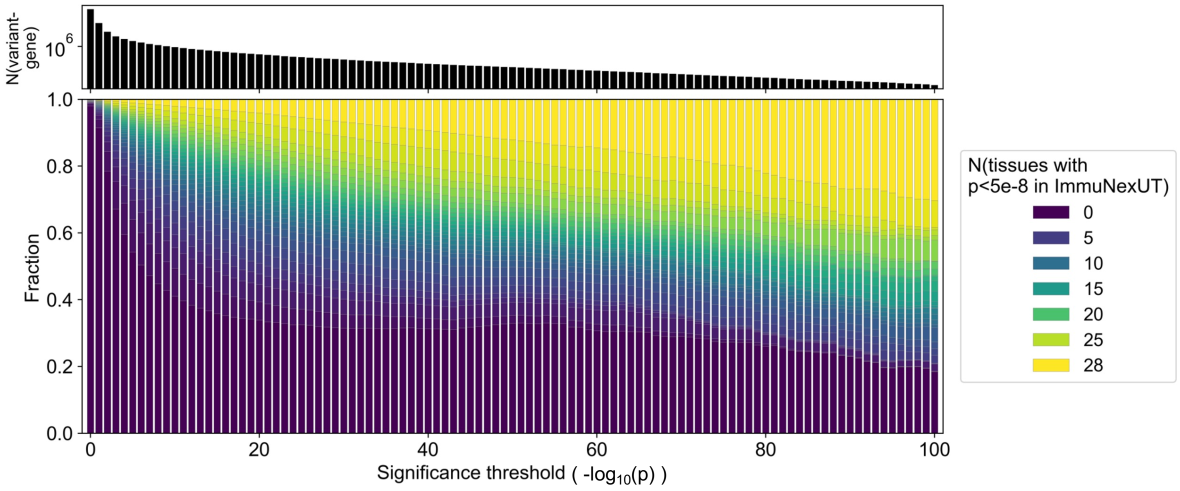

b

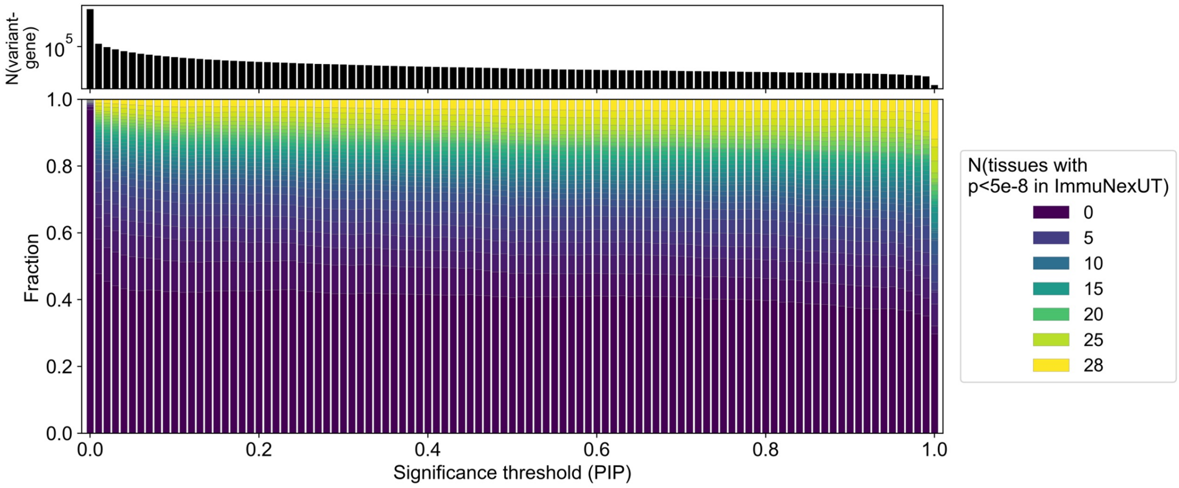

c

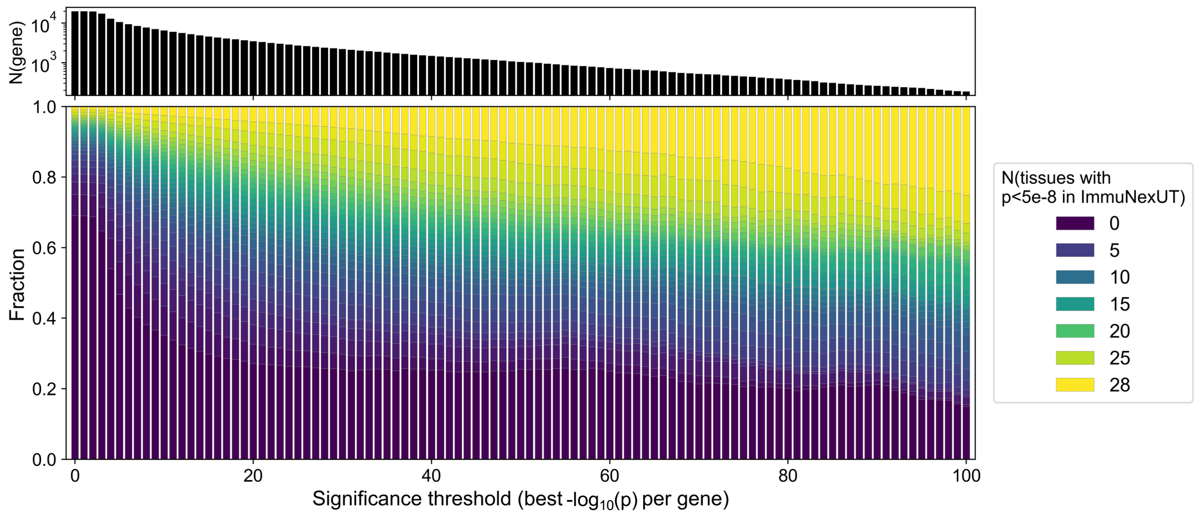

d

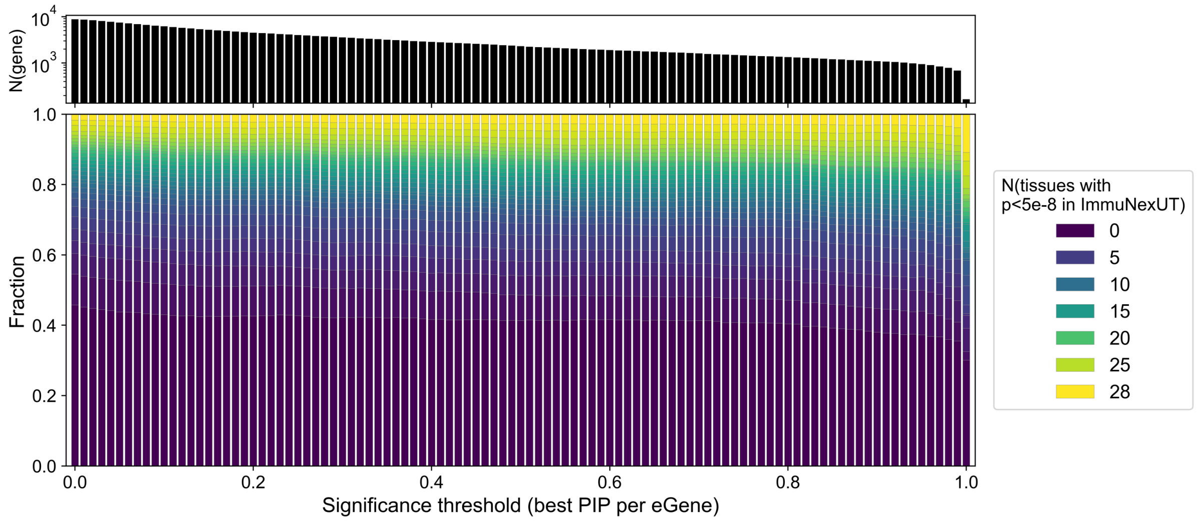

The figures show that higher significance (p-value or PIP) in our JCTF whole blood eQTL call corresponds to significant eQTL effect in larger number of cells in ImmuNexUT, consistent with previous literatures showing eQTLs with larger effect sizes tend to have effects across multiple tissues [23]. (**a**) and (**b**) are for every variant-gene pair, while (**c**) and (**d**) are for top variant-gene pair, both yielding roughly consistent results. The increase of significant cell types in ImmuNexUT along with x axis is observed for both marginal p-value and PIP, and is more prominent for p-value compared to PIP, as expected simply because the ImmuNexUT data is not fine-mapped and includes a large number of tagged non-causal variants. 5e-8 = 5.0×10^{-8}. Same discussion is applied for **Fig. S15**.

Supplementary Figure 15. Increasing number of agreement with ImmuNexUT along with p-value in our analysis, per cell type

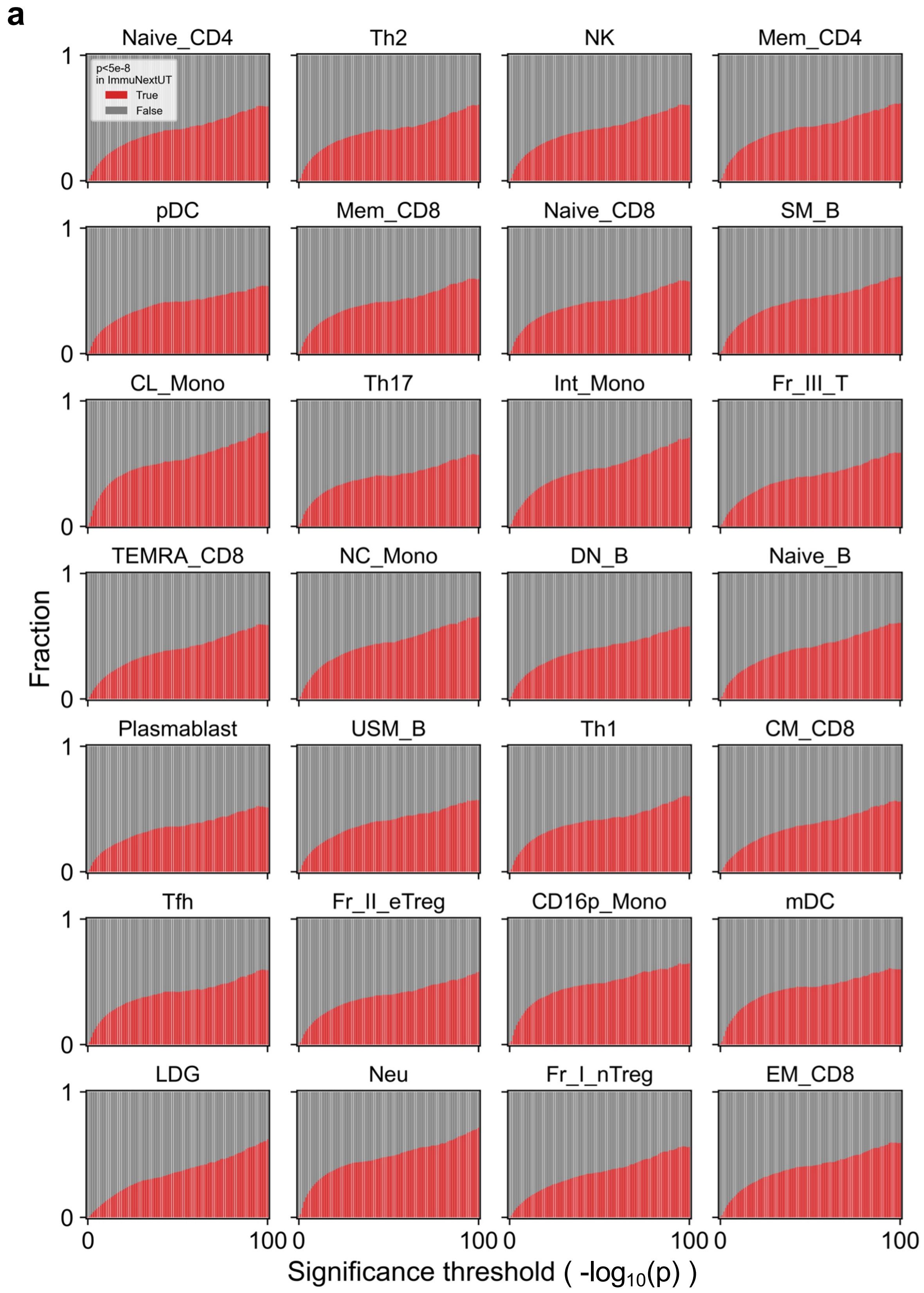

b

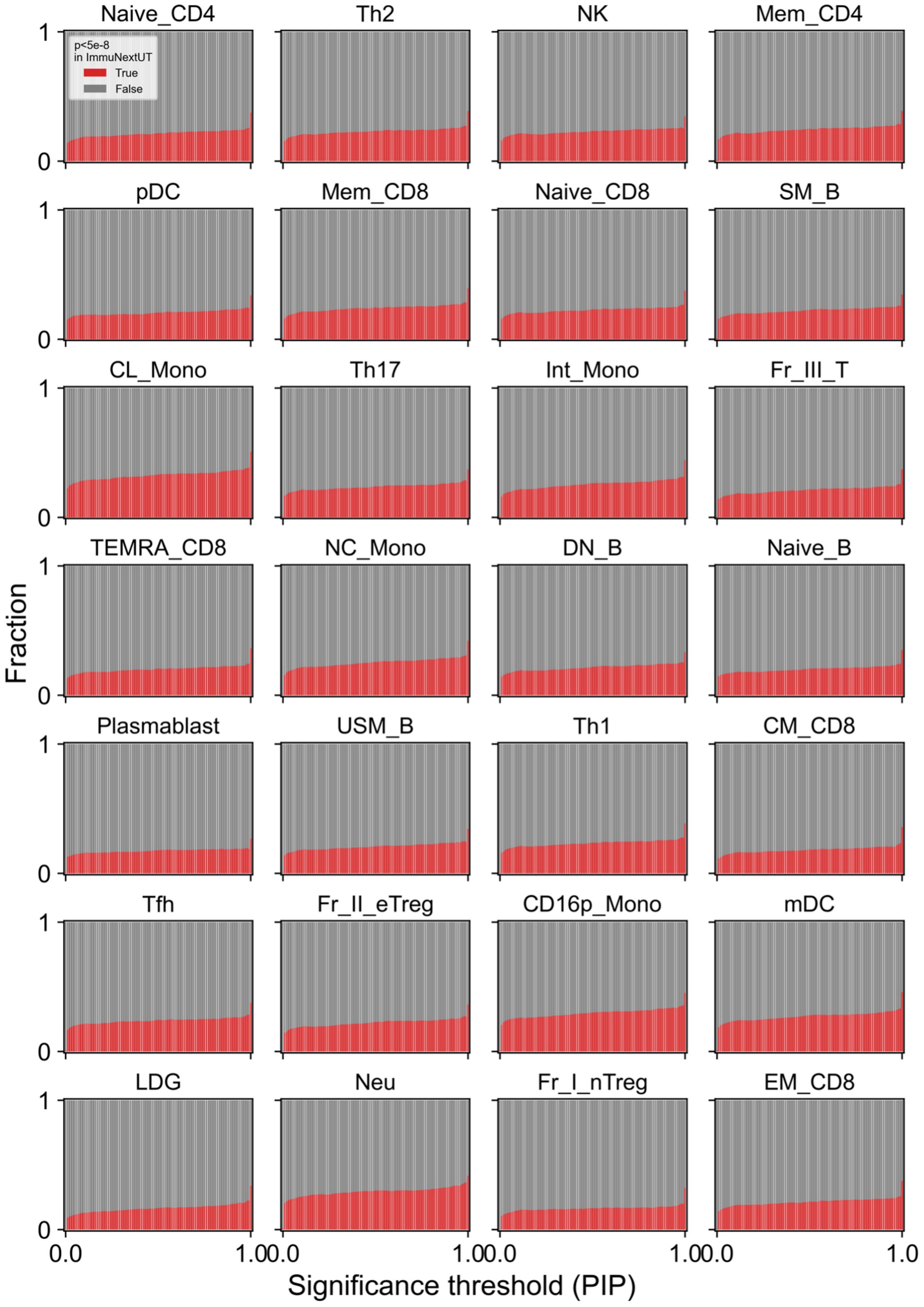

The x axis is the marginal p-value in **(a)**, and is PIP in **(b)**.

Supplementary Figure 16. Differential expression results remain significant after adjusting for inferred cell type composition

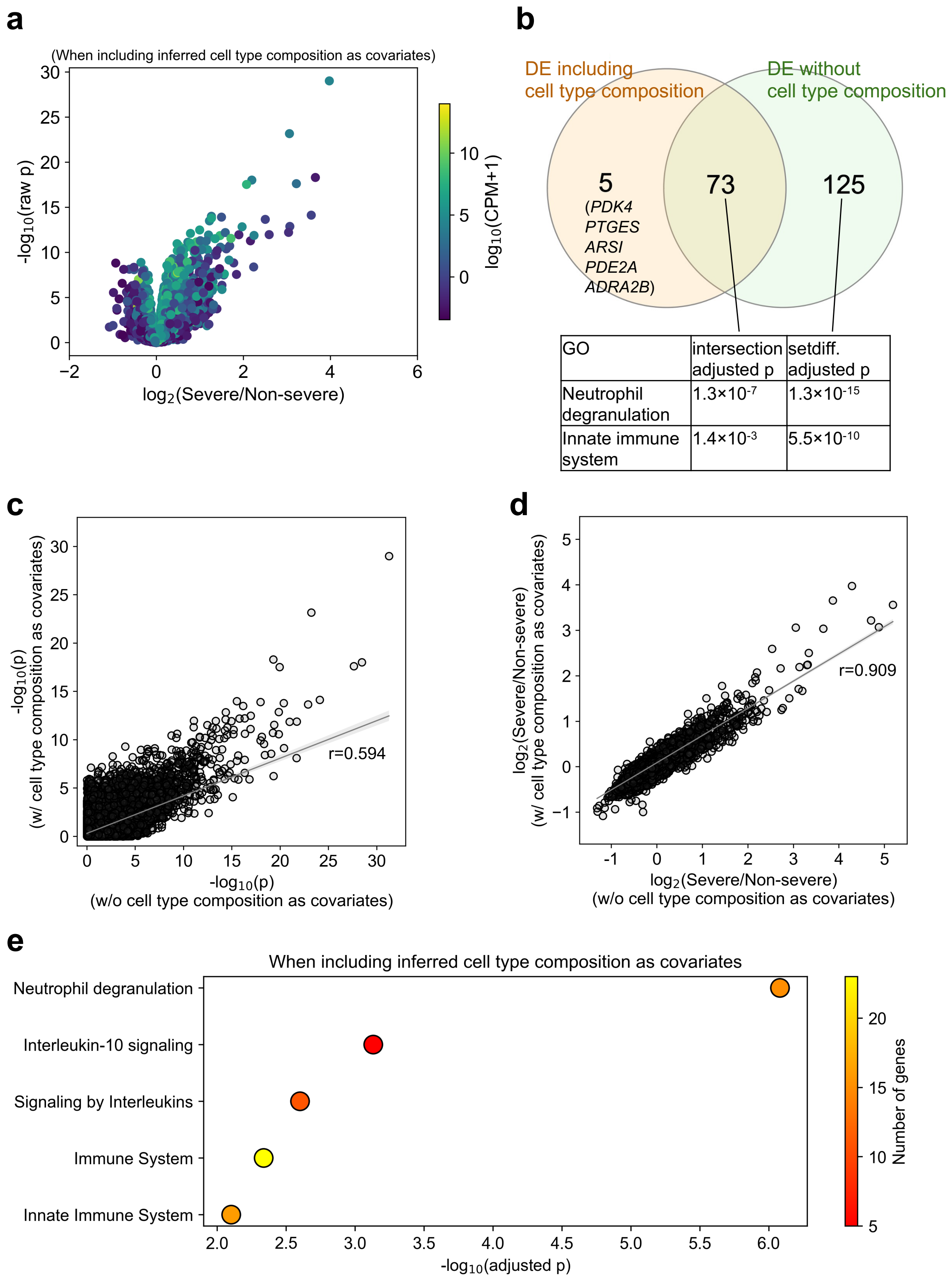

f

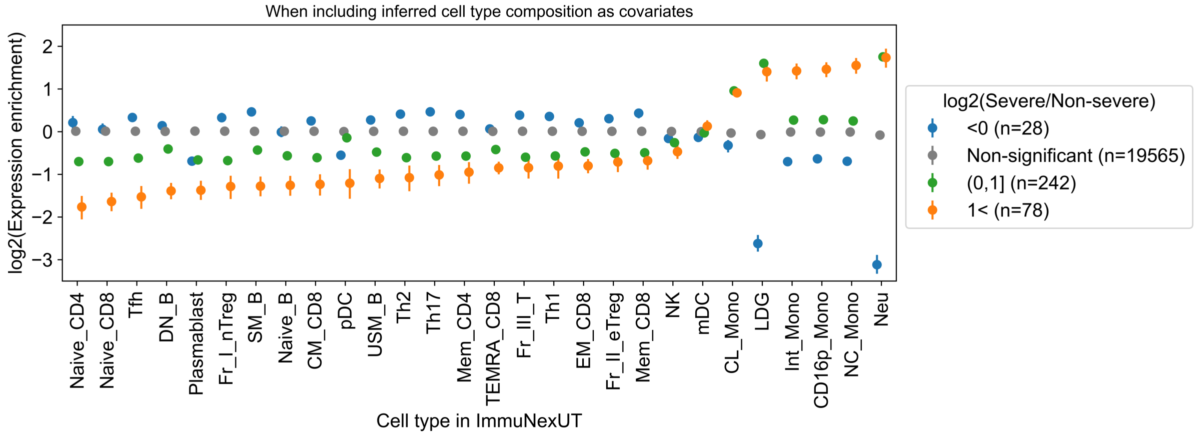

a. Volcano plot showing the difference of the RNA expression level between severe and non-severe COVID-19 cases (x axis, $\log_2$(severe/non-severe)), and the statistical significance (likelihood ratio test p-value, y axis). Color shows the $\log_{10}$(count per million + 1), when including inferred cell type composition. **b.** Venn diagram showing the number of expression-increased genes that remain (or do not remain) significant (p<0.05/#genes, 2<fold change) when including inferred cell type composition, as well as the GO term enrichments. **c.** Scatter plot comparing $-\log_{10}(p)$ of differential expression analysis, before (x axis) or after (y axis) including inferred cell type composition. **d.** Same scatter plot for expression fold change. **e.** GO term enrichment and **f.** Cell type specific expression when including inferred cell type composition. Out of 22 inferred cell type compositions, four cell types highly correlated with COVID-19 severity (|r|>0.25; Naïve B cell, CD8+ T cells, non-activated (M0) macrophages and neutrophils) were included as additional covariates. InteractiVenn web portal (http://www.interactivenn.net) was used for **b**.

Supplementary Figure 17. Differentially spliced genes in severe COVID-19 cases

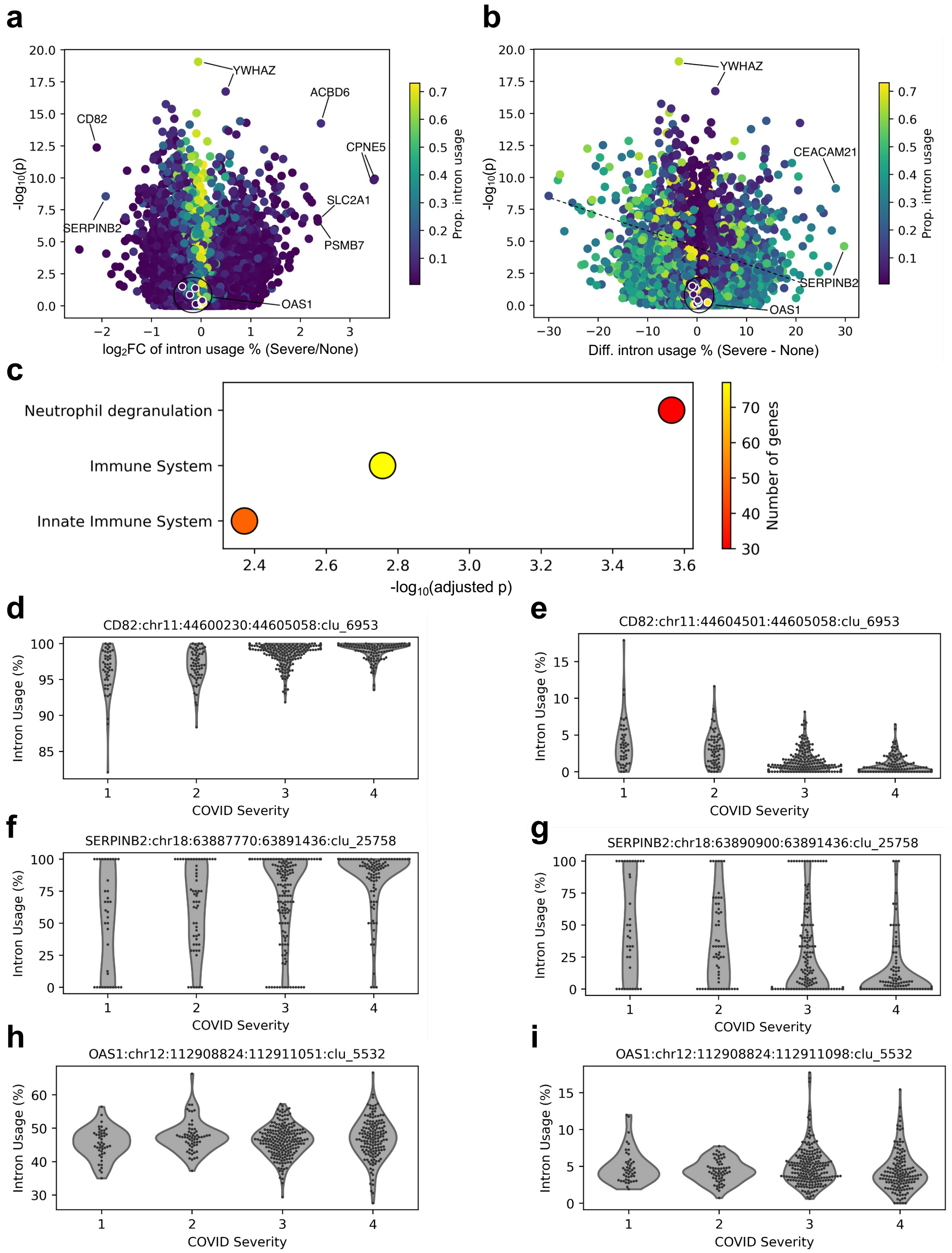

a.b. Volcano plot showing the difference of the fraction of intron used between severe and non-severe COVID-19 cases (**a.** $\log_2$(severe / non-severe) and **b.** % in severe - % in non-severe). y axis shows the p-value in log likelihood ratio test (LRT) (thus common for **a** and **b**). **c.** GO term enrichment of significantly-enriched ($p<0.05$ / #tests) genes in severe cases (n=598). **d-i.** Swarm and violin plot showing the intron

usage (y axis) along with the COVID-19 severity (x axis), for three example genes and two intron regions each. $p<10^{-10}$ for **d-g**, and p=0.03 for **h** and **i** in LRT. Swarm plot does not necessarily show all the data points, when the distribution is skewed (e.g. large number of zeros). While we observed a decrease in the intron usage of *OAS1* (p=0.03), the position is different from previously reported. For *ACE2*, another known gene with a suggested link between its splice defect and COVID-19 disease phenotype, RNA-seq in whole blood is underpowered to examine its splicing landscape due to low baseline expression level.

Supplementary Figure 18. Correlation between the median gene expression in different sample groups

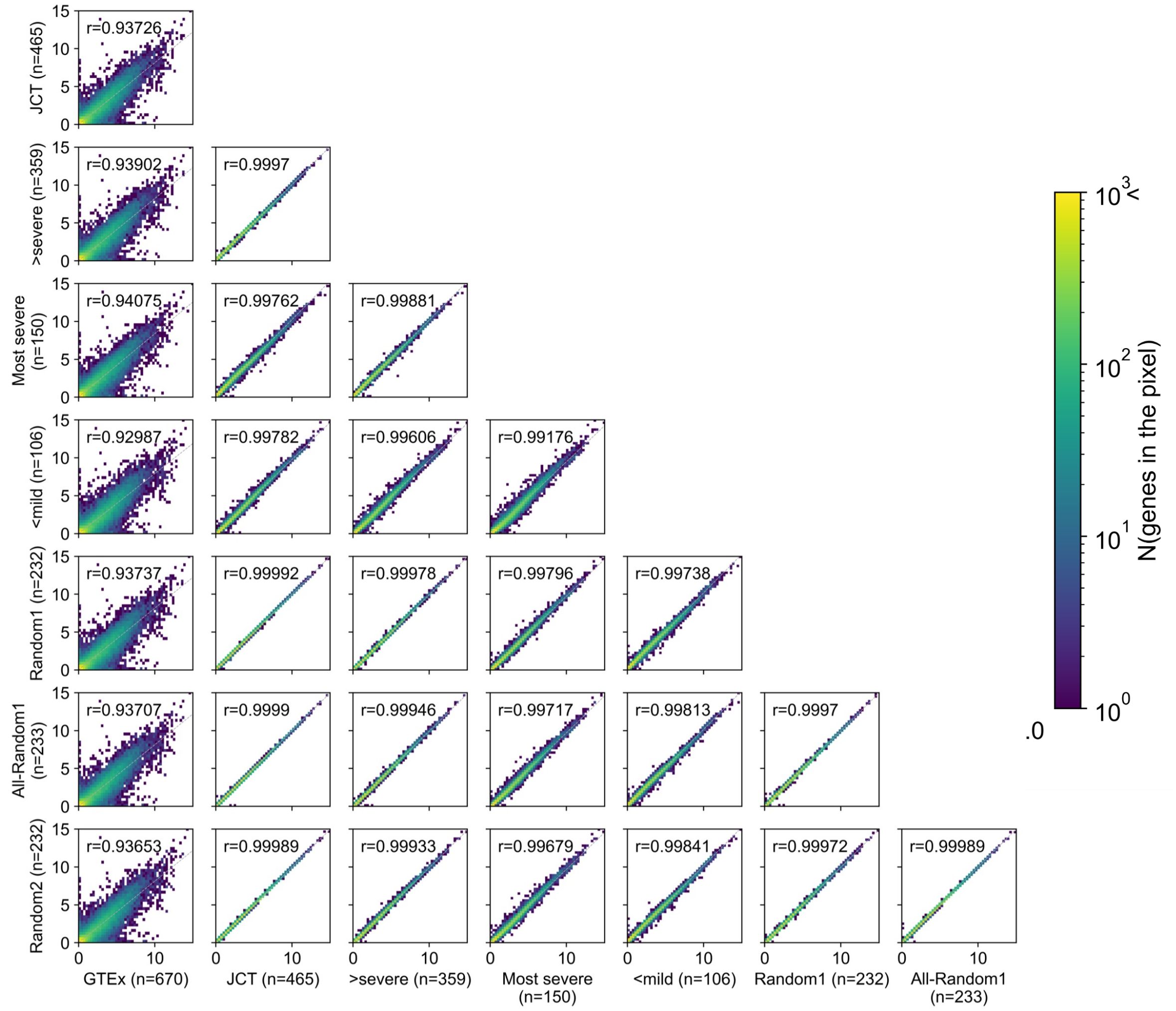

Median TPM was calculated and plotted for each pair of sample groups. Random 1 and Random 2 are random sampling using seed numbers of 1 or 2.

Supplementary Figure 19. Neutrophil enrichment score-interaction eQTL in GTEx

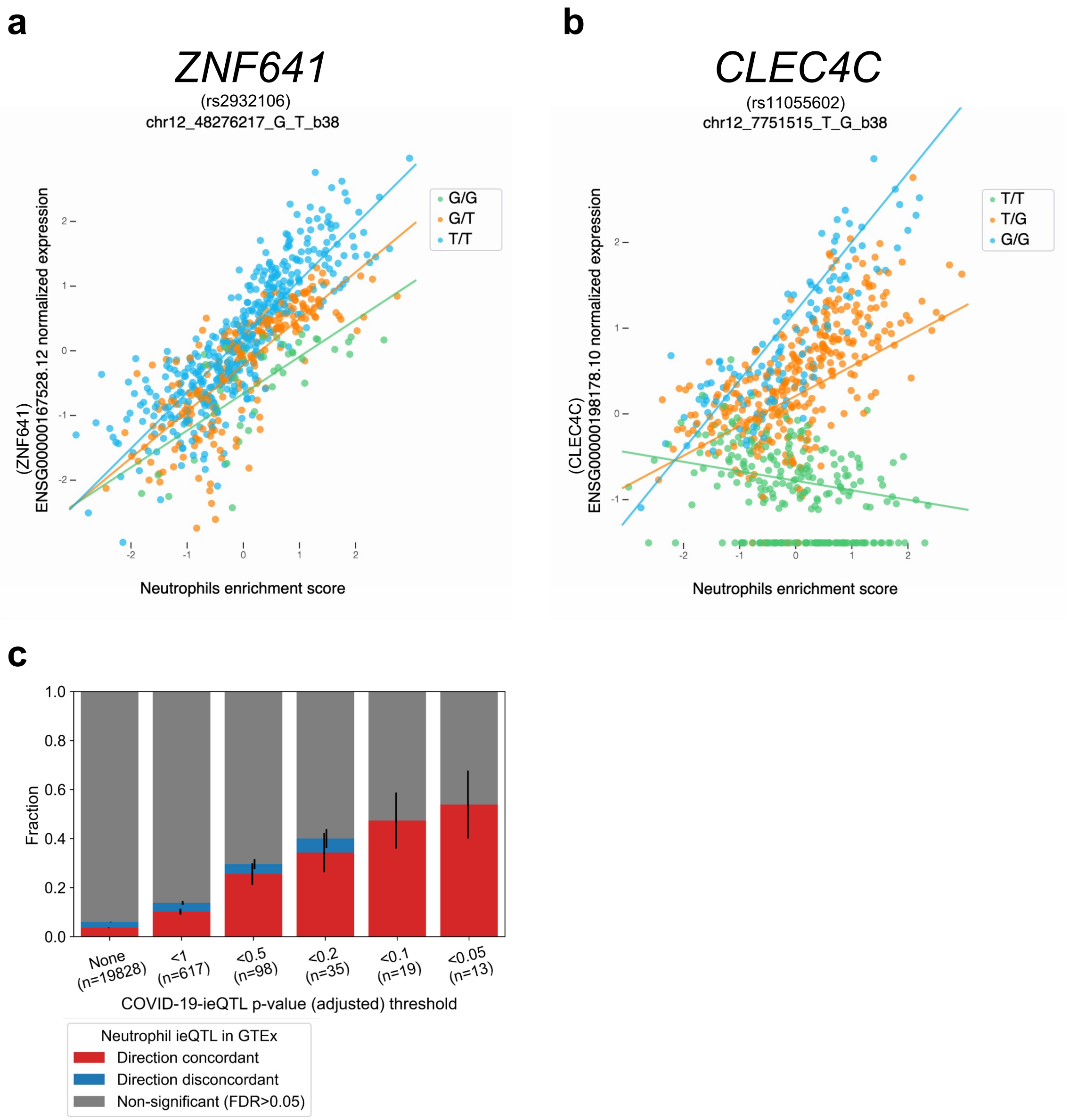

Scatter plots for *ZNF641* (**a**) and *CLEC4C* (**b**) were obtained through the GTEx portal (http://gtexportal.org/). We added canonical gene name (on the y-axis) and rsid (on the title) to the original plot for readability. **c**. The fraction of COVID-19-ieQTLs replicated as GTEx neutrophil enrichment score -ieQTLs, as a function of adjusted p-value threshold. Error bar in **c** is the standard error of the mean of the bottom bar. We used custom method to infer the direction concordance even when the lead variant in GTEx was different from that in our JCTF, as described in **Supplementary Note**.

Supplementary Figure 20. Examples of cell type specific expression for genes with COVID-19-interaction eQTLs

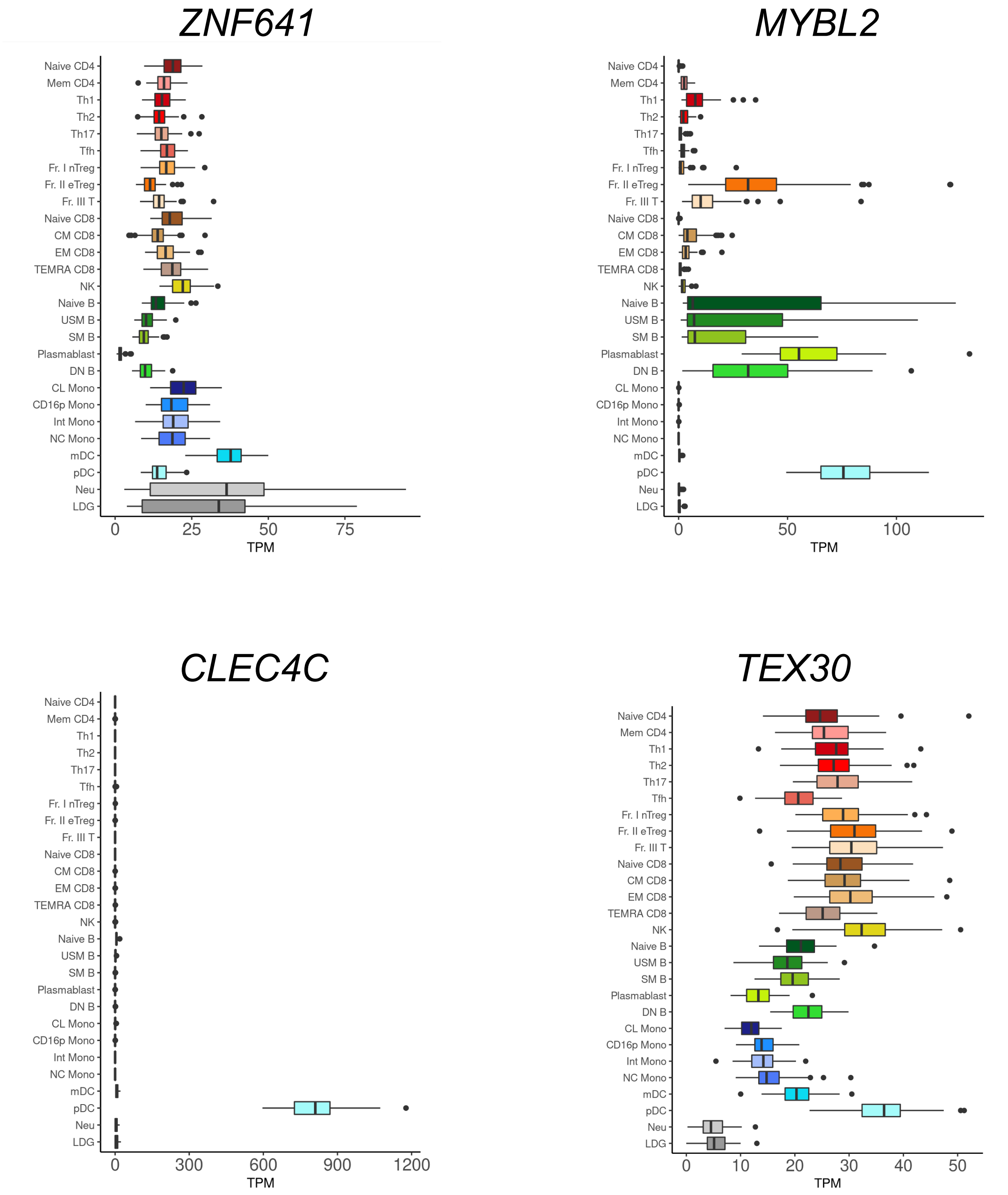

Box plots for *ZNF641* (**upper left)**, *MYBL2* **(upper right)**, *CLEC4C* (**lower left)** and *TEX30* **(lower right)** were obtained through the ImmuNexUT portal (http://immunexut.org/).

Supplementary Figure 21. Fine-mapping and colocalization of eQTLs and ieQTLs

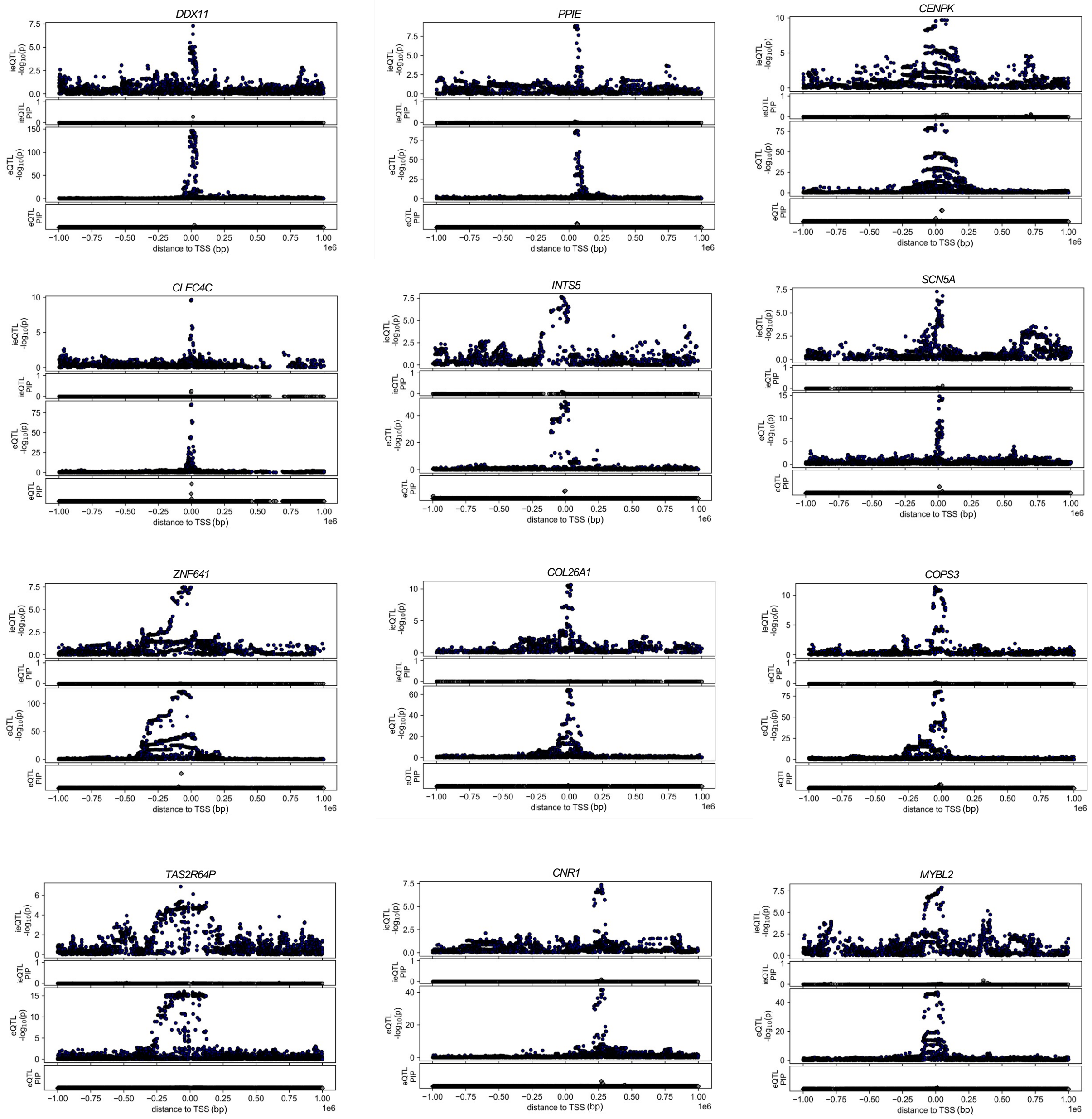

Association p-value (-log$_{10}$, raw) and posterior inclusion probability (PIP) of eQTLs and COVID-19 interaction-eQTL (ieQTL) for 12 genes with adjusted p-value <0.05. For these 12 genes, the peak locations were roughly the same between the eQTL and ieQTL signals.

Supplementary Figure 22. eQTLs and ieQTLs signals for the *TEX30* gene

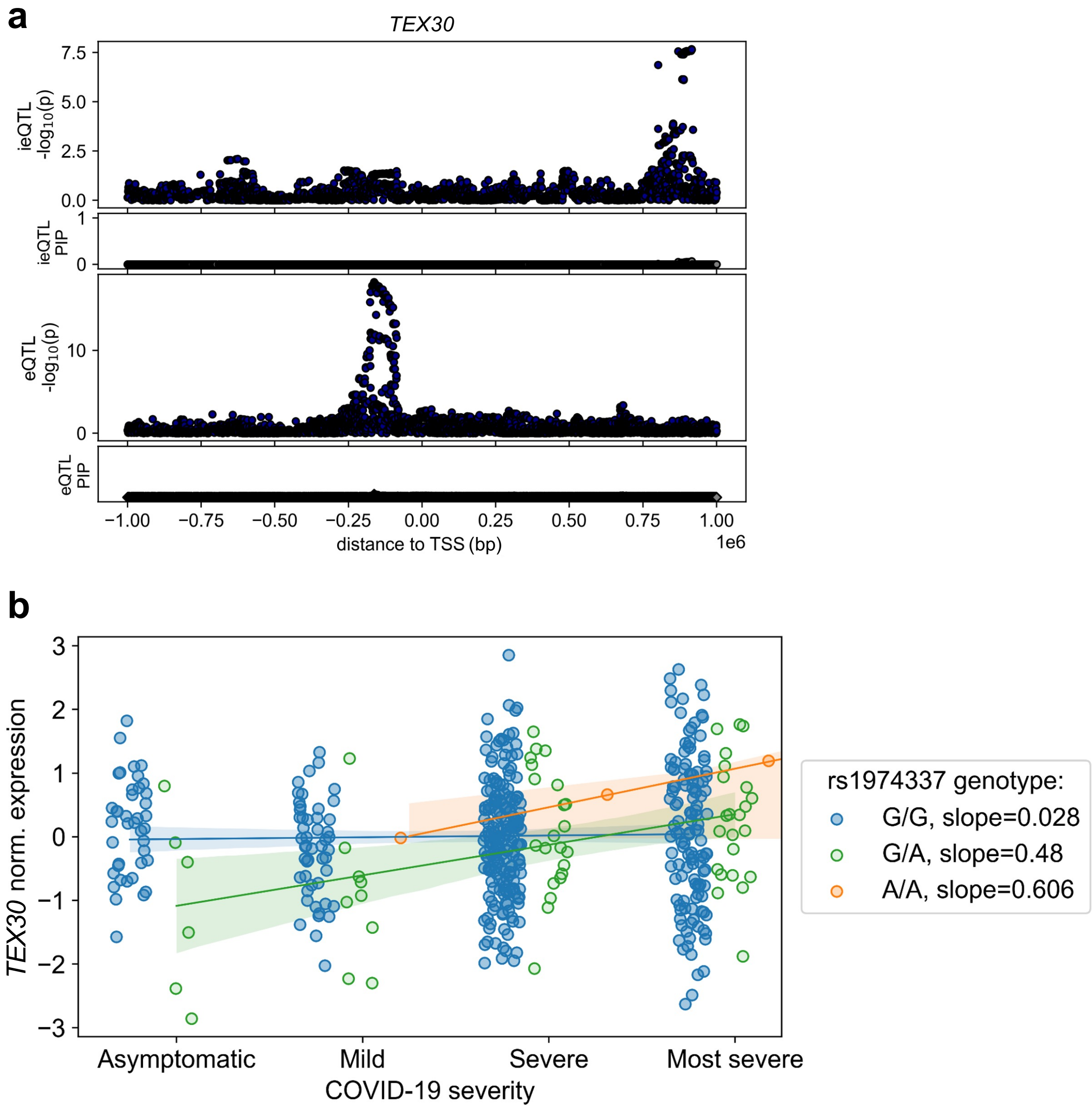

a. Association p-value (-$\log_{10}$, raw) and posterior inclusion probability (PIP) of eQTLs and COVID-19 interaction-eQTL (ieQTL) for *TEX30* gene, clearly presenting distinct peaks. **b.** Scatter plot of the normalized expression of *TEX30* gene stratified by genotype (color) and COVID-19 severity (position). Error band denotes the 95% confidence interval.

Supplementary Figure 23. COVID-19 ieQTL call results before and after adjustment for inferred cell type composition

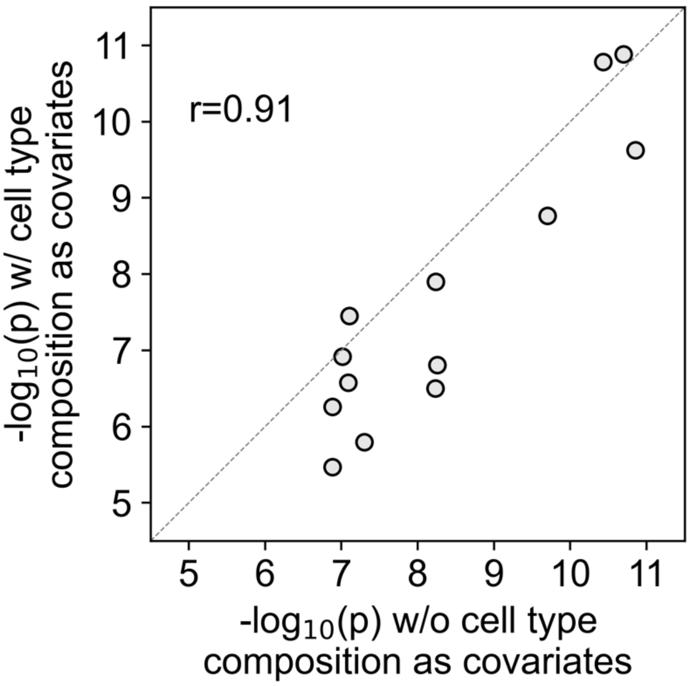

Scatter plot comparing $-\log_{10}(p)$ of COVID-19 ieQTL analysis for the 13 ieGenes (FDR <0.05), before (x axis) or after (y axis) including inferred cell type composition.

Supplementary references

1. THE GTEX CONSORTIUM. The GTEx Consortium atlas of genetic regulatory effects across human tissues. *Science* **369**, 1318–1330 (2020).
2. Kaplanis, J. *et al.* Evidence for 28 genetic disorders discovered by combining healthcare and research data. *Nature* **586**, 757–762 (2020).
3. Kanai, M. *et al. Insights from complex trait fine-mapping across diverse populations*. 2021.09.03.21262975 https://www.medrxiv.org/content/10.1101/2021.09.03.21262975v1 (2021) doi:10.1101/2021.09.03.21262975.
4. Wu, N. *et al.* TBX6 Null Variants and a Common Hypomorphic Allele in Congenital Scoliosis. *New England Journal of Medicine* **372**, 341–350 (2015).
5. Wang, Q. S. *et al.* Leveraging supervised learning for functionally informed fine-mapping of cis-eQTLs identifies an additional 20,913 putative causal eQTLs. *Nat Commun* **12**, 3394 (2021).
6. Mountjoy, E. *et al.* An open approach to systematically prioritize causal variants and genes at all published human GWAS trait-associated loci. *Nat Genet* **53**, 1527–1533 (2021).
7. Chen, M.-H. *et al.* Trans-ethnic and Ancestry-Specific Blood-Cell Genetics in 746,667 Individuals from 5 Global Populations. *Cell* **182**, 1198-1213.e14 (2020).
8. Vuckovic, D. *et al.* The Polygenic and Monogenic Basis of Blood Traits and Diseases. *Cell* **182**, 1214-1231.e11 (2020).
9. He, L. *et al.* CABLES1 Deficiency Impairs Quiescence and Stress Responses of Hematopoietic Stem Cells in Intrinsic and Extrinsic Manners. *Stem Cell Reports* **13**, 274–290 (2019).
10. ChIP-Atlas: a data-mining suite powered by full integration of public ChIP-seq data. *EMBO reports* **19**, e46255 (2018).
11. Hormozdiari, F. *et al.* Colocalization of GWAS and eQTL Signals Detects Target Genes. *The American Journal of Human Genetics* **99**, 1245–1260 (2016).
12. Wen, X., Pique-Regi, R. & Luca, F. Integrating molecular QTL data into genome-wide genetic association analysis: Probabilistic assessment of enrichment and colocalization. *PLOS Genetics* **13**, e1006646 (2017).
13. Võsa, U. *et al.* Large-scale cis- and trans-eQTL analyses identify thousands of genetic loci and polygenic scores that regulate blood gene expression. *Nat Genet* **53**, 1300–1310 (2021).
14. Westra, H.-J. *et al.* Systematic identification of trans eQTLs as putative drivers of known disease associations. *Nat Genet* **45**, 1238–1243 (2013).
15. Porcu, E. *et al.* Differentially expressed genes reflect disease-induced rather than disease-causing changes in the transcriptome. *Nat Commun* **12**, 5647 (2021).
16. Taylor-Weiner, A. *et al.* Scaling computational genomics to millions of individuals with GPUs. *Genome Biology* **20**, 228 (2019).

17. Zhang, H. *et al.* DNA methylation and allergic sensitizations: A genome-scale longitudinal study during adolescence. *Allergy* **74**, 1166–1175 (2019).
18. Ngo, H. T. T., Pham, L. V., Kim, J.-W., Lim, Y.-S. & Hwang, S. B. Modulation of Mitogen-Activated Protein Kinase-Activated Protein Kinase 3 by Hepatitis C Virus Core Protein. *Journal of Virology* **87**, 5718–5731 (2013).
19. Artigas, R., Vega-Tapia, F., Hamilton, J. & Krause, B. J. Dynamic DNA methylation changes in early versus late adulthood suggest nondeterministic effects of childhood adversity: a meta-analysis. *Journal of Developmental Origins of Health and Disease* **12**, 768–779 (2021).
20. Namkoong, H. *et al.* DOCK2 is involved in host genetics and biology of severe COVID-19. *Nature,* doi:10.1038/s41586-022-05163-5 (2022).
21. Raudvere, U. *et al.* g:Profiler: a web server for functional enrichment analysis and conversions of gene lists (2019 update). *Nucleic Acids Research* **47**, W191–W198 (2019).
22. Shang, L. *et al.* Genetic Architecture of Gene Expression in European and African Americans: An eQTL Mapping Study in GENOA. *The American Journal of Human Genetics* **106**, 496–512 (2020).
23. Aguet, F. *et al.* Genetic effects on gene expression across human tissues. *Nature* **550**, 204–213 (2017).
