# Supplementary material for: The whole blood transcriptional regulation landscape in 465 COVID-19 infected samples from Japan COVID-19 Task Force
Source: Nat Commun. 2022 Aug 22;13:4830. doi: 10.1038/s41467-022-32276-2 (PMC9395416; doi:10.1038/s41467-022-32276-2)
Supplement: Supplementary file 2 — Description of Additional Supplementary Files [file 41467_2022_32276_MOESM2_ESM.pdf]

Description of Additional Supplementary Files

File Name: Supplementary Data 1
Description: The correlation between PEER factors, other covariates and cell type composition inferred from CIBERSORT

File Name: Supplementary Data 2
Description: The list of eQTLs co-localizing with possible hematopoietic trait-causal variants (Co-localization Posterior Probability = CLPP > 0.1)

File Name: Supplementary Data 3
Description: The list of possibly causal eQTLs (PIP > 0.5) for genes suggested as relevant with COVID-19 severity in a GWAS

File Name: Supplementary Data 4
Description: The list of genes with significantly increased expression in samples with severe COVID-19 status (orange: passing the effect size threshold)

File Name: Supplementary Data 5
Description: The list of genes with significantly decreased expression in samples with severe COVID-19 status (orange: passing the effect size threshold)

File Name: Supplementary Data 6
Description: The list of genes with significantly increased expression in samples with severe COVID-19 status, when including inferred cell type composition as covariates (orange: passing the effect size threshold)

File Name: Supplementary Data 7
Description: The list of genes with significantly decreased expression in samples with severe COVID-19 status, when including inferred cell type composition as covariates (orange: passing the effect size threshold)

File Name: Supplementary Data 8
Description: . The list of regions in intron clusters with significantly increased usage in samples with severe COVID-19 status (orange: passing the effect size threshold)

File Name: Supplementary Data 9
Description: The list of regions in intron clusters with significantly decreased usage in samples with severe COVID-19 status (None of them passed the effect size threshold)

File Name: Supplementary Data 10
Description: The list of interaction eQTLs (interaction variable = COVID=19 seveirty status) passing FDR threshold
