# Supplementary material for: The whole blood transcriptional regulation landscape in 465 COVID-19 infected samples from Japan COVID-19 Task Force
Source: Nat Commun. 2022 Aug 22;13:4830. doi: 10.1038/s41467-022-32276-2 (PMC9395416; doi:10.1038/s41467-022-32276-2)
Supplement: Supplementary file 4 — Reporting Summary [file 41467_2022_32276_MOESM4_ESM.pdf]

nature portfolio

Corresponding author(s): Yukinori Okada

Last updated by author(s): Jun 13, 2022

Reporting Summary

Nature Portfolio wishes to improve the reproducibility of the work that we publish. This form provides structure for consistency and transparency in reporting. For further information on Nature Portfolio policies, see our Editorial Policies and the Editorial Policy Checklist.

Statistics

For all statistical analyses, confirm that the following items are present in the figure legend, table legend, main text, or Methods section.

n/a	Confirmed	
☐	☒	The exact sample size (*n*) for each experimental group/condition, given as a discrete number and unit of measurement
☐	☒	A statement on whether measurements were taken from distinct samples or whether the same sample was measured repeatedly
☐	☒	The statistical test(s) used AND whether they are one- or two-sided<br>*Only common tests should be described solely by name; describe more complex techniques in the Methods section.*
☐	☒	A description of all covariates tested
☐	☒	A description of any assumptions or corrections, such as tests of normality and adjustment for multiple comparisons
☐	☒	A full description of the statistical parameters including central tendency (e.g. means) or other basic estimates (e.g. regression coefficient) AND variation (e.g. standard deviation) or associated estimates of uncertainty (e.g. confidence intervals)
☐	☒	For null hypothesis testing, the test statistic (e.g. *F*, *t*, *r*) with confidence intervals, effect sizes, degrees of freedom and *P* value noted<br>*Give P values as exact values whenever suitable.*
☐	☒	For Bayesian analysis, information on the choice of priors and Markov chain Monte Carlo settings
☒	☐	For hierarchical and complex designs, identification of the appropriate level for tests and full reporting of outcomes
☐	☒	Estimates of effect sizes (e.g. Cohen's *d*, Pearson's *r*), indicating how they were calculated

Our web collection on statistics for biologists contains articles on many of the points above.

Software and code

Policy information about availability of computer code

Data collection	No software was used in data collection.
Data analysis	The code used in this manuscript is available at https://github.com/QingboWang/japan_covid_taskforce_rna .<br><br>The set of softwares and tools used for the analysis as well as data visualization are listed as below;<br>CIBERSORT web interface (http://cibersort.stanford.edu/)<br>DESeq2 v1.32.0 (https://bioconductor.org/packages/release/bioc/html/DESeq2.html)<br>edgeR v3.34 (https://bioconductor.org/packages/release/bioc/html/edgeR.html)<br>fastQTL v2.165 (http://fastqtl.sourceforge.net)<br>FINEMAP v1.3.1 (http://www.christianbenner.com/)<br>GATK v4.1.9.0 LiftoverVcf (https://gatk.broadinstitute.org/)<br>ggsashimi v.1.1.0 (https://github.com/guigolab/ggsashimi)<br>g:Profiler web interface (https://biit.cs.ut.ee/gprofiler/page/citing)<br>GTEx pipeline (https://github.com/broadinstitute/gtex-pipeline)<br>LeafCutter v0.2.7 (https://davidaknowles.github.io/leafcutter/index.html)<br>matplotlib v3.3.4 (https://matplotlib.org)<br>numpy v1.20.1 (https://numpy.org)<br>pandas v1.1.4 (https://pandas.pydata.org)<br>RSEM v1.3.0 (https://deweylab.github.io/RSEM/)<br>scikit-learn v0.24.1 (https://scikit-learn.github.io/stable)<br>scipy v1.6.2 (http://scikit-learn.github.io/stable)<br>seaborn v0.11.1 (https://seaborn.pydata.org)<br>STAR v2.5.3a and v2.6.0 (https://github.com/alexdobin/STAR)

susieR v0.11.43 (https://github.com/stephenslab/susieR)
tensorQTL v1.0.5 (https://github.com/broadinstitute/tensorqtl)
Variant Effect Predictor (VEP) version 104 web interface (https://asia.ensembl.org/Homo_sapiens/Tools/VEP/)

For manuscripts utilizing custom algorithms or software that are central to the research but not yet described in published literature, software must be made available to editors and reviewers. We strongly encourage code deposition in a community repository (e.g. GitHub). See the Nature Portfolio guidelines for submitting code & software for further information.

Data

Policy information about availability of data

All manuscripts must include a data availability statement. This statement should provide the following information, where applicable:

- Accession codes, unique identifiers, or web links for publicly available datasets
- A description of any restrictions on data availability
- For clinical datasets or third party data, please ensure that the statement adheres to our policy

The summary statistics of eQTL (cis and trans) and cis-sQTL analysis, as well as the RNA-seq expression matrix are available at the National Bioscience Database Center (NBDC) Human Database (accession code: hum0343).
The individual genotype data is available at European Genome-Phenome Archive (EGA) with the accession code EGAS00001006284.
The data from colocalization, differential expression and ieQTL analysis in this study are provided I the Supplementary Data file.

The list of publicly available datasets used are listed below:
Biobank Japan (BBJ) and UK Biobank (UKB) fine-mapping:
NBDC Human Database (accession code: hum0197) and https://www.finucanelab.org/data
eQTLgen trans-eQTL data: https://www.eqtlgen.org/trans-eqtls.html
The expression modifier score (EMS): https://www.finucanelab.org/data
Genome Aggregation Database (gnomAD) allele frequencies: https://gnomad.broadinstitute.org/downloads
Genotype-Tissue Expression (GTEx) cis-eQTL data: https://gtexportal.org/home/datasets
ImmuNexUT cell type expression data: https://www.immunexut.org
Multi-Ethnic Study of Atherosclerosis (MESA) cis-eQTL data: https://www.dropbox.com/sh/f6un5evevyvvyl9/AAA3sfa1DgqY67tx4q36P341a?dl=0

Field-specific reporting

Please select the one below that is the best fit for your research. If you are not sure, read the appropriate sections before making your selection.

☒ Life sciences ☐ Behavioural & social sciences ☐ Ecological, evolutionary & environmental sciences

For a reference copy of the document with all sections, see nature.com/documents/nr-reporting-summary-flat.pdf

Life sciences study design

All studies must disclose on these points even when the disclosure is negative.

Sample size	The study participants were recruited through Japan COVID-19 Task Force. Whole blood-RNA-sequencing was performed for a subset of the genotyped samples (n=500) and analyzed in this study. Although no sample size was predetermined due to the unpredictable nature of the COVID-19 outbreak, the current sample size is comparable with major bulk RNA-seq studies such as GTEx (n = 670 for whole blood).
Data exclusions	Stringent sample and variant level quality control (QC) filters were applied (sample call rate > 0.97, variant call rate > 0.99 for DNA-seq, number of mapped RNA read > 10^7, mapping rate>0.8, intergenic rate<0.1, base mismatch rate<0.01, ribosomal RNA rate<0.3, and intersample correlation deviation score >-15), resulting in n=465 samples for main analyses. The distribution of the quality are available at Supplementary Fig. 1
Replication	Although we did not attempt to replicate our results by constructing another dataset of the same nature due to its uniqueness, we replicated our main findings by comparing with existing databases such as GTEx (cis-e/sQTL study), UK Biobank (co-localization study), and eQTLgen (trans-eQTL study), once for each.
Randomization	We did not need to use randomization in this study because this is a genotype-gene expression association study. All the samples with available accessibility to genotype and RNA expression data passing quality control threshold were included in the analysis.
Blinding	We did not apply blinding of the samples because this is a genotype-gene expression association study and no intervention was conducted in our study.

Reporting for specific materials, systems and methods

We require information from authors about some types of materials, experimental systems and methods used in many studies. Here, indicate whether each material, system or method listed is relevant to your study. If you are not sure if a list item applies to your research, read the appropriate section before selecting a response.

Materials & experimental systems

n/a	Involved in the study
☒	☐ Antibodies
☒	☐ Eukaryotic cell lines
☒	☐ Palaeontology and archaeology
☒	☐ Animals and other organisms
☐	☒ Human research participants
☒	☐ Clinical data
☒	☐ Dual use research of concern

Methods

n/a	Involved in the study
☒	☐ ChIP-seq
☒	☐ Flow cytometry
☒	☐ MRI-based neuroimaging

Human research participants

Policy information about studies involving human research participants

Population characteristics	Study participants are of East Asian ancestry, the mean age was 59, 68% were male, and all of them were tested positive for PCR test results.
Recruitment	We enrolled participants diagnosed as COVID-19 positive by physicians using the clinical manifestation and PCR test results at one of the >100 the affiliated hospitals participating to Japan COVID-19 Task Force. Any subjects with obtained informed consent were included without further biases.
Ethics oversight	This study was approved by the ethical committees of Keio University School of Medicine, Osaka University Graduate School of Medicine, and affiliated institutes. Informed consent was obtained from all participants.

Note that full information on the approval of the study protocol must also be provided in the manuscript.
